# Bone marrow sinusoidal endothelium controls terminal erythroid differentiation and reticulocyte maturation

**DOI:** 10.1038/s41467-021-27161-3

**Published:** 2021-11-29

**Authors:** Joschka Heil, Victor Olsavszky, Katrin Busch, Kay Klapproth, Carolina de la Torre, Carsten Sticht, Kajetan Sandorski, Johannes Hoffmann, Hiltrud Schönhaber, Johanna Zierow, Manuel Winkler, Christian David Schmid, Theresa Staniczek, Deborah E. Daniels, Jan Frayne, Georgia Metzgeroth, Daniel Nowak, Sven Schneider, Michael Neumaier, Vanessa Weyer, Christoph Groden, Hermann-Josef Gröne, Karsten Richter, Carolin Mogler, Makoto Mark Taketo, Kai Schledzewski, Cyrill Géraud, Sergij Goerdt, Philipp-Sebastian Koch

**Affiliations:** 1 Department of Dermatology, Venereology and Allergology, University Medical Center and Medical Faculty Mannheim, Heidelberg University, and Center of Excellence in Dermatology, Mannheim, Germany; 2 European Center for Angioscience (ECAS), Medical Faculty Mannheim, Heidelberg University, Mannheim, Germany; 3 Division of Cellular Immunology, German Cancer Research Center (DKFZ), Heidelberg, Germany; 4 Department of Immunobiochemistry, Mannheim Institute for Innate Immunoscience (MI3), Medical Faculty Mannheim, Heidelberg University, Mannheim, Germany; 5 Core Facility Platform Mannheim (CFPM), NGS Core Facility, Medical Faculty Mannheim, Heidelberg University, Mannheim, Germany; 6 School of Biochemistry, University of Bristol, Bristol, UK; 7 Department for Hematology and Oncology, University Medical Center and Medical Faculty Mannheim, Heidelberg University, Mannheim, Germany; 8 Institute for Clinical Chemistry, University Medical Center and Medical Faculty Mannheim, Heidelberg University, Mannheim, Germany; 9 Department of Neuroradiology, University Medical Center and Medical Faculty Mannheim, Heidelberg University, Mannheim, Germany; 10 Department of Radiation Oncology, University Medical Center and Medical Faculty Mannheim, Heidelberg University, Mannheim, Germany; 11 Institute of Pharmacology, Philipps-University, Marburg, Germany; 12 Division of Molecular Genetics, German Cancer Research Center (DKFZ), Heidelberg, Germany; 13 Institute of Pathology, Technical University Munich, Munich, Germany; 14 Division of Experimental Therapeutics, Graduate School of Medicine, Kyoto University, Kyoto, Japan; 15 Section of Clinical and Molecular Dermatology, Department of Dermatology, Venereology and Allergy, University Medical Center and Medical Faculty Mannheim, Heidelberg University, Mannheim, Germany

**Keywords:** Myelodysplastic syndrome, Stem-cell niche, Molecular medicine

## Abstract

Within the bone marrow microenvironment, endothelial cells (EC) exert important functions. Arterial EC support hematopoiesis while H-type capillaries induce bone formation. Here, we show that BM sinusoidal EC (BM-SEC) actively control erythropoiesis. Mice with stabilized β-catenin in BM-SEC (*Ctnnb1*^*OE-SEC*^) generated by using a BM-SEC-restricted Cre mouse line (*Stab2-iCreF3*) develop fatal anemia. While activation of Wnt-signaling in BM-SEC causes an increase in erythroblast subsets (PII–PIV), mature erythroid cells (PV) are reduced indicating impairment of terminal erythroid differentiation/reticulocyte maturation. Transplantation of *Ctnnb1*^*OE-SEC*^ hematopoietic stem cells into wildtype recipients confirms lethal anemia to be caused by cell-extrinsic, endothelial-mediated effects. *Ctnnb1*^*OE-SEC*^ BM-SEC reveal aberrant sinusoidal differentiation with altered EC gene expression and perisinusoidal ECM deposition and angiocrine dysregulation with de novo endothelial expression of FGF23 and DKK2, elevated in anemia and involved in vascular stabilization, respectively. Our study demonstrates that BM-SEC play an important role in the bone marrow microenvironment in health and disease.

## Abstract (web summary)

Niche crosstalk with Haematopoietic cells underlies normal haematopoiesis and myeloid disorders. Here the authors report a Stabilin2-Cre driver mouse with Cre-activity restricted to bone marrow sinusoidal endothelial cells, and that Stabilin2-Cre driven overactivation of b-catenin leads to erythroid differentiation defects and anaemia.

## Introduction

Crosstalk between hematopoietic stem and progenitor cells (HSPCs) and cells of the bone marrow (BM) microenvironment is increasingly recognized to regulate normal hematopoiesis as well as development of myeloid disorders^1^. Multiple cell types contribute to the BM niche, including mesenchymal stromal cell populations (MSC) and their progeny such as osteoblasts and perivascular stroma cells, but also macrophages as well as endothelial cells (ECs)^2,3^. The murine BM vasculature displays remarkable angiodiversity. Distinct vessel types within the BM niche comprise arterial endothelial cells (AEC), H-type capillary vessels, and BM sinusoidal (L-type) endothelial cells (BM-SEC) that contribute to osteogenesis, bone angiogenesis, and HSPC maintenance and differentiation via angiocrine signaling^4–9^. EC-derived stem cell factor (Scf) is selectively secreted by AEC, and genetic deletion of Scf in AEC, but not BM-SEC, significantly reduces functional HSPC^10^. H-type capillary vessels contribute to bone formation, vascularization, and regeneration via Notch signaling^11^. BM-SEC are the most abundant EC population in the BM, activate HSPC, and regulate BM trafficking^4^. E-type ECs and Apelin-expressing ECs are additional, specialized BM-EC subpopulations that control vascular development, or vascular regeneration and hematopoiesis after myeloablative injury, respectively^12,13^. The recent improvements of single-cell transcriptomics and refined flow cytometry sorting techniques have given new insights into the molecular profile of the BM vascular niche and the BM microenvironment (BMME), thereby identifying decisive gene signatures for AEC and BM-SEC^3,10,14^. The balance between AEC, H-type vessels and BM-SEC fluctuates; upon ageing, H-type vessels regress entailing reduced hematopoiesis and osteogenesis^6^. Notably, endothelial Notch activation can revert this process inducing arterialization and H-type capillary expansion^4,11^.

Despite these essential data on the involvement of BM-EC in normal hematopoiesis, evidence for the involvement of BM-EC in the pathogenesis of hematological disorders is scarce. Notably, emerging data suggest that alterations of the BM niche as opposed to cell-intrinsic factors do play a role in the pathogenesis of myeloid neoplasms^15^. Patient-derived MSC were demonstrated to exhibit cytogenetic abnormalities that are distinct from their hematopoietic counterparts and to promote the malignant behavior of stem cells and their progeny in human myelodysplastic syndrome (MDS)^16^. In mice, for example, deletion of retinoic acid receptor-γ^17^ and activation or derepression of Wnt signaling in the BMME^18,19^ have been shown to promote the development of myeloproliferation or myelodysplasia. Regarding Wnt signaling, osteoblasts genetically engineered to specifically express a constitutively active mutant allele of *Ctnnb1* using the *α1(I)Collagen-Cre* driver develop acute myeloid leukemia due to the upregulation of the Notch ligand Jagged 1^18^. In the Apc haploinsufficiency MDS model in which Wnt signaling in the BMME causes ineffective hematopoiesis, the *Mx1-Cre* driver was used to target MSC^20^. Conflicting results, however, have been reported regarding targeting of other niche cells, especially EC by *Mx1-Cre*^21–23^. In analyzing published data on the regulation of HSPC interactions with the BM niche by Wnt-β-catenin signaling, Lane and Heidel therefore suggested to further dissect the specific contributions of MSC or EC potentially mediating Wnt-dependent myelodysplasia^24^. In order to be able to explore the distinct functions of these latter cellular participants within the BMME in vivo, however, the lack of suitable, highly specific *Cre*-driver mice constitutes a major obstacle. Unfortunately, most EC-targeted driver *Cre* mice target all EC as well as hematopoietic stem cells and part of their progeny^22^, and the *EpoR-Cre* driver used to target BM-SEC is expressed also in erythroid cells^10^.

In order to comprehensively analyze the effects of BM-SEC-restricted activation of Wnt signaling within the BM niche, we generated a *Cre* driver mouse under the control of the *Stabilin-2* (*Stab2*) promotor. *Stab2* is known to be specifically expressed by BM-SEC, but also by SEC of other organs, and recent studies confirmed *Stab2* to be the gene most strongly overexpressed in BM-SEC versus AEC^10,14^. In contrast to the *Stab2-iCre* Founder 2 mouse (*Stab2-iCreF2*) reported previously^25,26^, the *Stab2-iCre* Founder 3 (*Stab2-iCreF3*) line shows restricted *Cre* activity in BM-SEC without reporter activity in HSPC and AEC. Here, we show that BM-SEC genetically engineered to specifically express a constitutively active mutant allele of *Ctnnb1* using the *Stab2-iCreF3* driver mouse line (*Stab2-iCreF3*^*tg/wt*^ *Ctnnb1(Ex3)*^*fl/wt*^) exhibit aberrant sinusoidal differentiation and cause lethal anemia due to a block in terminal erythroid differentiation.

## Results

### Generation and characterization of *Stab2-iCreF3* mice

**Fig. 1:**
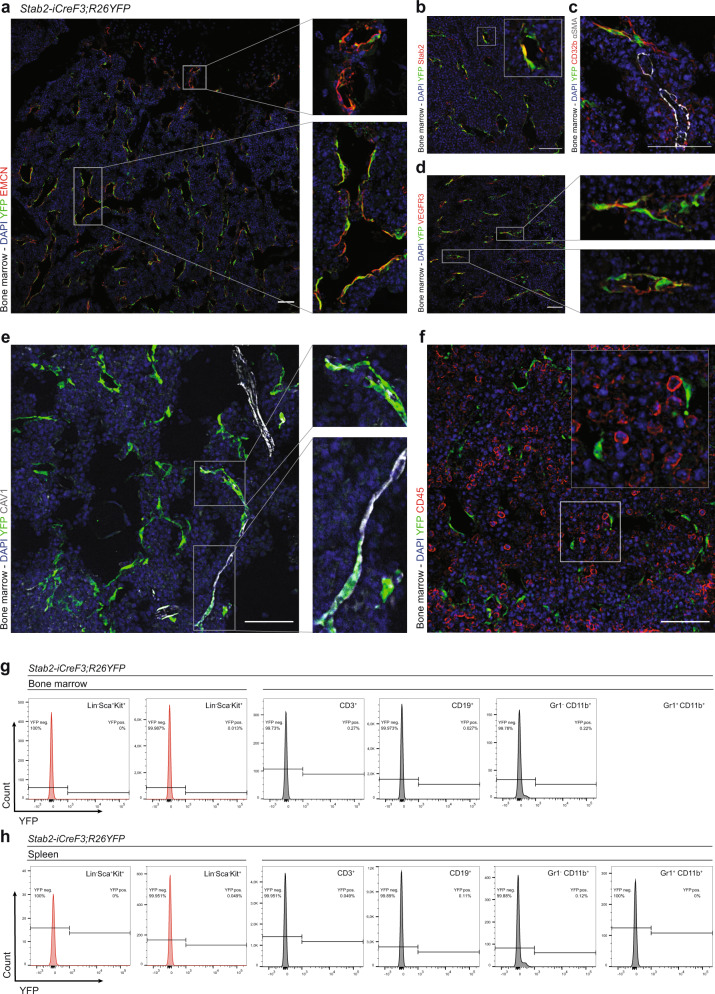
*Stab2-iCreF3;R26YFP* mice show reporter activity restricted to sinusoidal endothelial cells in the bone marrow. **a**–**c** Immunofluorescence (IF) staining for endothelial markers of *Stab2-iCreF3;R26YFP* bone marrow from 6-month-old female mice. Co-IF of YFP, DAPI and **a** EMCN (*n* = 3), **b** Stab2 (*n* = 3), **c** CD32b, α-SMA (*n* = 3), **d** VEGFR3, and **e** CAV1. Scale bars: 50 μm. **f**–**h** Hematopoietic marker staining of *Stab2-iCreF3;R26YFP* bone marrow from 5-month-old female mice for IF and flow cytometry analysis. **f** Co-IF of YFP, DAPI, CD45 (*n* = 3). Scale bars: 50 μm. Representative graphs of flow cytometry YFP quantification in **g** Lin^-^Sca-1^+^Kit^+^, Lin^-^Sca-1^-^Kit^+^ cells, T cells (CD3^+^), B cells (CD19^+^), myeloid cells (Gr1^-^CD11b^+^, Gr1^+^CD11b^+^) of **g** bone marrow and **h** spleen (*n* = 4). Source data for **g**, **h** are provided as a Source Data file.

To study the effects of β-catenin overactivation in BM-SEC, an *iCre* driver mouse line was generated using the promoter of the *Stabilin2* (*Stab2*) gene specifically expressed in SECs of the BM, the liver, the spleen and the lymph nodes (*Stab2-iCreF3*). In contrast to the *Stab2-iCreF2* driver mice generated by us previously^25^, the reporter activity of *Stab2-iCreF3;R26YFP* mice was restricted to SECs with endothelial Stab2 expression in adult animals^27,28^. In comparison to SEC in liver, spleen, and lymph nodes (Supplementary Fig. 1a), BM-SEC exhibited a more homogenous reporter activity (Fig. 1a). Organs without SEC such as the lungs, kidney, heart, or intestines did not show notable reporter activity (Supplementary Fig. 1b). Reporter-positive BM-SEC were characterized as Emcn^+^Stab2^+^CD32b^+^VEGFR3^+^ EC (Fig. 1a–d and Supplementary Fig. 1c), while CAV1^+^ AEC and CD32b^-^α-SMA^+^ larger arteries were largely reporter YFP negative (Fig. 1c, e). Only scattered CAV1^+^YFP^+^ vessels were found, presumably representing connections between AEC and BM-SEC (Fig. 1e). Importantly, CD45^+^ (Fig. 1f) hematopoietic cells were reporter-negative. Flow cytometry analysis (FACS) of whole BM and spleen showed no reporter positivity in hematopoietic stem cells or their progeny (Fig. 1g, h), as compared to our previously published *Stab2-iCreF2;R26YFP* mouse line (Supplementary Fig. 1d) that showed reporter positivity in 5–10% of hematopoietic stem cells and their progeny^25^. Developmentally, reporter activity in BM-EC was first observed starting at 10 days of age (P10), whereas embryonic day (E)17.5 embryos and P0 pups were YFP negative (Supplementary Fig. 1e). In the fetal liver, no reporter activity was found at E17.5, but discrete YFP-positivity was present at day P1 in some liver EC, while at day P10 several liver ECs were YFP-positive (Supplementary Fig. 1f). Taken together, these results confirmed highly specific Cre-activity in the postnatal sinusoidal vasculature within the BM niche.

### Activation of β-catenin in BM-SEC causes lethal anemia

**Fig. 2:**
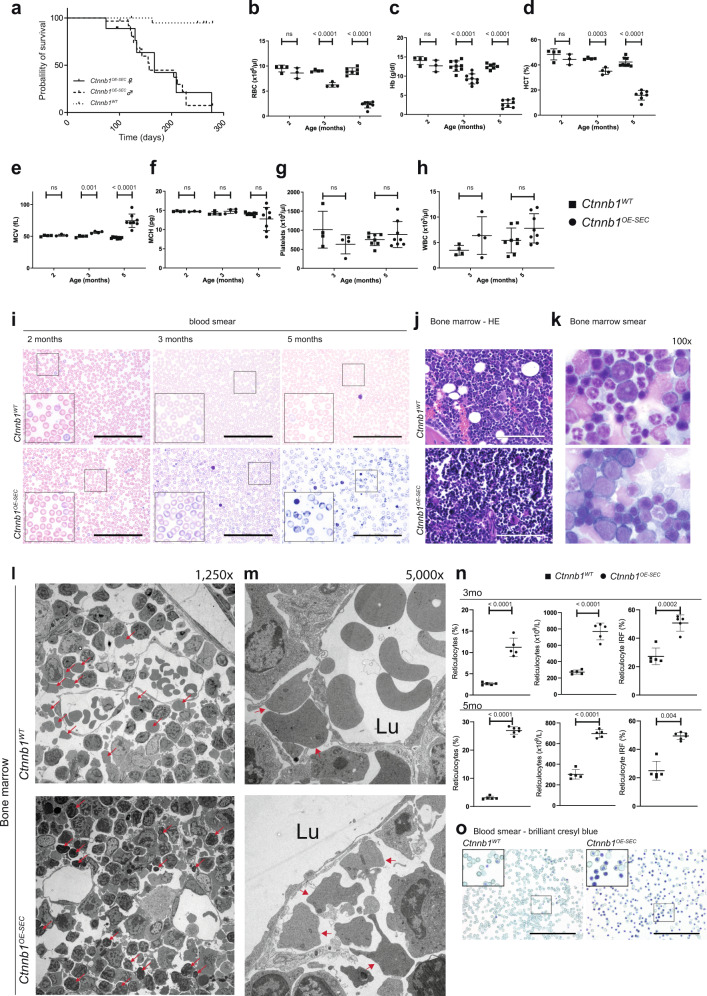
Activation of β-catenin in BM-SECs (*Ctnnb1*^*OE-SEC*^) causes fatal anemia. **a** Kaplan–Meier survival curves for control (*Ctnnb1*^*WT*^) and *Ctnnb1*^*OE-SEC*^ mice. The probability of survival is shown for *Ctnnb1*^*OE-SEC*^ female (*n* = 18) vs. *Ctnnb1*^*OE-SEC*^ male (*n* = 30) vs. *Ctnnb1*^*WT*^ mice (*n* = 25). **b**–**f** Red blood cell indices for 2-, 3-, and 5-month-old male *Ctnnb1*^*WT*^ and *Ctnnb1*^*OE-SEC*^ mice. **b** Red blood cells (RBC) (2 months, *n* ≥ 3; 3 months, *n* = 4; 5 months, *n* ≥ 7), **c** hemoglobin (Hb) (2 months, *n* ≥ 3; 3 months, *n* = 9; 5 months, *n* ≥ 7), **d** hematocrit (HCT) (2 months, *n* ≥ 3; 3 months, *n* = 4; 5 months, *n* ≥ 7), **e** mean corpuscular volume (MCV) (2 months, *n* ≥ 3; 3 months, *n* = 4; 5 months, *n* = 8), **f** mean corpuscular hemoglobin (MCH) (2 months, *n* ≥ 3; 3 months, *n* = 4; 5 months, *n* = 8). **g** Platelet (3 months, *n* = 4; 5 months, *n* = 8) and **h** white blood cell (WBC) counts (3 months, *n* = 4; 5 months, *n* = 8) for 3- and 5-month-old male *Ctnnb1*^*WT*^ and *Ctnnb1*^*OE-SEC*^ mice. **i** Peripheral blood smears of 2- and 3-month-old female and 5-month-old male *Ctnnb1*^*WT*^ and *Ctnnb1*^*OE-SEC*^ (*n* ≥ 3) mice. Scale bars: 100 μm. **j** H&E staining of bone marrow sections of 3-month-old female *Ctnnb1*^*WT*^ and *Ctnnb1*^*OE-SEC*^ (*n* = 5). Scale bar: 100 μm. **k** Bone marrow smears of 5-month-old male *Ctnnb1*^*WT*^ and *Ctnnb1*^*OE-SEC*^ (*n* = 3) mice (×100 enlargement). **l**, **m** Representative transmission electron microscopy analysis of *Ctnnb1*^*WT*^ and *Ctnnb1*^*OE-SEC*^ bone marrow. **l** Arrows indicate erythroblasts, reticulocytes in the bone marrow parenchyma (×1250 enlargement). **m** Arrows indicate reticulocytes outside of sinusoidal walls (×5000 enlargement). 3- and 4-month-old female *Ctnnb1*^*WT*^ and *Ctnnb1*^*OE-SEC*^ mice (*n* = 3). **n**, **o** Reticulocyte analysis in the peripheral blood. **n** Relative (%), absolute (×10^9^/L) reticulocyte numbers, and immature reticulocyte fraction (IRF) in the peripheral blood of 3- and 5-month-old female *Ctnnb1*^*WT*^ and *Ctnnb1*^*OE-SEC*^ mice (*n* ≥ 5). **o** Brilliant cresyl blue staining of blood smears of 5-month-old male *Ctnnb1*^*WT*^ and *Ctnnb1*^*OE-SEC*^ mice (*n* = 3). Scale bar: 100 μm. The dots in the graphs represent individual mice. Mean ± s.e.m. is shown for each group of mice in all graphs. Statistical significance was determined using Student’s *t*-test or Mann–Whitney *U* test (two-sided). ns, not significant. Source data, precise values of *n*, and employed statistical tests for **a**, **b**, **c**, **d**, **e**, **f**, **g**, **h**, **n** are provided as a Source Data file.

β-catenin overactivation in BM-SEC was achieved by crossing *Stab2-iCreF3*^*tg/wt*^ with *Ctnnb1(Ex3)*^*fl/fl*^ mice^29^ (*Stab2-iCreF3*^*tg/wt*^ *Ctnnb1(ex3)*^*fl/wt*^ [*Ctnnb1*^*OE-SEC*^]) (Supplementary Fig. 2a). *Ctnnb1*^*OE-SEC*^ mice were born at a lower Mendelian frequency than expected (Supplementary Fig. 2b) and suffered from reduced overall survival rates with a mean survival time of 162 days (167 days for females; 157 days for males; *p* < 0.0001 for both sexes) (Fig. 2a). *Ctnnb1*^*OE-SEC*^ mice developed severe anemia at the age of 3 months as demonstrated by significantly lower red blood cell counts, hemoglobin levels, and hematocrit (Fig. 2b–d). The mean red cell volume was significantly increased, while the mean corpuscular hemoglobin was not significantly changed at most time points (Fig. 2e, f) indicating macrocytic, normochromic anemia in *Ctnnb1*^*OE-SEC*^ mice. Serum analysis showed increased serum iron levels and no aberrant LDH or K^+^ levels in *Ctnnb1*^*OE-SEC*^ mice (Supplementary Fig. 2c–e). White blood cell counts and platelets were normal (Fig. 2g, h and Supplementary Fig. 2f, g). Peripheral blood smears of *Ctnnb1*^*OE-SEC*^ mice revealed striking polychromasia starting at 2–3 months of age indicating premature release of red blood cells in the bloodstream; in addition, scattered fragmentocytes were found (Fig. 2i). BM histology (Fig. 2j) and smears (Fig. 2k and Supplementary Fig. 2h) showed an increased number of immature erythroid precursors in mutant *Ctnnb1*^*OE-SEC*^ mice. Transmission electron microscopy (TEM) of the BM of *Ctnnb1*^*OE-SEC*^ mice further revealed accumulation of early erythroid progenitors and irregularly shaped reticulocytes outside the lumina of the sinusoids and an obvious lack of reticulocytes and mature erythrocytes beneath and inside the sinusoidal lumina (Fig. 2l, m). Further peripheral blood analysis showed significantly more mature and numerous immature reticulocytes with nuclear remnants in the circulation in mutant *Ctnnb1*^*OE-SEC*^ mice (Fig. 2n, o).

### *Ctnnb1*^*OE-SEC*^ mice develop extramedullary hematopoiesis

The anemic *Ctnnb1*^*OE-SEC*^ mice developed excessive splenomegaly with strongly increased spleen weight as well as increased spleen to bodyweight ratio (Supplementary Fig. 3a, b). Splenomegaly, suggestive of extramedullary hematopoiesis, was first noticeable in 2-month-old female *Ctnnb1*^*OE-SEC*^ mice and showed aggravation at older ages in both female and male mice (Supplementary Fig. 3b). Spleen cellularity showed a tendency toward higher numbers at the age of 3 months, while the cell number was significantly increased at the age of 5 months (Supplementary Fig. 3c). Microscopically, the spleen demonstrated a complete loss of its histological structure with effacement of the white pulp (Supplementary Fig. 3d), as confirmed by reduced numbers of B and T cells in the spleen (Supplementary Fig. 3e). In contrast, we observed higher B- and T-cell numbers in the peripheral blood of *Ctnnb1*^*OE-SEC*^ mice (Supplementary Fig. 3f). FACS revealed increased numbers of HSPCs (HSC, ST-HSC), of megakaryocyte-erythroid progenitor cells (MEP), granulocyte-monocyte progenitor cells (GMP), and of common myeloid progenitor cells (CMP) suggestive of extramedullary hematopoiesis (Supplementary Fig. 3g). Significantly more colony-forming unit (CFU)-GEMM colony numbers further indicated extramedullary hematopoiesis (Supplementary Fig. 3h), while unaltered *Bmp4* expression excludes classical BMP4-dependent splenic stress erythropoiesis^30^ (Supplementary Fig. 3i). Furthermore, immunofluorescent analysis revealed increased numbers of DAPI-stained nucleated Ter119^+^ erythroid progenitors and hematopoietic Kit^+^ progenitor cells (Supplementary Fig. 3j).

Extramedullary hematopoiesis was also detected in the liver albeit to a lesser extent. Despite only marginal increases in liver weight and liver to bodyweight ratio (Supplementary Fig. 4a, b), histological and immunofluorescent analyses of liver sections revealed small erythroblastic islands throughout the liver parenchyma (Supplementary Fig. 4c, d). In addition, increased numbers of CD71^+^Ter119^+^ cells were found in the liver of *Ctnnb1*^*OE-SEC*^ mice as analyzed by flow cytometry (Supplementary Fig. 4e). On the contrary, hepatic sinusoidal endothelial zonation^31^ as well as angiocrine Wnt ligand-mediated metabolic liver zonation^32^ did not reveal any abnormalities as analyzed by expression of Lyve1 and EMCN or glutamine synthetase and Arginase 1, respectively (Supplementary Fig. 4f).

### Activation of β-catenin in BM-SEC impairs erythropoiesis

**Fig. 3:**
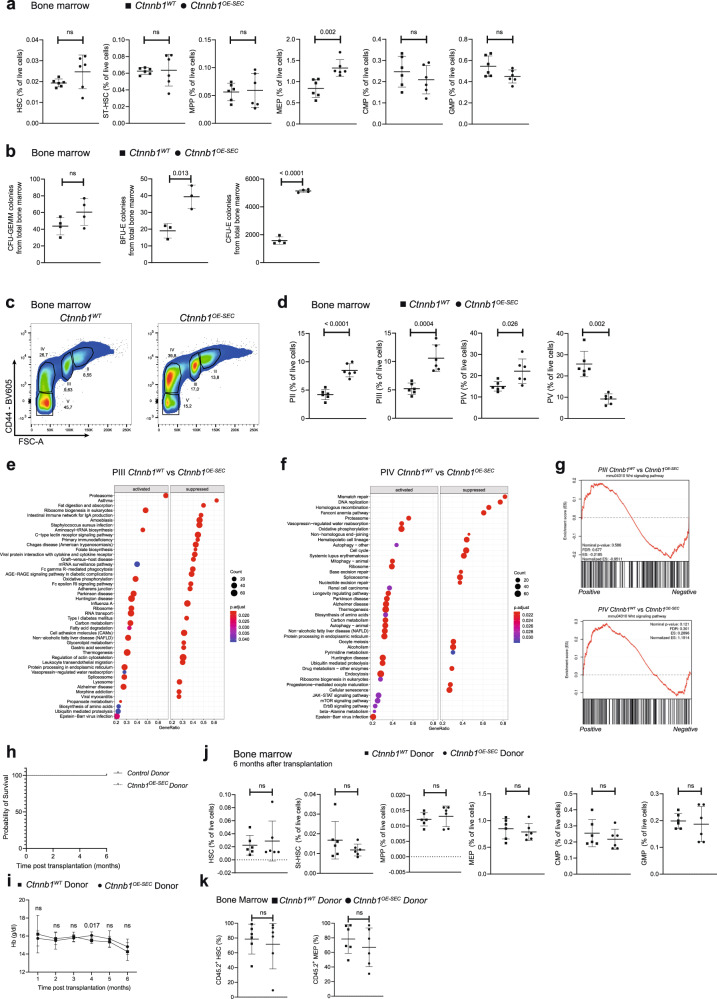
The erythroid cells of *Ctnnb1*^*OE-SEC*^ mice show a block in differentiation at the level of orthochromatic erythroblasts and immature reticulocytes. **a** Flow cytometry quantification (% of live cells) of HSCs (Lin^-^Sca-1^+^Kit^+^CD150^+^CD48^-^), short-term HSCs (ST-HSCs) (Lin^-^Sca-1^+^Kit^+^CD150^-^CD48^-^), MPPs (Lin^-^Sca-1^+^Kit^+^CD150^-^CD48^+^), GMPs (Lin^-^Sca-1^-^Kit^+^CD34^+^CD16/32^+^), CMPs (Lin^-^Sca-1^-^Kit^+^CD34^+^CD16/32^-^), and MEPs (Lin^-^Sca-1^-^Kit^+^CD34^-^CD16/32^-^) of 3-month-old *Ctnnb1*^*WT*^ and *Ctnnb1*^*OE-SEC*^ females (*n* = 6). **b** Numbers of in vitro CFU assays performed with total bone marrow cells from 3-month-old *Ctnnb1*^*WT*^ and *Ctnnb1*^*OE-SEC*^ females (CFU-GEMM and CFU-E, *n* = 4; BFU-E, *n* = 3). Experiments were performed in triplicates. **c** Representative image of flow cytometry analysis of erythroid bone marrow cells. Plots of CD44 versus FSC of Ter119+-pre-gated BM cells were subdivided into four distinct populations, PII–PV. **d** Flow cytometry quantification (% of live cells) of erythroid populations PII–PV from of bone marrow cells of 3-month-old *Ctnnb1*^*WT*^ and *Ctnnb1*^*OE-SEC*^ females (*n* = 6). **e**, **f** GSEA-KEGG pathway alterations analyzed using MSigDB hallmark gene sets in isolated **e** PIII and **f** PIV cells from bone marrow of 5-month-old male *Ctnnb1*^*WT*^ and *Ctnnb1*^*OE-SEC*^ (*n* = 5). **g** GSEA plots of Wnt signaling pathway in PIII (upper panel) and PIV (lower panel) cells. A normalized enrichment scores (NES) was calculated after normalization across all analyzed gene sets. The red curve corresponds to the ES (enrichment score) curve, which is the running sum of the weighted enrichment score. **h**–**k** Transplantation of 1200 HSC from CD45.2 *Ctnnb1*^*WT*^ and *Ctnnb1*^*OE-SEC*^ mice into lethally irradiated CD45.1 C57BL/6J recipients. **h** Survival curves of mice following transplantation with *Ctnnb1*^*WT*^ and *Ctnnb1*^*OE-SEC*^ donor HSCs (*n* = 6 per group). **i** Monthly Hb measurement of CD45.1 C57BL/6J recipients transplanted with *Ctnnb1*^*WT*^ and *Ctnnb1*^*OE-SEC*^ donor HSCs (*n* = 6). **j** Flow cytometry quantification of HSC, ST-HSC, MPP, GMP, CMP, and MEP of transplanted CD45.1 C57BL/6J recipients at 6 months after transplantation (*n* = 6). **k** Flow cytometry quantification of CD45.2 positivity in HSC and MEP in the bone marrow of CD45.1 C57BL/6J recipients transplanted with *Ctnnb1*^*OE-SEC*^ and *Ctnnb1*^*WT*^ HSC CD45.2 donor at 6 months after transplantation (*n* = 6). The dots in the graphs represent individual mice. Mean ± s.e.m. is shown for each group of mice in all graphs. Statistical significance was determined using Student’s *t*-test or Mann–Whitney *U* test (two-sided). ns, not significant. Source data, precise values of *n* and employed statistical tests for **a**, **b**, **d**, **e**, **f**, **g**, **h**, **i**, **j**, **k** are provided as a Source Data file.

A detailed FACS of the BM of *Ctnnb1*^*OE-SEC*^ mice revealed significantly increased numbers (frequency) of MEP in the BM, while numbers of HSC, ST-HSC, multipotent progenitors (MPP), GMP, and CMP were normal compared to *Ctnnb1*^*WT*^ littermates (Fig. 3a). Notably, no significant alterations in total BM cellularity of *Ctnnb1*^*OE-SEC*^ and control mice were found (Supplementary Fig. 5a). Less CD11b^+^Gr1^+^ and Ly6G^+^ cells (mostly neutrophils and myeloid-derived suppressor cells) were present in the BM of *Ctnnb1*^*OE-SEC*^ mice, while no differences were observed for CD11b^+^Gr1^-^ cells (mostly monocytes). Ly6C^+^CD115^+^ cells (mostly monocytes/macrophages) were significantly increased in the BM of our mutant mice (Supplementary Fig. 5b, c).

The numbers of CD3^+^ T cells and CD19^+^ B cells were significantly decreased in the BM (Supplementary Fig. 5d). When analyzing Pro-B, Pre-B, immature B cells, and mature B cells in relation to differentiated B cells, the ratio of Pre-B was reduced, while Pro-B cells and mature B cells were increased (Supplementary Fig. 5e).

Due to the fatal anemia in *Ctnnb1*^*OE-SEC*^ mice, we analyzed erythroid differentiation in more detail. Colony assays of cells isolated from total BM revealed no difference in CFU-GEMM numbers, but displayed significantly higher numbers of BFU-E and CFU-E (Fig. 3b). Erythroblast subpopulations in the BM were analyzed by FACS preselected by CD71 and Ter119 and according to their CD44 positivity and forward scatter (FSC) intensity as described by Chen et al.^33^. *Ctnnb1*^*OE-SEC*^ mice displayed increased numbers of all types of erythroblasts ranging from basophilic erythroblasts (PII) to orthochromatic erythroblasts and immature reticulocytes (PIV). On the contrary, the numbers of mature erythroid cells (PV) were significantly reduced suggesting a block in erythroid differentiation between PIV and PV (Fig. 3c, d). The same analysis of the spleen and liver for erythroid precursors revealed increased numbers of PII–PV in the liver and PII–PIV in the spleen, while PV were significantly reduced in the spleen (Supplementary Fig. 5f, g).

Transcriptomics of isolated polychromatic erythroblasts (PIII), as well as of orthochromatic erythroblasts and immature reticulocytes (PIV) of *Ctnnb1*^*OE-SEC*^ mice revealed significant regulation of 1687 (PIII) and 1962 (PIV) genes, respectively (Supplementary Data Files 1 and 2). Gene Set Enrichment Analysis (GSEA) revealed numerous dysregulated gene sets in PIII and PIV cells *Ctnnb1*^*OE-SEC*^ mice (Fig. 3e, f). Remarkably, GSEA in PIV revealed a striking loss of cell cycle and cell division associated pathways possibly indicating dysfunctional orthochromatic enucleation, as this process somewhat resembles cell division in an asymmetric manner (Fig. 3f). On the contrary, the Wnt signaling pathway was unaltered in both PIII and PIV erythroid precursor populations confirming absent leakiness of the *Stab2-iCreF3* driver mouse line toward HSPCs as well as erythroid precursor cells (Fig. 3g).

In order to exclude cell-intrinsic effects in *Ctnnb1*^*OE-SEC*^ stem and progenitor cells to cause fatal anemia, we performed transplantation experiments with HSC derived from *Ctnnb1*^*OE-SEC*^ donor or control *Ctnnb1*^*WT*^ donor mice (CD45.2) transplanted into lethally irradiated wildtype (CD45.1) recipients. *Ctnnb1*^*OE-SEC*^ or *Ctnnb1*^*WT*^ donor BM recipients did not show differences in survival until final examination at 6 months after transplantation (Fig. 3h). *Ctnnb1*^*OE-SEC*^ donor BM recipients did not develop fatal anemia as analyzed 1–6 months after transplantation (Fig. 3i). FACS analysis of the BM of all recipient mice revealed no differences in numbers of HSC, ST-HSC, MPP, MEP, CMP, and GMP among the transplanted groups at final examination after 6 months (Fig. 3j). Furthermore, analysis of donor-derived HSC and MEP in BMs of CD45.1 C57BL/6J recipients transplanted with CD45.2 HSC from *Ctnnb1*^*OE-SEC*^ and *Ctnnb1*^*WT*^ mice showed no differences in their contributions at 6 months after transplantation (Fig. 3k). Together these results argue against a cell-intrinsic defect in *Ctnnb1*^*OE-SEC*^ HSPCs to cause anemia and suggest that changes in the BM vascular niche indeed cause the block in terminal erythroid differentiation.

### Aberrant sinusoidal differentiation in *Ctnnb1*^*OE-SEC*^ mice

**Fig. 4:**
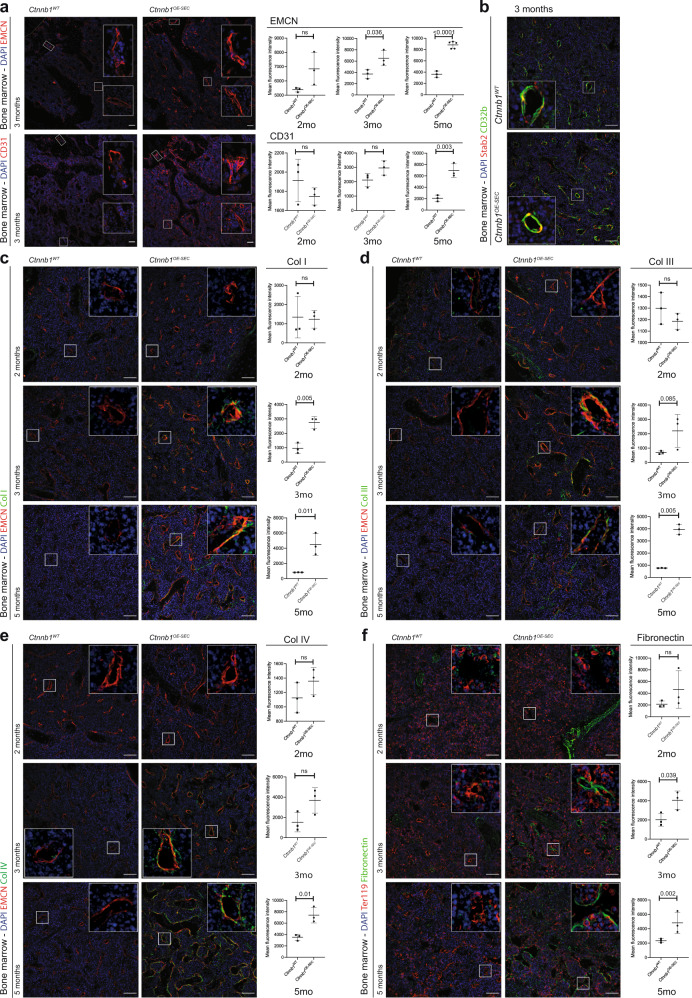
BM-SEC in *Ctnnb1*^*OE-SEC*^ mice exhibit aberrant sinusoidal differentiation including increased extracellular matrix (ECM) deposition. **a**–**f** IF staining for endothelial, erythroid and ECM markers of *Ctnnb1*^*WT*^ and *Ctnnb1*^*OE-SEC*^ bone marrow sections of 2-, 3-, or 5-month-old female mice. Co-IF of DAPI, **a** CD31 and EMCN (*n* = 3); **b** Stab2, CD32b (*n* = 3); **c** EMCN, Collagen I (Col I) (*n* = 3); **d** EMCN, Collagen III (Col III) (*n* = 3); **e** EMCN, Collagen IV (Col IV) (*n* = 3); and **f** Ter119, Fibronectin (*n* = 3). Scale bars: 50 μm. Quantifications of IFs are shown next to corresponding IF staining (**a**, **c**–**f**). The dots in the graphs represent individual mice. Three representative images have been used for quantification per individual mouse. Mean ± s.e.m. is shown for each group of mice in all graphs. Statistical significance was determined using Student’s *t*-test or Mann–Whitney *U* test (two-sided). ns, not significant. Source data, and employed statistical tests for **a**, **c**, **d**, **e**, **f** are provided as a Source Data file.

To understand the functional role of β-catenin overactivation in BM-SEC regarding ineffective erythropoiesis and fatal anemia, we analyzed vascular differentiation of BM-SEC in *Ctnnb1*^*OE-SEC*^ mice. The BM microvasculature of control mice showed a gradual decrease in endothelial expression of CD31 and EMCN from the epiphysis to the diaphysis resembling the previously described population of CD31^lo^EMCN^lo^ L-type EC that grossly correspond to BM-SEC^5,11^. On the contrary, the BM microvasculature of *Ctnnb1*^*OE-SEC*^ mice revealed an increased expression of both CD31 and EMCN throughout the BM sinusoidal vascular network along the epiphysis–diaphysis axis with preserved expression of other SEC-associated marker proteins such as Stab2 and CD32b^10^ (Fig. 4a, b and Supplementary Fig. 7b). Notably, a strong correlation was found between tissue EMCN expression upon immunofluorescent analysis of bone sections and the hemoglobin levels (Supplementary Fig. 6a). As SEC lack a formal continuous basement membrane or show reduced extracellular matrix (ECM) coverage as compared to other blood vascular and especially capillary EC (CEC), we performed an immunofluorescent analysis of collagens I, III, and IV, as well as of fibronectin in the BM of *Ctnnb1*^*OE-SEC*^ mice. Parallel to the exacerbation of anemia, increased deposition of these ECM molecules surrounding EMCN^+^ BM-EC became evident in 3-month-old and even more so in 5-month-old *Ctnnb1*^*OE-SEC*^ mice as compared to controls (Fig. 4c–f). Among these ECM molecules, overexpression of basement membrane-associated collagen IV was especially marked in *Ctnnb1*^*OE-SEC*^ mice (Fig. 4e). Similar angiocrine overexpression of ECM molecules was observed in the splenic sinusoids of *Ctnnb1*^*OE-SEC*^ mice (Supplementary Fig. 6b), whereas the liver vasculature did not show any differences in perivascular collagen expression (Supplementary Fig. 6c).

To further delineate the β-catenin-induced alterations in BM-SEC, we performed gene expression profiling of BM-EC isolated ex vivo from *Ctnnb1*^*OE-SEC*^ mice. KEGG pathway analysis showed significant activation or suppression of several important pathways, most notably activation of Wnt signaling and MYC targets and suppression of angiogenesis (Supplementary Fig. 6d). Activation of Wnt signaling indicated the functionality of β-catenin overactivation in *Ctnnb1*^*OE-SEC*^ mice. Microarray data were further corroborated by quantitative reverse-transcription PCR (qRT-PCR) analysis demonstrating significant upregulation of Wnt target genes *Axin2* and *Apccd1*, while *Lef1* (*p* = 0.055) or *TCF7* (*p* = 0.066) showed a tendency toward higher expression levels (Supplementary Fig. 6e). In addition, we performed qRT-PCR analysis on a predefined panel of marker genes for BM-SEC, continuous CEC and AEC^10^. These analyses showed significant downregulation of BM-SEC-associated genes such as *Rgs4*, *Angptl4*, *Gpr182*, and *Vegfr3* while AEC- or CEC-associated genes such as *CD34* or *Sparcl1* were significantly upregulated, respectively, demonstrating aberrant differentiation of BM-SEC in *Ctnnb1*^*OE-SEC*^ mice (Supplementary Fig. 6f). Notably, SEC-associated genes *Stab2* and *Vegfr3* were counter-regulated on the mRNA and protein levels (Supplementary Figs. 6f and 7a, b).

To gain more insight into the molecules potentially involved in BM-SEC-associated impairment of erythroid differentiation and maturation, we analyzed a predefined panel of angiocrine factors^31^ using the microarray data of BM-EC isolated ex vivo from *Ctnnb1*^*OE-SEC*^ mice. The five most strongly expressed genes from the resultant list of genes were further analyzed by qRT-PCR. Two of these genes, *Dickkopf (DKK)2* and *Fibroblast growth factor (FGF)23* were significantly upregulated (Supplementary Fig. 7c, d). Notably, *DKK2* is known to be induced in EC by basement membrane component laminin and to induce formation of well-formed, tight, pericyte-covered neo-vessels resembling continuous capillary structures^34^ indicating a role for *DKK2* in aberrant endothelial differentiation of BM-SEC in *Ctnnb1*^*OE-SEC*^ mice.

**Fig. 5:**
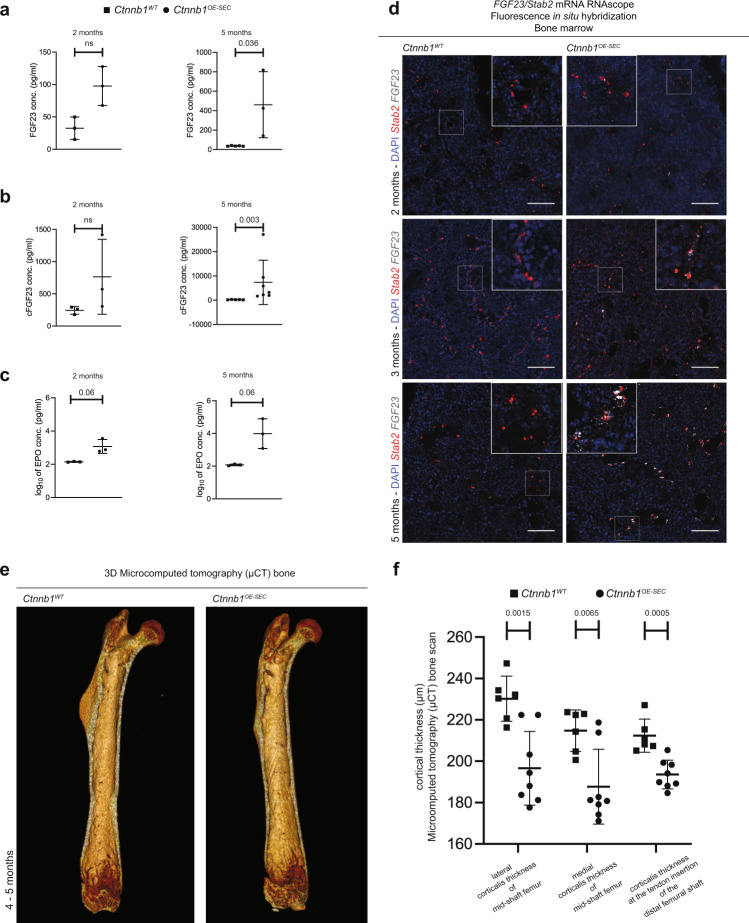
*Ctnnb1*^*OE-SEC*^ mice show de novo expression of FGF23 in BM-SECs and serum. **a** Serum FGF23 (2-month-old mice, *n* = 3; 5-month-old mice, *n* ≥ 3), **b** cFGF23 (2-month-old mice, *n* = 3; 5-month-old mice, *n* ≥ 5), and **c** EPO levels (2- and 5-month-old mice, *n* = 3) of *Ctnnb1*^*WT*^ and *Ctnnb1*^*OE-SEC*^ mice measured by ELISA. The dots in the graphs represent individual female mice. **d** mRNA RNAScope® FISH assay with DAPI, *FGF23* (white) and *Stab2* (red) of *Ctnnb1*^*WT*^ and *Ctnnb1*^*OE-SEC*^ bone marrow (2-, 3-, and 5-month-old male mice, *n* ≥ 3). Scale bars: 50 μm. **e**, **f** Micro-computed tomography (μCT) bone scan of femurs (right and left limb) of 4- and 5-month-old female *Ctnnb1*^*WT*^ (*n* = 6 from three mice) and *Ctnnb1*^*OE-SEC*^ (*n* = 8 from four mice). **e** Exemplary view of the inside from a 3D reconstructed μCT scan of *Ctnnb1*^*WT*^ and *Ctnnb1*^*OE-SEC*^ femurs. **f** μCT quantification of corticalis thickness (μm) at selected femoral regions. The dots in the graphs represent measurements of the lower extremities (right and left limb) of individual mice. Mean ± s.e.m. is shown for each group of mice in all graphs. Statistical significance was determined using Student’s *t*-test or Mann–Whitney *U* test (two-sided). ns, not significant. Source data, precise values of *n* and employed statistical tests for **a**, **b**, **c**, **f** are provided as a Source Data file.

As FGF23 is known to cause impaired bone mineralization in MDS and chronic kidney disease, and as it also acts as a negative regulator of erythropoiesis in vivo^35,36^, we analyzed FGF23 expression in *Ctnnb1*^*OE-SEC*^ mice in more detail. Notably, serum levels of full-length FGF23 and of C-terminal FGF23 (cFGF23) were significantly increased in 5-month-old *Ctnnb1*^*OE-SEC*^ mice (Fig. 5a, b), while erythropoietin (EPO) serum levels only showed a tendency toward higher values in *Ctnnb1*^*OE-SEC*^ serum (Fig. 5c). Analysis of BM tissue by fluorescence in situ hybridization (FISH) revealed upregulation of *FGF23* expression restricted to Stab2^+^ BM-SEC in increasing intensity from 2- to 5-month-old *Ctnnb1*^*OE-SEC*^ mice as compared to controls (Fig. 5d). To determine whether impairment of erythroid differentiation and maturation in *Ctnnb1*^*OE-SEC*^ mice was caused directly by FGF23, we used the human immortalized adult erythroid cell line BEL-A, which fully recapitulates normal erythropoiesis including enucleation^37^. In this human in vitro system, however, no direct effects of FGF23 on erythroid differentiation, maturation, and enucleation as well as apoptosis or proliferation were observed (Supplementary Fig. 7e). Moreover, the coreceptor *Klotho* required for FGF receptor activation did not reveal altered expressions neither in BM-SEC, nor in total BM, PIII, and PIV subpopulations (Supplementary Fig. 7f). On the contrary, when analyzing whether β-catenin overactivation of BM-SEC affected bone morphogenesis, we found that bone thickness was significantly reduced on μCT scans of femurs from *Ctnnb1*^*OE-SEC*^ mice while no structural abnormalities were detected (Fig. 5e, f).

## Discussion

Here, we discovered that constitutive β-catenin activation specifically in BM-SEC caused aberrant sinusoidal endothelial differentiation with altered EC marker gene expression and increased periendothelial ECM deposition as well as angiocrine dysregulation with de novo expression of *FGF23* and *DKK2*. Aberrant sinusoidal differentiation and angiocrine dysregulation entailed lethal anemia due to a block in terminal erythroid differentiation resulting in defective reticulocyte maturation. As a consequence, extramedullary hematopoiesis developed in the spleen and liver; however, the capacity did not suffice to compensate for the severe loss of red blood cells. The hematological phenotype observed in our study bears some resemblance to the phenotype of the Apc haploinsufficiency-mediated MDS-like mouse model in which activation of canonical Wnt signaling in cells of the BMME was shown to induce MDS-like disease without cell-intrinsic alterations in hematopoietic cells^20^. As the *Mx1-cre* driver line used in the Apc haploinsufficiency MDS-like model rather seem to target MSC than BM-SEC^22^, MSC rather than EC likely mediate these effects in this model. In support of this notion, the Apc haploinsufficiency MDS-like phenotype differs from the phenotype of the *Ctnnb1*^*OE-SEC*^ mice described here as it shows alterations in a different range of HSPC such as elevation of LSK, LT-HSC, and ST- HSC while MPP, MEP, BFU-E, and CFU-E were normal^38^. In *Ctnnb1*^*OE-SEC*^ mice LSK, LT-HSC, ST-HSC, and MPP were normal, while MEP, BFU-E, and CFU-E were significantly elevated. Furthermore, in the Apc haploinsufficiency model the roadblock in erythroid differentiation seemed to occur somewhat earlier at the R2/PII stage whereas reticulocyte maturation—severely affected in our *Ctnnb1*^*OE-SEC*^ mice—seemed normal^20,38^. Therefore, both models provide evidence that alterations of the Wnt signaling pathway in non-hemopoietic cells of the BMME can induce alterations also seen in MDS. Altogether, Wnt signaling in BM-SEC as demonstrated here seems to affect terminal erythroid differentiation impacting reticulocyte maturation. On the contrary, Wnt signaling in MSC affects erythropoiesis and hematopoiesis; furthermore, Wnt signaling in osteoblasts induces overt acute myeloid leukemia underscoring the important role of Wnt signaling in the development of hematological neoplasia. Notably, transplantation of HSC derived from a MDS mouse model into a healthy BM niche was shown to improve and delay the onset of MDS further supporting the notion that niche cells are intricately involved in initiation or progression of MDS^39^. Overall, it appears likely that altered Wnt signaling in different stromal cells of the BMME is involved in the diverse clinical variants of human MDS.

We show here that BM-SEC actively control erythropoiesis. β-catenin overactivation in BM-SEC induced a shift in vascular differentiation from EMCN^lo^CD31^lo^ L-type vessels to EMCN^hi^CD31^hi^ aberrant sinusoidal vessels with loss of (BM)-SEC genes, induction of continuous EC and AEC genes, and increased deposition of perisinusoidal Collagen I, III, IV, and Fibronectin^40^. Whereas H-type vessels have been shown to be less permeable for cells and solutes as compared to L-type vessels and are surrounded by pericytes^4,41^, electron microscopy studies demonstrated that egress of BM cells into the bloodstream occurs particularly at sites where pericytes are absent^42^, such as in L-Type BM-SEC. Furthermore, a lower number of tight junctions in BM-EC might facilitate egress of BM cells into the bloodstream via interdigital sliding of EC junctions^43^. Structural and molecular changes in BM-SEC of *Ctnnb1*^*OE-SEC*^ mice and subsequently in the BMME may contribute to the development of lethal anemia either by inhibiting terminal erythroid differentiation and/or by influencing the process of reticulocyte egress and maturation. We found numerous pathological, square-edged, polygonal reticulocytes within the BM stroma close to the abluminal surface of the BM sinuses and we detected immature reticulocytes with nuclear remnants in the circulation. Therefore, we propose that reticulocytes are released into the circulation in *Ctnnb1*^*OE-SEC*^ mice before they have matured to the stage when they are normally released. These pathological reticulocytes obviously cannot accomplish definitive maturation into red blood cells, and thus may be prone to be easily destroyed. Furthermore, hematopoietic cells other than erythrocytes also managed to egress in *Ctnnb1*^*OE-SEC*^ mice. Therefore, mechanisms other than impaired egress had to be considered to cause fatal anemia in *Ctnnb1*^*OE-SEC*^ mice such as de novo secretion of angiocrine inhibitors or loss of angiocrine inducers of terminal erythroid differentiation and reticulocyte maturation.

Notably, we found increased levels of FGF23 and cFGF23 in the serum of *Ctnnb1*^*OE-SEC*^ mice. FGF23 was previously reported to be elevated in serum of MDS patients and cFGF23 correlated negatively with their hemoglobin levels^36^. While the expression of FGF23 is detected in many different tissues during development, the bone is recognized as the main source of circulating FGF23 during adulthood under normal health conditions^44^. Confirming our findings, upregulation of BM endothelial FGF23 as well as continuous EC markers Pecam1 and Apln was demonstrated after irradiation in H-type and E-type capillary vessels, but not BM-SEC^9,12,13,45^. Hitherto, there is no evidence for a direct link between FGF23 expression and Wnt-/β-catenin signaling in BM-SEC. In the heart, however, FGF23 was shown to activate β-catenin and to induce collagen and fibronectin expression in fibroblasts as was also seen in *Ctnnb1*^*OE-SEC*^ mice^46,47^. Bone-derived FGF23 negatively regulates erythropoiesis in a paracrine manner and the FGF23 receptors and its coreceptor Klotho are expressed in erythroid precursor cells in a murine MDS model^35,36,48^. In the human erythroid cell line BEL-A in vitro, no direct effects of FGF23 on erythroblasts were detected when analyzing erythroid expansion, viability, apoptosis, and enucleation. Nevertheless, FGF23 may well contribute to the complex pathogenesis of lethal anemia in *Ctnnb1*^*OE-SEC*^ mice in vivo in a direct or indirect manner or in concert with additional features instructed by aberrantly differentiated BM-SEC such as membrane charge, adhesion molecules, or other angiocrine factors. Therefore, FGF23 should be further investigated as a promising target using FGF23-deficient mice or anti-FGF23 antibodies in *Ctnnb1*^*OE-SEC*^ mice and in murine and human MDS. In addition to FGF23, DKK2 was identified as an angiokine induced de novo in transdifferentiated BM-SEC in *Ctnnb1*^*OE-SEC*^ mice. DKK2 is known to induce angiogenesis and to promote vascular stabilization and pericyte coverage during vessel maturation. Thus, we hypothesize that DKK2 may be involved in causing aberrant differentiation of BM-SEC to a tighter type of endothelium in an autocrine manner.

Altogether, our findings contribute to a better understanding of the regulatory function of heterogeneous cell populations within the BMME regarding hematopoiesis. Our study demonstrates that cell-specific alterations of BM-SEC can cause refractory anemia as in MDS-like disease. The BMME has recently been shown to comprise at least 17 different cell populations as analyzed by single-cell RNA-Seq technology^14,49^. Here, we provide a mouse model to specifically study BM-SEC in vivo in this context and confirm BM-SEC as a significant BMME cell population controlling terminal erythropoiesis and reticulocyte maturation. Furthermore, Wnt signaling seems to be an important pathway in determining BM-SEC identity and functions as has similarly been shown for several EC subpopulations in other organs^50–54^. The Wnt signaling pathway and its downstream angiocrine target molecules such as FGF23 and DKK2 may also bear therapeutic potential in various types of anemia or in hematopoietic neoplasms. Therefore, BM-SEC need to be considered as main actors during normal functions and pathogenic alterations in the BMME.

## Methods

### Animals

All animals were housed under specific pathogen-free conditions in an animal facility (Heidelberg University) at a 12 h/12 h day/night cycle in individually ventilated plastic cages (Green Line, Tecniplast, Buguggiate, Italy) with adjusted air temperature (21 °C) and 50% relative humidity. All mice were fed ad libitum with a standard rodent diet (V1534-000, Ssniff, Soest, Germany) and free access to water. The authors have complied with all relevant ethical regulations for animal testing and research. Namely, all animal experiments were performed in accordance with Federal Animal Regulations and were institutionally approved by the district government Karlsruhe and performed under institutional guidelines. To generate a sinusoidal endothelial subtype-specific Ctnnb1 gain-of-function model (*Ctnnb1*^*OE-SEC*^) *Stab2-iCreF3*^*tg/wt*^ (Tg(Stab2-icre)1.3Cger)^25^ were crossed with *Ctnnb1(ex3)*^*fl/wt*^ (*Ctnnb1*^*tm1Mmt*^)^29^ mice. Female and male animals aged 2–5 months were used in this study. Here Cre-mediated removal of exon 3 coded amino acids results in a dominant stable form of Ctnnb1 protein and subsequent activation of β-catenin pathway in target cells^29^. Cre-negative *Ctnnb1(ex3)*^*fl/wt*^ siblings served as negative controls in experimental groups. Sinusoidal endothelial specificity of Cre activity was analyzed in crosses of *Stab2-iCreF3*^*tg/wt*^ transgenic mice with *R26YFP*^*fl/fl*^ (B6.129×1-Gt(ROSA)26Sortm1(EYFP)Cos/J) [JAX 006148]^55^ reporter animals. Female and male reporter animals were analyzed at the age of embryonic day (E)17.5 and older.

### Organ dissection, cryopreservation, and paraffin embedding

Mice were sacrificed by cervical dislocation. Liver, spleen, lymph node, lung, kidney, heart, and intestine tissue samples were either embedded in OCT compound (Sakura) and frozen in liquid nitrogen or fixed in 4% paraformaldehyde at 4 °C, followed by paraffin embedding according to routine protocols. Bones were fixed in 4% paraformaldehyde at 4 °C for 4 h and decalcified in 0.5 M EDTA at pH 7.4 for 24 h. Decalcified bones were immersed in 20% sucrose and 2% polyvinylpyrrolidone (PVP) in PBS for 24 h and finally embedded in OCT or 8% porcine gelatin, 20% Sucrose, and 2% PVP in PBS^56^. For embryo preparation, pregnant mice were sacrificed by cervical dislocation. Embryos were dissected and the yolk sac was collected for genotyping. For GFP/YFP staining embryos were embedded in OCT after incubation overnight in 4% PFA followed by 24 h 30% sucrose treatment.

### Histology, in situ hybridization, and immunofluorescence

For blood smear samples, 5 μL of whole blood sample mixed with up to 2.0 mg EDTA/mL was smeared uniformly across a microscopic slide using another slide at a 30-degree smearing angle and air-dried for 5 min. For differential cytomorphological assessment of BM, whole BM was spread onto glass slides and fixed using 100% methanol. Air-dried blood films and BM slides were stained with May-Grünwald/Giemsa stain and brilliant cresyl blue solutions according to standard protocols provided by the manufacturer. Paraffin-embedded tissues were sectioned in 3 μm. For hematoxylin & eosin, periodic acid-Schiff, and Prussian blue staining, formalin-fixed, paraffin-embedded samples were processed according to standard protocols provided by the manufacturer.

RNA FISH was conducted using RNAscope Multiplex FISH version 2 assay (323110; Advanced Cell Diagnostics) kits with mouse-specific probes against the positive control mouse Ppib (Cyclophilin B) gene, mouse Stab2-Channel 2 (4249 – 5075 NM_138673.2) and mouse FGF23-Channel 1 (2 – 1131 NM_022657.4) according to the manufacturer’s protocols.

For immunofluorescence (IF), cryosections (8–14 μm) were air-dried, rehydrated in PBS, and blocked in 5% donkey serum in PBS for 30 min. Primary antibody was incubated for 2 h at room temperature or overnight at 4 °C. Sections were washed three times in PBS before incubation of appropriate Alexa-Fluor coupled secondary antibodies for 1 h at room temperature. Nuclei were counterstained with DAPI (D1306, Thermo Fisher Scientific) or Toto3 iodide (T3604, Life Technologies). Finally, sections were thoroughly washed in PBS before mounting with Dako fluorescent mounting medium (Dako).

Sections were visualized with Axioskop 2 (Zeiss), ECLIPSE Ci microscope (Nikon), ECLIPSE Ni-E microscope (Nikon), or DMi8 (Leica). Processing of images was performed by NIS-Elements Imaging Software AR 5.02.00 (Nikon) and ImageJ 1.52e software (https://imagej.nih.gov/ij/). For quantification of IF images, RGB images were split into separate channels corresponding to stainings (including DAPI) in ImageJ. Images of a staining were uniformly visually adjusted using the “Multiply” and “Subtract Background” commands in ImageJ. Then, an automated threshold using the “Otsu” command was applied and the mean pixel signal intensity of the remaining area was analyzed.

### Hematology and flow cytometry analysis (FACS)

Hematological blood counts were done from EDTA whole blood samples with automated blood analyzers (Sysmex XN-9000) using protocols accredited according to norm ISO 15189 (German Accreditation Body DAkkS). BM cells were released from the bones by flushing murine tibia and fibula bones with FACS buffer (5% FCS in PBS), and cells were harvested from the flow-through of a 70-μm filter (BD Falcon). Spleens were dissociated directly by passing the cells through a 40-μm filter. Livers were perfused via the portal vein with ice cold PBS, dissected, mechanically minced, digested at 38 °C in collagenase/GBSS (G9779, Sigma-Aldrich) and filtered through a 250-μm filter (BD Falcon). Then, 30 µL of peripheral blood was collected using EDTA-containing microtubes (Sarstedt), then suspended in PBS; the erythrocytes cells were lysed with RBC Lysis Buffer (10X, BD Pharm Lyse) by incubating them for 15 min at room temperature. Cell suspensions (BM, spleen, liver, peripheral blood) were incubated for 30 min with 500 μg/mL whole mouse IgG (Jackson ImmunoResearch Laboratories) for blocking Fc receptors and stained for 30 min with titrated concentrations of fluorescent dye-labeled antibodies in FACS buffer (5% FCS in PBS) on ice. Dead cells were stained with either Sytox Blue (Invitrogen) or Fixable viability stain 780 (BD Horizon). Flow cytometry was performed using a BD Biosciences FACSCanto II flow cytometer, a BD Biosciences LSR Fortessa cell analyzer or sorted by BD Bioscience FACSAriaIII. All data were analyzed using FlowJo 10.7.1 (Tree Star Inc.) or FACS Diva software (BD Bioscience). Reticulocytes and immature reticulocyte fraction were measured with a Sysmex XN-9000 analyzer according to the manufacturer’s protocols.

### Antibodies

Primary antibodies: monoclonal rat anti-Endomucin (Clone V.7C7, Cat. No. 14-5851-82, eBioscience; dilution 1:100), polyclonal chicken anti-GFP/YFP (Cat. No. ab13970, abcam; dilution 1:800), polyclonal goat anti-CD32 (Cat. No. AF1460, R&D Systems; dilution 1:75), polyclonal rabbit anti-alpha smooth muscle Actin (Cat. no. ab5694, abcam; dilution 1:500), monoclonal rat anti-VEGFR-3 (Cat. No. 552857, BD PharMingen; dilution 1:100), polyclonal rabbit anti-Cav1 (Cat. No. sc-7305, Santa Cruz; dilution 1:250), monoclonal rat anti-CD45 (Clone 30-F11, Cat. No. 550539, BD PharMingen; dilution 1:400), monoclonal rat anti-Ter119 (Clone TER-119, Cat. No. 550565, BD PharMingen; dilution 1:200), polyclonal goat anti-c-kit-antibody (Cat. No. AF1356, R&D Systems; dilution 1:200), monoclonal rat anti-CD31 (Clone MEC13.3, Cat. No. 102502, BioLegend; dilution 1:300), polyclonal rabbit anti-Collagen type I (Cat. No. R1038, Acris; dilution 1:200), polyclonal rabbit anti-Collagen type III alpha 1 chain (Cat. No. R1040, Acris; dilution 1:200), polyclonal rabbit anti-Collagen IV (Cat. No. GTX19808, Genetex; dilution 1:200), polyclonal rabbit anti-fibronectin (Cat. No. ab23750, Abcam; dilution 1:100), polyclonal goat anti-Podocalyxin (Cat. No. AF1556, R&D Systems; dilution 1:100), polyclonal rabbit anti-Glutamine Synthetase (Cat. No. G2781, Sigma-Aldrich; dilution 1:2000), polyclonal goat anti-Arginase I (Cat. No. sc-18351, Santa Cruz; dilution 1:50), polyclonal goat anti-Lyve1 (Cat. No. AF2125, R&D Systems; dilution 1:400), mouse anti–Stabilin-2 clone 3.1 antibody (dilution 1:700)^28^. The following secondary antibodies were all purchased from Dianova and diluted 1:400: Alexa-Fluor 488 (donkey-anti-chicken (Cat. No. 703-545-155), donkey-anti-goat (Cat. No. 705-545-147), donkey-anti-rabbit (Cat. No. 711-545-152), donkey-anti-rat (Cat. No. 712-545-153), Alexa-Fluor 647 (donkey-anti-chicken (Cat. No. 703-605-155), donkey-anti-goat (Cat. No. 705-605-147), donkey-anti-rabbit (Cat. No. 711-605-152), donkey-anti-rat (Cat. No. 712-605-153), and Cy3 (donkey-anti-chicken (Cat. No. 703-165-155), donkey-anti-goat (Cat. No. 705-165-147), donkey-anti-rabbit (Cat. No. 711-165-152), donkey-anti-rat (Cat. No. 712-165-153). For FACS staining the following antibodies were used: monoclonal rat anti-CD3ε–APC–eFluor 780 (Clone 17A2, Cat. No. 47-0032-82, eBioscience; dilution 1:25), monoclonal rat anti-CD3ε–PE (Clone 145-2C11, Cat. No. 553063, BD PharMingen; dilution 1:25), monoclonal rat anti-CD3ε–BV510 (Clone 17A2, Cat. No. 100233, BioLegend; dilution 1:100), monoclonal rat anti-CD16/32–PE–Cy7 (Clone 93, Cat. No. 25-0161-82, eBioscience; dilution 1:400), monoclonal rat anti-CD34–eFluor660 (Clone RAM34, Cat. No. 50-0341, eBioscience; dilution 1:400), monoclonal rat anti-CD48-AF700 (Clone HM48-1, Cat. No. 103426, BioLegend; dilution 1:400), monoclonal rat anti-Sca1–PerCP–Cy5.5 (Clone D7, Cat. No. 45-5981, eBioscience; dilution 1:400), monoclonal rat anti-CD19–BV421 (Clone 6D5, Cat. No. 50-0341, eBioscience; dilution 1:400), monoclonal rat anti-CD19–BV605 (Clone 6D5, Cat. No. 115540, BioLegend; dilution 1:800), monoclonal rat anti-CD19–PerCP–Cy5.5 (Clone 6D5, Cat. No. 115534, BioLegend; dilution 1:100), monoclonal rat anti-CD117–BV711 (Clone 2B8, Cat. No. 105835, BioLegend; dilution 1:800), monoclonal rat anti-CD150–BV605 (Clone TC15–12F12.2, Cat. No. 115927, BioLegend; dilution 1:100), monoclonal rat anti-Gr1-APC (Clone RB6-8C5, Cat. No. 553129, BD PharMingen; dilution 1:400), monoclonal rat anti-Gr1-PE (Clone RB6-8C5, Cat. No. 553128, BD PharMingen; dilution 1:400), monoclonal rat anti-CD45-PE-Cy7 (Clone 30-F11, Cat. No. 25-0451-82, eBioscience; dilution 1:400), monoclonal rat anti-CD45-APC (Clone 30-F11, Cat. No. 559864, BD PharMingen; dilution 1:50), monoclonal rat anti-CD11b–PerCP–Cy5.5 (Clone M1/70, Cat. No. 45-0112-82, eBioscience; dilution 1:400), monoclonal rat anti-CD11b-PE (Clone M1/70, Cat. No. 12-0112-82, eBioscience; dilution 1:800), monoclonal rat anti-CD44–BV605 (Clone IM7, Cat. No. 103047, BioLegend; dilution 1:400), monoclonal rat anti-Ter119–PE (Clone Ter119, Cat. No. 553673, BD PharMingen; dilution 1:50), monoclonal rat anti-Ter119–APC-Cy7 (Clone Ter119, Cat. No. 116223, BioLegend; dilution 1:100), monoclonal rat anti-CD71–PE-Cy7 (Clone RI7217, Cat. No. 113811, BioLegend; dilution 1:100), monoclonal rat anti-CD71–PE (Clone RI7217, Cat. No. 113808, BioLegend; dilution 1:100), monoclonal rat anti-CD45.1-BV785 (Clone A20, Cat. No. 110743, BioLegend; dilution 1:100), monoclonal rat anti-CD45.2-BV711 (Clone 104, Cat. No. 563685, eBioscience; dilution 1:400), monoclonal rat anti-CD45R-BV421 (Clone RA3-6B2, Cat. No. 103251, BioLegend; 1:100), monoclonal rat anti-IgM-FITC (Clone 121-15F9, Cat. No. 11-5890-85, eBioscience; dilution 1:100), monoclonal rat anti-MHCII-APC (Clone M5/114.15.2, Cat. No. 17-5321-81, eBioscience; dilution 1:400), monoclonal rat anti-Ly6C-APC-Cy7 (Clone AL-21, Cat. No. 560596, BD PharMingen; dilution 1:100), monoclonal rat anti-Ly6G–PerCP–Cy5.5 (Clone 1A8, Cat. No. 560602, BD PharMingen; dilution 1:100), monoclonal rat anti-CD115-BV605 (Clone AFS98, Cat. No. 135517, BioLegend; dilution 1:400), monoclonal rat anti-EMCN-PE (Clone V.7C7, Cat. No. 12-5851-82, eBioscience).

The lineage cocktail was composed of monoclonal rat anti-CD3ε-BV421 (Clone 17A2, Cat. No. 100228, BioLegend; dilution 1:200), monoclonal rat anti-CD4-BV421 (Clone GK1.5, Cat. No. 100443, BioLegend; dilution 1:800), monoclonal rat anti-CD8-BV421 (Clone 53-6.7, Cat. No. 100753, BioLegend; dilution 1:800), monoclonal rat anti-CD11b-BV421 (Clone M1/70, Cat. No. 101251, BioLegend; dilution 1:800), monoclonal rat anti-CD19-BV421 (Clone 6D5, Cat. No. 115549, BioLegend; dilution 1:400), monoclonal rat anti-Gr-1-BV421 (Clone RB6-8C5, Cat. No. 108445, BioLegend; dilution 1:800), and monoclonal rat anti-Ter119-BV421 (Clone Ter119, Cat. No. 116234, BioLegend; dilution 1:200). Sytox blue (Cat. No. S34857, Thermo Fisher Scientific; dilution 1:25000) and Fixable Viability Dye eFluor 780 (Cat. No. 65-0865-18, eBioscience; dilution 1:1000) were used for dead cell discrimination. All populations were gated for live single cells. The gating strategies are shown in Supplementary Fig. 8a–h. Stem cell populations were defined as: HSCs (Lin^-^Kit^+^Sca-1^+^CD150^+^CD48^-^), ST-HSCs (Lin^-^Kit^+^Sca-1^+^CD150^-^CD48^-^), MPPs (Lin^-^Kit^+^Sca-1^+^CD150^-^CD48^+^), CMPs (Lin^-^Kit^+^Sca-1^-^CD34^+^CD16/32^low^), GMPs (Lin^-^Kit^+^Sca-1^-^CD34^+^CD16/32^+^), MEPs (Lin^-^Kit^+^Sca-1^-^CD34^-^CD16/32^-^). Erythroid progenitors were defined as: PII (Basophilic erythroblasts: CD3^-^, CD19^-^, CD71^+^, Ter119^+^, CD44^+^, FSC^+^), PIII (Polychromatic erythroblasts: CD3^-^, CD19^-^, CD71^+^, Ter119^+^, CD44^med^, FSC^med^), PIV (Orthochromatic erythroblasts and reticulocytes: CD3^-^, CD19^-^, CD71^+^, Ter119^+^, CD44^low^, FSC^low^), PV (Erythrocytes: CD3^-^, CD19^-^, CD71^-^, Ter119^+^, CD44^-^, FSClow). EMCN^+^ cells were defined as CD11b^-^EMCN^+^. Immune cells were defined as: CD3^+^ (Size/CD3^+^CD19^-^), CD19^+^ (Size/CD3^-^CD19^+^), CD11b^+^Gr^-^ (Size/CD3^-^CD19^-^CD11b^+^Gr^-^), CD11b^+^Gr^+^ (Size/CD3^-^CD19^-^CD11b^+^Gr^+^), Pro-B Cells (CD43^mid^CD45R^+^), Pre-B Cells (CD43^-^CD45R^+^IgM^-^), Immature B Cells (CD43^-^CD45R^lo^IgM^+^), Mature B Cells (CD43^-^CD45R^hi^IgM^+^), Ly6G^+^ (Size/Ly6C^mid^Ly6G^+^), Ly6C^lo^CD115^+^ (Size/Ly6C^lo^Ly6G^-^CD115^+^), Ly6C^+^CD115^+^ (Size/Ly6C^+^Ly6G^-^CD115^+^).

### Microarray data and bioinformatic

RNA was extracted from FACS-sorted erythroblastic cells, PIII, PIV, and BM-EC using InnuPrep RNA Mini Kit (Analytik Jena). ECs were isolated from murine BM as previously described by Kusumbe et al.^6,9^. Briefly, murine long bones were crushed in Ca^2+^/Mg^2+^ free PBS, digested with 0.7 mg/mL Collagenase A (Cat. No. 10103586001, Sigma-Aldrich) for 45 min at 37 °C, filtered through a 40-μm mesh and incubated with 25 μL of EMCN-antibody (monoclonal rat anti-EMCN, clone V.7C7, Cat. No. sc-65495, Santra Cruz Biotechnology) coated dynabeads (Cat. No. 11035, Invitrogen) for 25 min at 4 °C under constant rotation. EMCN^+^ cells were then extracted by magnetic separation of dynabead-conjugated cells. Gene expression profiling was performed using arrays of mouse MoGene-2_0-st-type from Affymetrix. Biotinylated antisense complementary DNA (cDNA) was then prepared according to the standard labeling protocol with the GeneChip^®^ WT Plus Reagent Kit (from Thermo Fisher Scientific). Afterwards, the hybridization on the chip was performed on a GeneChip Hybridization oven 640, dyed in the GeneChip Fluidics Station 450 with the GeneChip^®^ Hybridization, Wash and Stain Kit and thereafter scanned with a GeneChip Scanner 3000. All of the equipment used was from the Affymetrix-Company.

A Custom CDF Version 22 with ENTREZ-based gene definitions was used to annotate the arrays^57^. The raw fluorescence intensity values were normalized applying quantile normalization and RMA background correction. One-way ANOVA was performed to identify differential expressed genes using a commercial software package SAS JMP15 Genomics, version 10, from SAS (SAS Institute). A false positive rate of *a* = 0.05 with FDR correction was taken as the level of significance.

GSEA was used to determine whether defined lists (or sets) of genes exhibit a statistically significant bias in their distribution within a ranked gene list using the *fgsea* package^58^ ran under R v3.4.0^59^. Pathways belonging to various cell functions such as cell cycle or apoptosis were obtained from public external databases (KEGG, http://www.genome.jp/kegg and molecular signature database, MSigDB, v6.2 hallmark gene set collection^60^). The gene set for angiocrine factors^31^ was defined merging published factors from Rafii et al.^61^ with Gene Ontology gene set cytokine activity filtered for murine genes^62,63^. Heatmaps were created with the ComplexHeatmap package^64^.

### Quantitative reverse-transcription PCR (qRT-PCR)

RNA was extracted from primary ECs using E.Z.N.A. TM Total-RNA-Kit I (OMEGA Biotec, Norcross, GA, USA). cDNA was synthesized with RevertAid H-Minus M-MuLV Reverse Transcriptase (Thermo Fisher Scientific). Quantitative PCR was performed on a qTOWER 3G touch thermal cycler (Analytik Jena) using innuMIX qPCR SyGreen Sensitive (845-AS-1310200, Analytik Jena). Primers were designed using Primer-BLAST (http://www.ncbi.nlm.nih.gov/tools/primer-blast/). All the primers used in the present study are listed in Supplementary Table 1. Normalized expression values were calculated using the Pfaffl method considering amplification efficiency values determined by standard curves^65^.

### Transplantation

For HSC sorting/enrichment, BM cells were stained with commercially prepared antibodies (see Supplementary Methods) and depleted with Dynabeads (Life technologies) according to the manufacturer’s instructions. In total, 1.2 × 10^3^ Lin^-^Kit^+^Sca-1^+^CD150^+^CD48^-^ cells from 10-week-old Ctnnb1^OE-SEC^ mice (CD45.2) were sorted (FACSAriaIII, Becton & Dickinson) and injected intravenously along with 1 × 10^5^ Lin^-^ BM cells sorted from CD45.1 mice into lethally irradiated (1100 cGy; split dose with 4 h time gap between each dose; Cesium 137GammaCell40 Irradiator, Best Theratronics) Pep Boy, B6 Cd45.1 recipients. Recipient mice were maintained on antibiotic water (Sulfamethoxazol/Trimethoprim, 400 mg/L) for 14 days. Blood samples were analyzed 4, 8, 12, 16, 20, and 24 weeks post transplantation for Hb-content and donor chimerism. BM of recipient mice was isolated and analyzed for donor-derived HSPC by flow cytometry.

### Colony-forming unit (CFU) assays

Singularized BM or spleen cell extracts were harvested in Iscove’s modified Dulbecco’s medium (Thermo Fisher Scientific) containing 2% FCS (Biochrom) plated in gridded 35-mm-diameter cell culture dishes (Sarstedt) at cell concentrations as suggested by the manufacturer, in triplicate. MethoCult™ GF M3434 (Stemcell Technologies) was used to stimulate mouse hematopoietic progenitor cells such as multi-potential granulocyte, erythroid, macrophage, megakaryocyte progenitor cells (CFU-GEMM), representing most colonies, but also BFU-E, granulocyte-macrophage progenitor cells (CFU-GM, CFU-G, and CFU-M). MethoCult™ M3334 was used for detection of CFU-E and MethoCult™ SF M3436 for BFU-E. Colonies were analyzed after 2 days (CFU-E), 10–12 days (CFU-GEMM, CFU-GM, CFU-G, CFU-M, and BFU-E), and 10–14 days (BFU-E) with an Axio Vert.A1 microscope (Zeiss).

### Transmission electron microscopy (TEM)

After perfusion-fixation with 2% formaldehyde, 2% glutaraldehyde, 1 mM MgCl2, 1 mM CaCl2, in 100 mM Na-phosphate buffer, pH 7.2 bones (femur) were excised and immersion-fixed for 24 h at 4 °C, decalcified in 100 mM EDTA at neutral pH for 3 days at 4 °C. Medium was changed once daily. The softened samples were cut into pieces using razorblades and washed in deionized water. Next, treatment for conventional epoxy-resin embedding and TEM consisted of post-fixation in 1% osmium tetroxide, en-bloc staining with 1% uranylacetate, graded dehydration with ethanol and embedding in epoxide (Glycidether, NMA, DDSA: Serva). Ultrathin sections at nominal thickness of 60 nm and contrast-stained with lead-citrate and uranylacetate were observed in Zeiss EM 910 at 100 kV (Zeiss) and micrographs taken with a slow scan CCD camera (TRS).

### In vitro assay

BEL-A cells^66,67^, established in the lab of Deborah E. Daniels and Jan Frayne, were cultured in StemSpan™ SFEM (Stemcell Technologies) containing 50 ng/mL SCF, 3 U/mL EPO, 1 μM dexamethasone, and 1 µg/mL doxycycline. To induce erythroid differentiation, expanding cells were transferred to primary medium supplemented with 1 µg/mL doxycycline for 4 days, and for a further 4 days without doxycycline. Cells were cultured with supplementation of either 100 ng/mL FGF23 (R&D Systems) or vehicle control for 48 h before sample collection for analysis. Cell viability was determined by trypan blue exclusion test. For FACS, aliquots of 2 × 10^5^ cells were incubated with band 3 primary antibody (BRIC71; IBGRL) in PBS containing 1% (w/v) BSA (Park Scientific Ltd) and 2 mg/mL glucose (PBS-AG), followed by APC-secondary antibody (BioLegend), or with conjugate antibodies (Annexin V-FITC or CD36-Vioblue; Miltenyi Biotech). Cells were analyzed on a BD LSR Fortessa flow cytometer. From day 6 of differentiation onwards cells were incubated with 5 μg/mL Hoechst 33342 nucleic acid stain (Merck) to distinguish the erythroblast and reticulocyte populations. Propidium iodide was used to exclude non-viable cells from analyses for band 3, CD36, and percentage reticulocyte measurements. Data were analyzed using FlowJo v10.6.1 (FlowJo LLC).

### Micro-computed tomography (CT) imaging

An industrial micro-CT (Y.Fox, Yxlon) equipped with a multifocus transmission x-ray tube and a 14-bit direct amorphous silicon flat panel detector (PaxScan 2520 D/CL; Varian Medical Systems, Inc.), in which the object is rotated around its horizontal axis in the cone beam, was used^68,69^. The source-object and object-detector distances were adjusted so that the sample filled the field of view. Imaging was performed using a tube voltage of 80 kV, tube current was set to 75 μA, and continuous image acquisition was carried out at 30 frames per second. A total of 990 projections were acquired per scan during a rotation of 360° within 33 s. Raw data were reconstructed with a filtered back projection algorithm with a matrix of 512 × 512 × 512 (Reconstruction Studio, Tera Recon) using a low pass filter (Shepp-Logan) to reduce image noise. The resulting digital imaging and communications in medicine data were analyzed using OsiriX imaging software (OsiriX v. 5.0.2, 64-bit; Pixmeo, SARL, Bernex).

### Blood parameters and ELISA

Relative hemoglobin values were analyzed based on photometric detection of cyanmethemoglobin. Plasma was collected was analyzed for the following routine parameters: LDH, K^+^ and Bilirubin (Roche cobas c 311 analyzer). Serum iron levels were measured with Dimension Vista^®^ 1500 analyzer (Siemens). FGF23, cFGF23, and EPO in plasma were detected by Mouse FGF23 DuoSet ELISA (DY2629-05, R&D Systems), Mouse/Rat FGF23 (C-Term) (60-6300, Quidel), and Mouse Erythropoietin/EPO Quantikine ELISA Kit (MEP00B, R&D Systems) according to the manufacturer’s instructions.

### Statistics and reproducibility

Statistical analyses were performed with GraphPad Prism 9.1.2 (GraphPad) and SigmaPlot 11 Software (Systat Software GmbH). For pairwise comparisons the two-tailed unpaired Student’s *t*-test and Mann–Whitney *U* test were used. Differences between data sets with *p* < 0.05 were considered statistically significant. Data are presented as means with error bars indicating standard error. All experiments have been performed with a minimum of three age- and sex-matched biological replicates. Micrographs shown in the figures are representative selections of at least three different micrographs per animal of a minimum of three independent biological samples.

### Reporting summary

Further information on research design is available in the Nature Research Reporting Summary linked to this article.

## Supplementary information

Supplementary Information

Reporting Summary

Description of Additional Supplementary Files

Supplementary Data 1

Supplementary Data 2

## Data Availability

The raw and normalized microarray data of isolated PIII, PIV, and BM endothelial cells generated in this study have been deposited in the Gene Expression Omnibus database under the accession number GSE180522 and made publicly available. The processed data of statistically significant differentially expressed genes of isolated erythroid PIII and PIV populations are provided in Supplementary Data Files 1 and 2. Source data are provided with this paper.

## Source data

Source Data
