# Supplementary material for: Bone marrow sinusoidal endothelium controls terminal erythroid differentiation and reticulocyte maturation
Source: Nat Commun. 2021 Nov 29;12:6963. doi: 10.1038/s41467-021-27161-3 (PMC8630019; doi:10.1038/s41467-021-27161-3)
Supplement: Supplementary file 1 — Supplementary Information [file 41467_2021_27161_MOESM1_ESM.pdf]

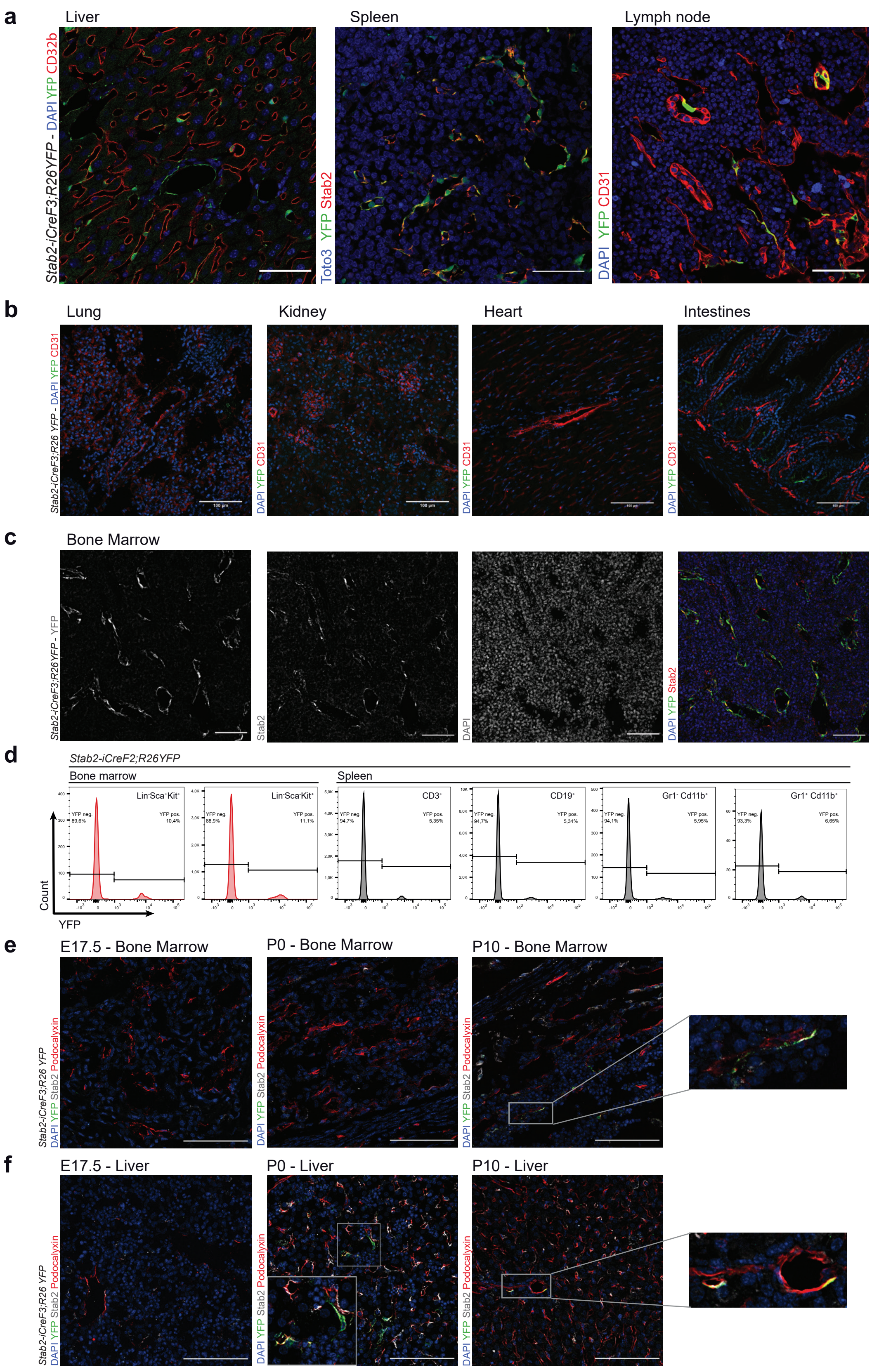

Supplementary Fig. 1 *Stab2-iCreF3;R26YFP* mice show reporter activity in sinusoidal endothelial cells of the liver, spleen and lymph node. (a-b) Immunofluorescence (IF) staining for endothelial markers of liver, spleen, lymph node, lung, kidney, heart and intestine of 6 months old *Stab2-iCreF3;R26YFP* mice. (a) Co-IF of YFP, DAPI and CD32b in the liver (n = 4); YFP, Toto3 and Stab2 in the spleen (n = 4); YFP, DAPI and CD31 in the lymph node (n = 3). Scale bars 50 µm. (b) Co-IF of YFP, DAPI and CD31 in the lung (n = 3); kidney (n = 3); heart (n = 3); intestine (n = 3). Scale bars 100 µm. **(c)** Co-IF of YFP, DAPI and Stab2 of Stab2-iCreF3;R26YFP bone marrow samples shown as single-channel and merged photomicrographs (n = 3). Scale bars 50 µm. **(d)** Quantification of YFP labeling frequencies of hematopoietic cells in BM ($Lin^-Sca\text{-}1^+Kit^+$ and $Lin^-Sca\text{-}1^-Kit^+$ cells per $3*10^6$ bone marrow cells; n = 3) and the spleen ($CD3^+$ T cells, $CD19^+$ B cells, $Gr1^-CD11b^+$ and $Gr1^+CD11b^+$ myeloid cells per $5*10^5$ spleen cells; n = 3) of adult *Stab2-iCreF2;R26YFP*[1] mice. **(e, f)** Co-IF of YFP, DAPI, Stab2 and Podocalyxin in (e) bone marrow and (f) liver tissues (n = 3) of *Stab2-iCreF3;R26YFP* embryos at E17.5 and pups at 0 (P0) and 10 (P10) days of age. Scale bars 100 µm. Three representative images have been used per individual mouse. Source data for d is provided as a Source data file.

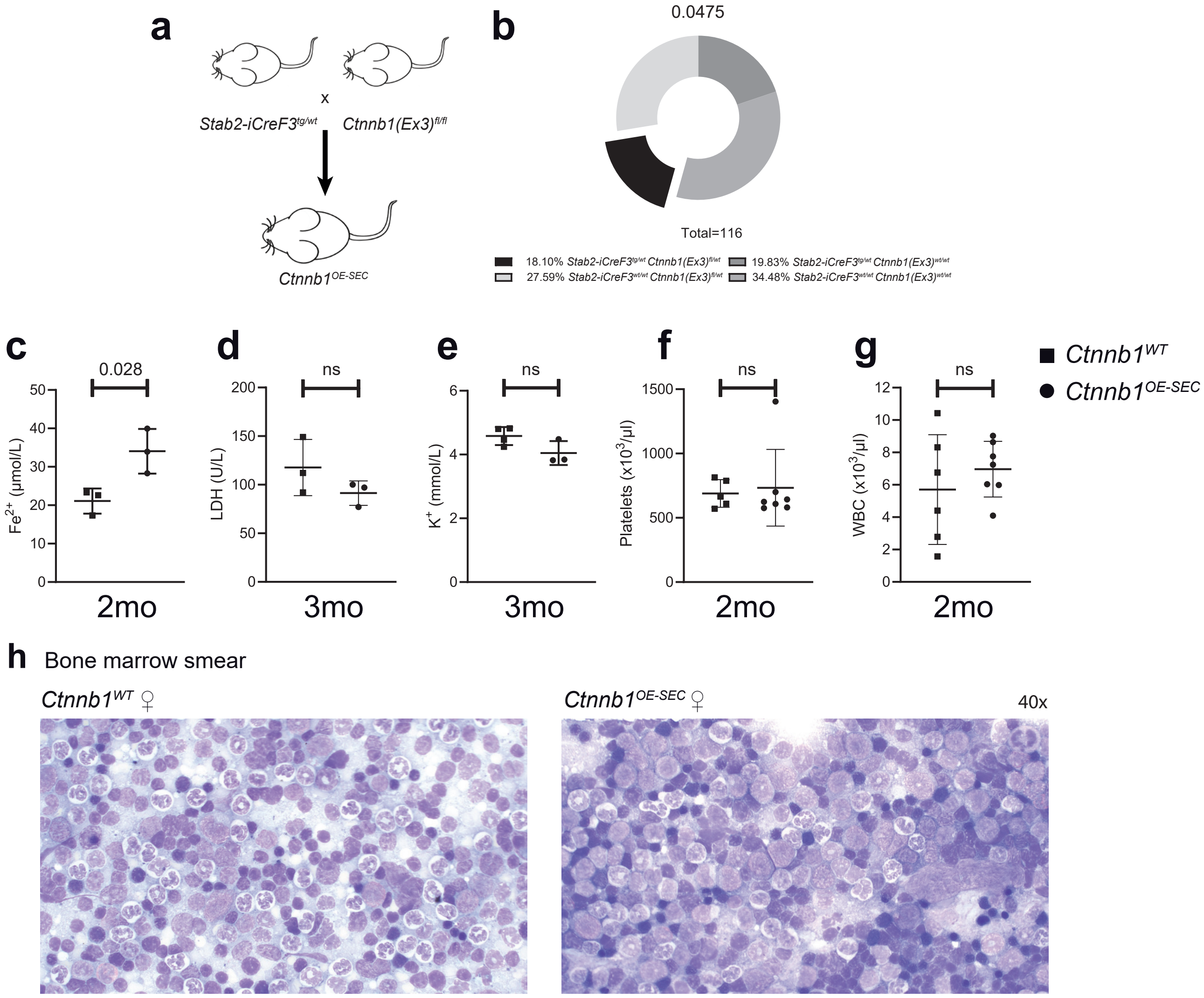

Supplementary Fig. 2 Breeding scheme, Mendelian frequency, and further blood parameters of *Ctnnb1^OE-SEC^* mice. **(a)** *Ctnnb1^OE-SEC^* mice (*Stab2-iCreF3^tg/wt^ Ctnnb1(ex3)^fl/wt^*) were produced by crossing *Stab2-iCreF3^tg/wt^* with *Ctnnb1(Ex3)^fl/fl^* mice and **(b)** Mendelian frequency of all progeny was determined. **(c)** Iron (Fe2+) (n = 3), **(d)** lactate dehydrogenase (LDH) (n = 3), **(e)** potassium (K^+) levels (n ≥ 3), **(f)** platelet (n = 5-7) and **(g)** white blood cell counts (WBC) (n = 6-7) measured in the peripheral blood of 2 and 3 months old female *Ctnnb1^WT^* and *Ctnnb1^OE-SEC^* mice. **(h)** Bone marrow smears of 5 months old male *Ctnnb1^WT^* and *Ctnnb1^OE-SEC^* (n = 3) mice (40x enlargement). Three representative images have been used per individual mouse. Mean ± s.e.m. is shown for each group of mice in all graphs. Statistical significance was determined using student's t-test or Mann–Whitney U test (two-sided). ns, not significant. Source data, precise values of 'n' and employed statistical tests for b, c, d, e, f, g are provided as a Source data file.

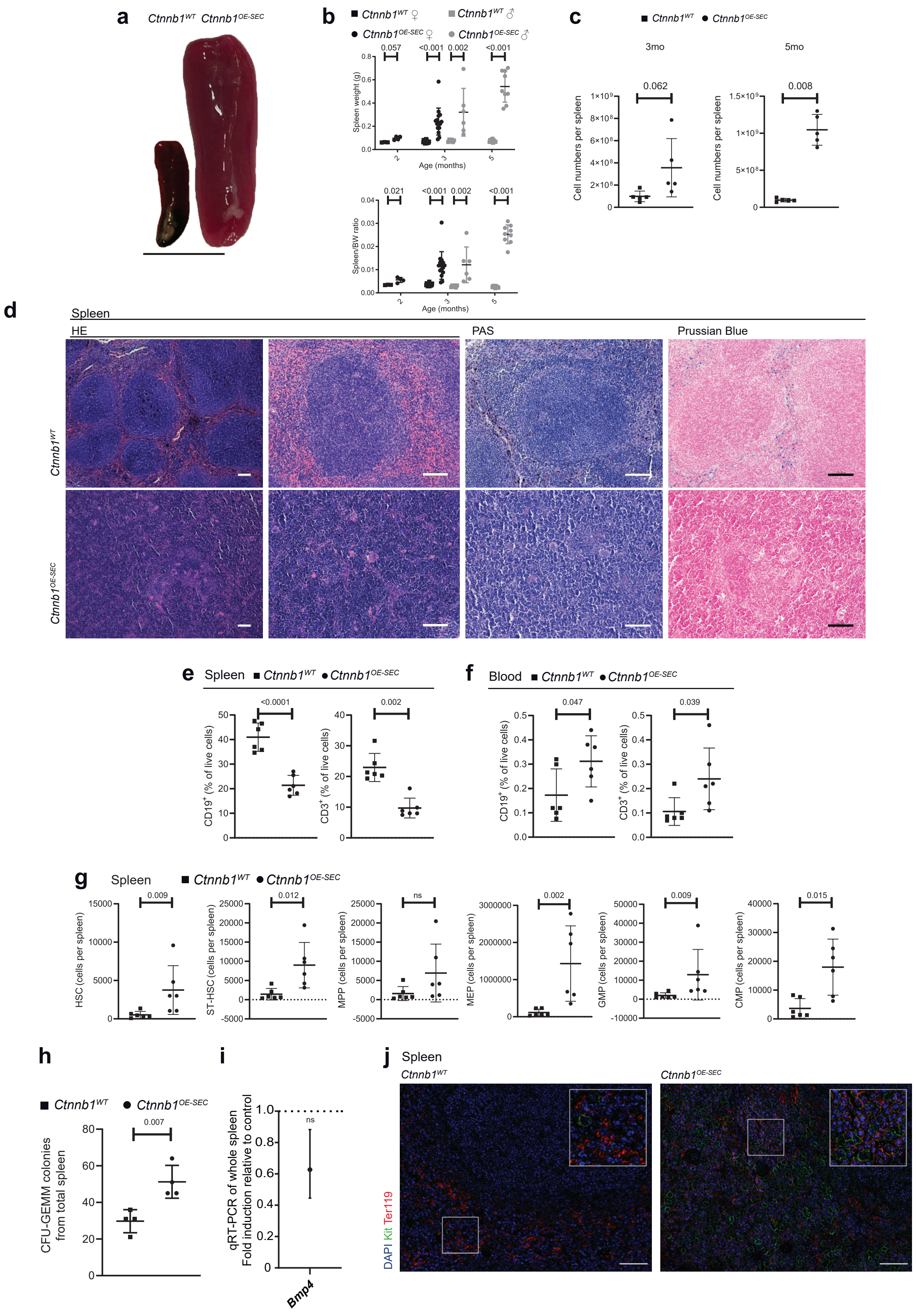

Supplementary Fig. 3 Anemia in *Ctnnb1*$^{OE\text{-}SEC}$ mice induces extramedullary hematopoiesis in spleen. (a) Representative macroscopic image of isolated spleens from 3 months old female *Ctnnb1*^{WT} and *Ctnnb1*$^{OE\text{-}SEC}$ mice. Scale bar 1 cm. **(b-c)** Spleen weight, spleen-to-body weight ratio (2 months old females, n = 3-4; 3 months old females, n = 17-15; 3 months old males, n = 6; 5 months old males, n = 9) (b) of 2, 3 and 5 months old and spleen cellularity shown as cell numbers per spleen (c) of 3 and 5 months old *Ctnnb1*^{WT} and *Ctnnb1*$^{OE\text{-}SEC}$ mice (male, female, each n = 5). **(d)** H&E, PAS and Prussian Blue staining of spleen sections of 3 months old female *Ctnnb1*^{WT} and *Ctnnb1*$^{OE\text{-}SEC}$ mice (n = 5). Scale bar 100 µm. **(e-f)** Flow cytometry quantification of B-cells (CD19^{+}) and T-cells (CD3^{+}) in (e) the spleen and in (f) the peripheral blood of 3 months old female *Ctnnb1*^{WT} and *Ctnnb1*$^{OE\text{-}SEC}$ mice (n = 6). **(g)** Flow cytometry quantification of hematopoietic stem and progenitor populations shown as cell numbers per spleen in 3 months old female *Ctnnb1*^{WT} and *Ctnnb1*$^{OE\text{-}SEC}$ mice (n = 6). **(h)** Numbers of *in vitro* CFU assays performed with isolated spleen cells from 3 months old *Ctnnb1*^{WT} and *Ctnnb1*$^{OE\text{-}SEC}$ females (n = 4). **(i)** qRT-PCR for *Bmp4* with cDNA from spleen lysates of 3 months old female *Ctnnb1*^{WT} and *Ctnnb1*$^{OE\text{-}SEC}$ (n = 5). *β-Actin* was used as housekeeping gene. **(j)** Co-IF of DAPI, Kit and Ter119 in spleen tissue of 3 months old female *Ctnnb1*^{WT} and *Ctnnb1*$^{OE\text{-}SEC}$ mice (n = 3). Scale bar 50 µm. Three representative images have been used per individual mouse. Mean ± s.e.m. is shown for each group of mice in b, c, e, f, g, h. Fold change ± s.e.m. is shown in i. Statistical significance was determined using student's t-test or Mann–Whitney U test (two-sided). ns, not significant. Source data, precise values of 'n' and employed statistical tests for b, c, e, f, g, h, i are provided as a Source data file.

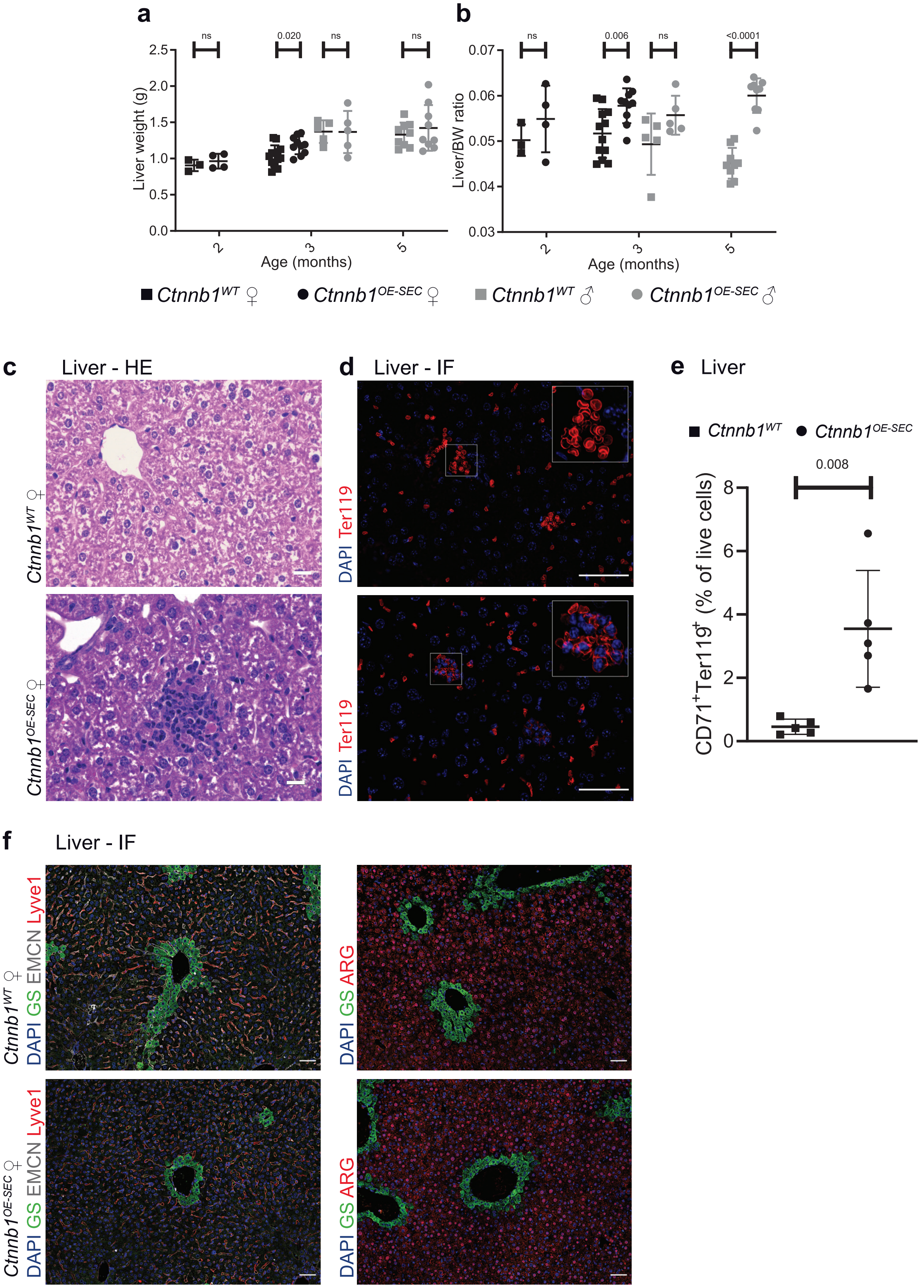

Supplementary Fig. 4 Anemia in *Ctnnb1*$^{OE\text{-}SEC}$ mice induces extramedullary hematopoiesis in the liver. (a) Liver weight, **(b)** liver-to-body weight ratio (2 months old females, n = 3-4; 3 months old females, n = 12-10; 3 months old males, n = 5; 5 months old males, n = 9) of 2, 3 and 5 months old *Ctnnb1*^{WT} and *Ctnnb1*$^{OE\text{-}SEC}$ mice. **(c)** H&E staining of liver sections of 3 months old female *Ctnnb1*^{WT} and *Ctnnb1*$^{OE\text{-}SEC}$ mice (n = 5). Scale bar 20 µm. **(d)** Co-IF of DAPI and Ter119 in the liver of 3 months old female *Ctnnb1*^{WT} and *Ctnnb1*$^{OE\text{-}SEC}$ mice (n = 3). Scale bar 50 µm. **(e)** Flow cytometry quantification of CD71+-Ter119+ cells in the liver 5 months old male *Ctnnb1*^{WT} and *Ctnnb1*$^{OE\text{-}SEC}$ mice (n = 5). **(f)** Co-IF of DAPI, glutamine synthetase (GS), EMCN and Lyve1 (f, left); DAPI, GS and arginase (Arg) (f, right) in the liver of 3 months old female *Ctnnb1*^{WT} and *Ctnnb1*$^{OE\text{-}SEC}$ mice (n = 3). Scale bar 50 µm. Three representative images have been used per individual mouse. Mean ± s.e.m. is shown for each group of mice in all graphs. Statistical significance was determined using student's t-test or Mann–Whitney U test (two-sided). ns, not significant. Source data, precise values of 'n' and employed statistical tests for a, b, e are provided as a Source data file.

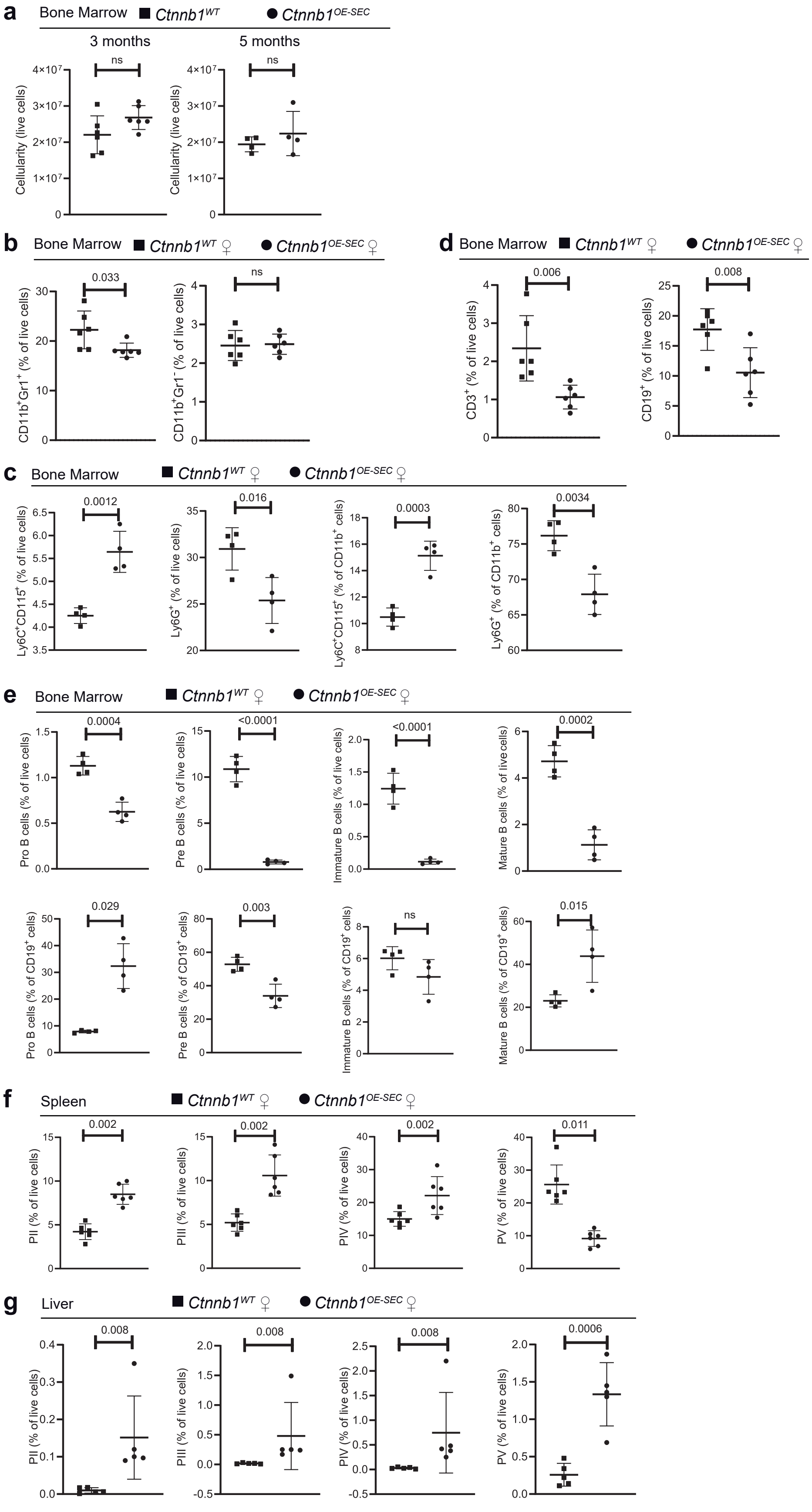

Supplementary Fig. 5 Flow cytometry quantification of different hematopoietic cells in the bone marrow, liver and spleen of *Ctnnb1^OE-SEC* mice. **(a)** Bone marrow cellularity, expressed as total live cells per 1x lower extremity, in 3 months old female (n = 6) and 5 months old male (n = 4) *Ctnnb1^WT* and *Ctnnb1^OE-SEC* mice. **(b)** Flow cytometry quantification of CD11b⁺Gr1⁺ and CD11b⁺Gr1⁻ cells in the bone marrow 3 months old female *Ctnnb1^WT* and *Ctnnb1^OE-SEC* mice (n = 6). **(c)** Flow cytometry quantification of Ly6C⁺CD115⁺ and Ly6G⁺ cells in the bone marrow 5 months old male *Ctnnb1^WT* and *Ctnnb1^OE-SEC* mice (n = 4). **(d)** Flow cytometry quantification of B-cells (CD19⁺) and T-cells (CD3⁺) in the bone marrow of 3 months old female *Ctnnb1^WT* and *Ctnnb1^OE-SEC* mice (n = 6). **(e)** Flow cytometry quantification of pro B cells, pre B cells, immature B cells and mature B cells in the bone marrow 5 months old male *Ctnnb1^WT* and *Ctnnb1^OE-SEC* mice (n = 4). **(f, g)** Flow cytometry quantification of PII – PV in (f) the spleen of 3 months old female *Ctnnb1^WT* and *Ctnnb1^OE-SEC* mice (n = 6) and in (g) the liver of 5 months old male *Ctnnb1^WT* and *Ctnnb1^OE-SEC* mice (n = 5). Mean ± s.e.m. is shown for each group of mice in all graphs. Statistical significance was determined using student's t-test or Mann–Whitney U test (two-sided). ns, not significant. Source data and employed statistical tests for a, b, c, d, e, f, g are provided as a Source data file.

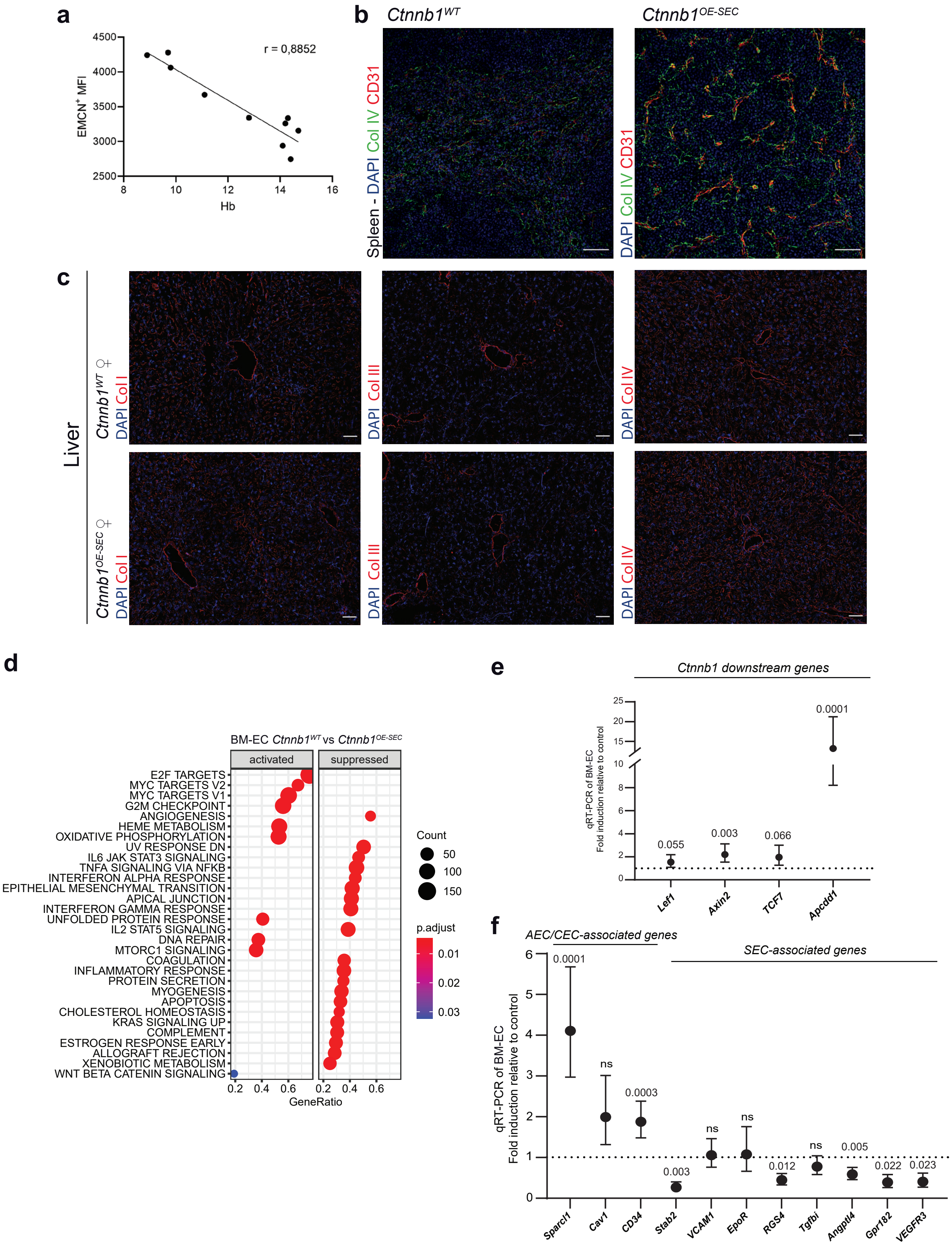

Supplementary Fig. 6 Characterization and analysis of endothelial cells in the bone marrow, spleen and liver of *Ctnnb1*$^{OE\text{-}SEC}$ mice. (a) Correlation of EMCN IF quantification and hemoglobin (Hb) blood levels of 3 months old female *Ctnnb1*^{WT} and *Ctnnb1*$^{OE\text{-}SEC}$ mice (n = 10). **(b)** Co-IF of DAPI, Collagen IV (Col IV) and CD31 in the spleen of 5 months old female *Ctnnb1*^{WT} and *Ctnnb1*$^{OE\text{-}SEC}$ mice (n = 3). Scale bar 50 µm. **(c)** Co-IF of DAPI and Collagen I (Col I), Collagen III (Col III) and Collagen IV (Col IV) in the liver of 3 months old female *Ctnnb1*^{WT} and *Ctnnb1*$^{OE\text{-}SEC}$ mice (n = 3). Scale bar 50 µm. **(d)** GSEA-KEGG pathway alterations analyzed using MSigDB hallmark gene sets in isolated BM-EC from bone marrow of 3 months old female *Ctnnb1*^{WT} and *Ctnnb1*$^{OE\text{-}SEC}$ mice (n = 6). **(e)** qRT-PCR for Ctnnb1-downstream genes *Lef1*, *Axin2*, *TCF7* and *Apccd1* with cDNA from freshly isolated BM-EC of 3 months old female *Ctnnb1*^{WT} and *Ctnnb1*$^{OE\text{-}SEC}$ mice (n = 6). *β-Actin* was used as housekeeping gene. **(f)** qRT-PCR for AEC/CEC- (*Sparcl1*, *Cav1*, *CD34*) and SEC-associated (*Stab2*,*VCAM1*, *EpoR*, *RGS4*, *Tgfbi*, *Angptl4*, *Gpr182*, *VEGFR3*) genes with cDNA from freshly isolated BM-EC of 3 months old female Ctnnb1WT and Ctnnb1OE-SEC mice (n = 6). *β-Actin* was used as housekeeping gene. Three representative images have been used per individual mouse. Fold change ± s.e.m. is shown in e, f. Statistical significance was determined using student's t-test. ns, not significant. Source data, precise values of 'n' and employed statistical tests for a, d, e, f are provided as a Source data file.

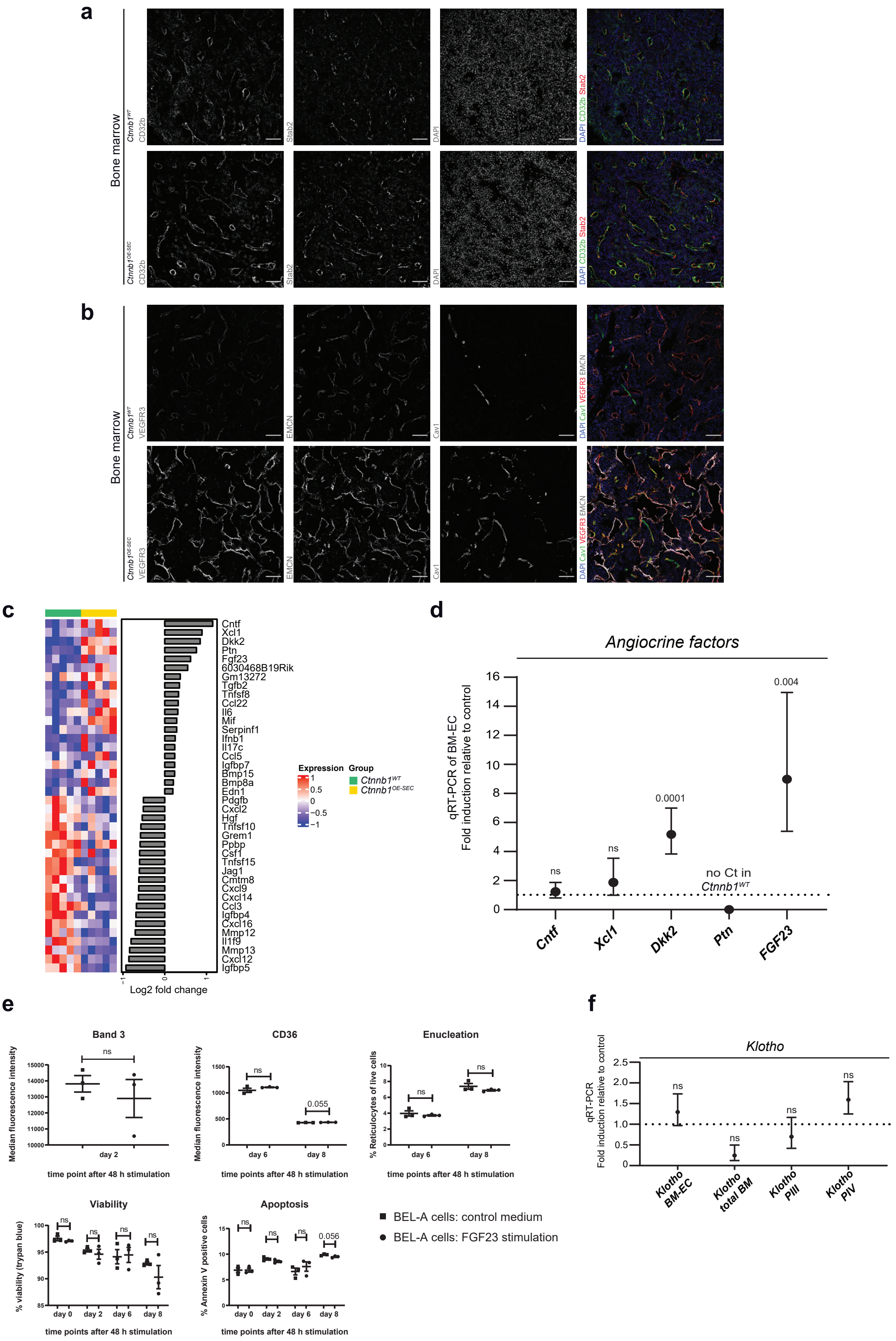

Supplementary Fig. 7 Characterization and analysis of endothelial cells in the bone marrow of *Ctnnb1*$^{OE\text{-}SEC}$ mice. (a, b) IF of DAPI, (a) CD32b, Stab2 of 3 months old female *Ctnnb1*^{WT} and *Ctnnb1*$^{OE\text{-}SEC}$ and (b) VEGFR3 and EMCN of 5 months old male *Ctnnb1*^{WT} and *Ctnnb1*$^{OE\text{-}SEC}$ bone marrow samples shown as single-channel and confocal photomicrographs (n = 3). Scale bars 50 µm. **(c)** Heat map of angiocrine factor genes. Selected genes[2] are shown for freshly isolated BM-EC from *Ctnnb1*^{WT} (green) and *Ctnnb1*$^{OE\text{-}SEC}$ mice (yellow). The heat map color represents the mean and maximum values for each gene. The intensity scale of the standardized expression values ranges from dark blue (low expression) to dark red (high expression). **(d)** qRT-PCR for angiocrine factos genes *Cntf*, *Xcl1*, *Dkk2*, *Ptn* and *FGF23* with cDNA from freshly isolated BM-EC of 3 months old female *Ctnnb1*^{WT} and *Ctnnb1*$^{OE\text{-}SEC}$ mice (n = 6). *β-Actin* was used as housekeeping gene. **(e)** *In vitro* stimulation of BEL-A cells with FGF23 for 48 hours prior to analysis. Assessment of differentiation via Band3 and CD36 level measurement, of enucleation via measurement of reticulocyte numbers, of cell viability via tryptan blue staining and of apoptosis via Annexin V measurement (n = 3). **(f)** qRT-PCR for *Klotho* with cDNA from freshly isolated BM-EC, total bone marrow, PIII and PIV of *Ctnnb1*^{WT} and *Ctnnb1*$^{OE\text{-}SEC}$ mice (n = 6). *β-Actin* was used as housekeeping gene. Three representative images have been used per individual mouse. Mean ± s.e.m. is shown for each group of mice in e. Fold change ± s.e.m. is shown in d, f. Statistical significance was determined using student's t-test. ns, not significant. Source data and employed statistical tests for c, d, e, f are provided as a Source data file.

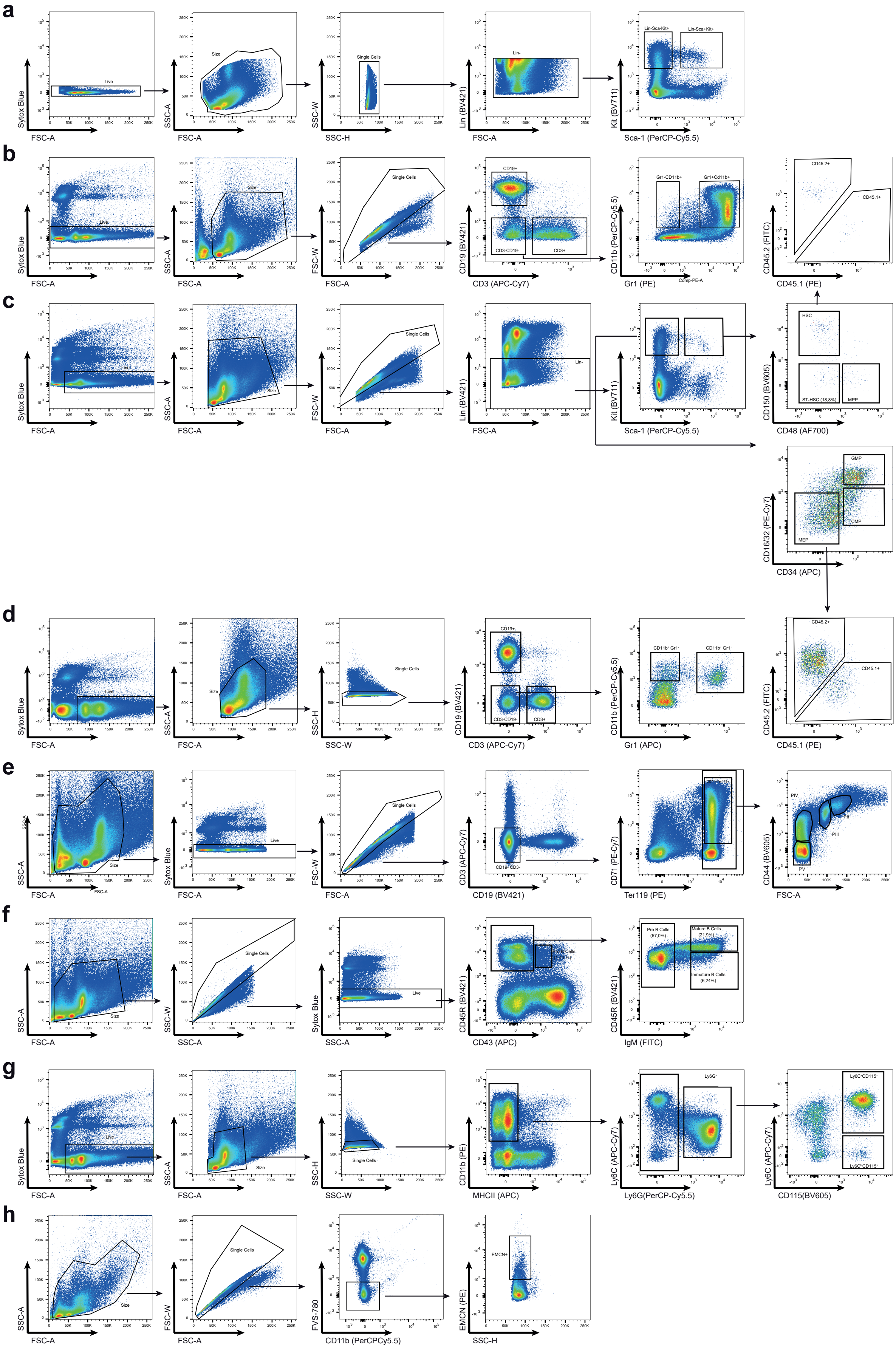

Supplementary Fig. 8 FACS gating strategies. (a-h) Gating strategies for flow cytometry analysis of different cell populations are depicted. (a) Gating strategy for Kit$^+$Sca1$^+$ stem cells and Kit$^+$Sca1$^-$ myeloid progenitor cells of YFP reporter mice are depicted, referring to Fig. 1g, 1h and Supp. Fig. 1d. (b) Gating strategy for CD3$^+$, CD19$^+$, CD11b$^+$Gr$^-$ and CD11b$^+$Gr$^+$ populations of YFP reporter mice are depicted, referring to Fig. 1g, 1h and Supp. Fig 1d. (c) Gating strategy for HSC, ST-HSC and MPP stem cell populations as well as MEP, CMP and GMP myeloid progenitors in bone marrow and spleen, referring to Fig. 3a, 3j, Supp. Fig. 3g. CD45.1 and CD45.2 analysis of HSC and MEP compartments, referring to Fig. 3k. (d) Gating strategy for CD3$^+$, CD19$^+$, CD11b$^+$Gr$^-$ and CD11b$^+$Gr$^+$ populations in bone marrow and spleen, referring to Supp. Fig. 3e, 3f and 5b. (e) Gating strategy for CD71$^+$Ter119$^+$ cells as well as erythroid progenitor populations PII, PIII, PIV and PV in bone marrow, spleen and liver, referring to Fig. 3d, Supp. Fig. 5f and 5g. (f) Gating strategy for Pro B Cells, Pre B Cells, Immature B Cells and Mature B Cells in bone marrow, referring to Supp. Fig. 5e (g) Gating strategy for Ly6G$^+$, Ly6C$^+$CD115$^+$ and Ly6CloCD115$^+$ cells in bone marrow, referring to Supp. Fig 5c. (h) Gating strategy for EMCN$^+$ cells in bone marrow, referring to Supp. Fig 6a.

Supplementary Table 1. Quantitative Real-time Reverse Transcription-PCR Primer Sequence Information.

Gene	Forward sequence	Reverse sequence
Bmp4	5'- TAAACCGTCTTGGAGCCTGC-3'	5'- AATGGCACTACGGAATGGCT-3'
Lef1	5'-CTAGGCGCGGCGAGGAG-3'	5'-CGGGAATGTCCGAATGCCA-3'
Axin2	5'-AAAATAAGCAGCCGTTCGC-3'	5'-CTTAAGTCAGCAGGGGCTCAT-3'
TCF7	5'- CGATCTCTCTGGATTTTATTCTCT -3'	5'- TGCTGTCTATATCCGCAGGAAG-3'
Apcdd1	5'-GGGACAGAGACGGACTACAGC-3'	5'-TAAGGACCGAGGGTGCGAT-3'
Sparcl1		
Cav1	5'- CCCTGGCGAACAGCCAAGA-3'	5'- CGTCGTCGTTGAGATGCTTG-3'
CD34		
Stab2	5'- CTCCTGGCACTCATCAGAGG-3'	5'- AGAAGCTTGCTCCTTGCCAT-3'
VCAM1	5'-TGCCGGCATATACGAGTGTG-3'	5'-AAACGATCATCCCGATGGCA-3'
EpoR	5'-ATGGACAAACTCAGGGTGCC-3'	5'-AAGTCTTCCAAGCGTTGGGT-3'
RGS4	5'-ACACATTCGTGCAACACTGC-3'	5'-GCCCGGTACATTGGCTTACT-3'
Tgfbi	5'-CCCGGAAGCTTCACCATCTT-3'	5'-CGATGTTGACGTTGCTCACC-3'
Angptl4	5'- TTTGGTACCTGTAGCCATTC-3'	5'- ATACCCTTTTTACGCTCCTG-3'

Gpr182	5'-CCACCTTGGAACCGGACAAT-3'	5'-AAGCCTACCACGAAGATGGC-3'
VEGFR3	5'-CTGGCAAATGGTTACTCCATGA-3'	5'-ACAACCCGTGTGTCTTCACTG-3'
Cntf	5'-TCTGTAGCCGCTCTATCTGG-3'	5'-GGTACACCATCCACTGAGTCAA-3'
Xcl1	5'-TTTGTCACCAAACGAGGACTAAA-3'	5'-CCAGTCAGGGTTATCGCTGTG-3'
Dkk2	5'-CTGATGCGGGTCAAGGATTCA-3'	5'-CTCCCCTCCTAGAGAGGACTT-3'
Ptn	5'-CTCTGCACAATGCTGACTGTC-3'	5'-ACAGCTTCTTACCTTGAGGCTT-3'
FGF23	5'-ATGGTCATGTAGATGGCACCC-3'	5'-CTTCGAGTCATGGCTCCTGTT-3'
Klotho	5'-AAATGGCTGGTTTGTCTCGGGAAC-3'	5'-TATGCCACTCGAAACCGTCCATGA-3'
β-Actin	5'-ACCCGCGAGCACAGCTTC-3'	5'-CTTTGCACATGCCGGAGC-3'

Supplementary References

1 Koch, P. S. *et al.* Angiocrine Bmp2 signaling in murine liver controls normal iron homeostasis. *Blood* **129**, 415-419, doi:10.1182/blood-2016-07-729822 (2017).

2 Winkler, M. *et al.* Endothelial GATA4 controls liver fibrosis and regeneration by preventing a pathogenic switch in angiocrine signaling. *J Hepatol*, doi:10.1016/j.jhep.2020.08.033 (2020).
