# Supplementary material for: Bone marrow sinusoidal endothelium controls terminal erythroid differentiation and reticulocyte maturation
Source: Nat Commun. 2021 Nov 29;12:6963. doi: 10.1038/s41467-021-27161-3 (PMC8630019; doi:10.1038/s41467-021-27161-3)
Supplement: Supplementary file 2 — Reporting Summary [file 41467_2021_27161_MOESM2_ESM.pdf]

nature portfolio

Corresponding author(s): PD Dr. Philipp-Sebastian Reiners-Koch

Last updated by author(s): Oct 26, 2021

Reporting Summary

Nature Portfolio wishes to improve the reproducibility of the work that we publish. This form provides structure for consistency and transparency in reporting. For further information on Nature Portfolio policies, see our Editorial Policies and the Editorial Policy Checklist.

Statistics

For all statistical analyses, confirm that the following items are present in the figure legend, table legend, main text, or Methods section.

n/a	Confirmed	
☐	☒	The exact sample size (*n*) for each experimental group/condition, given as a discrete number and unit of measurement
☐	☒	A statement on whether measurements were taken from distinct samples or whether the same sample was measured repeatedly
☐	☒	The statistical test(s) used AND whether they are one- or two-sided<br>*Only common tests should be described solely by name; describe more complex techniques in the Methods section.*
☐	☒	A description of all covariates tested
☐	☒	A description of any assumptions or corrections, such as tests of normality and adjustment for multiple comparisons
☐	☒	A full description of the statistical parameters including central tendency (e.g. means) or other basic estimates (e.g. regression coefficient) AND variation (e.g. standard deviation) or associated estimates of uncertainty (e.g. confidence intervals)
☐	☒	For null hypothesis testing, the test statistic (e.g. *F*, *t*, *r*) with confidence intervals, effect sizes, degrees of freedom and *P* value noted<br>*Give P values as exact values whenever suitable.*
☒	☐	For Bayesian analysis, information on the choice of priors and Markov chain Monte Carlo settings
☒	☐	For hierarchical and complex designs, identification of the appropriate level for tests and full reporting of outcomes
☒	☐	Estimates of effect sizes (e.g. Cohen's *d*, Pearson's *r*), indicating how they were calculated

Our web collection on statistics for biologists contains articles on many of the points above.

Software and code

Policy information about availability of computer code

Data collection	FACS Diva Software v6.1.3 (Flowcytometers and sorters, BD Bioscience); Image Processing NIS-Elements Imaging Software AR 5.02.00
Data analysis	FlowJo, Versions 10.6.1 and 10.7.1, Statistical analysis with Graphpad Prism v9.1.2 and SigmaPlot 11 Software (Systat Software GmbH); SAS JMP15 Genomics, Version 10; image Processing with ImageJ 1.52e Software

For manuscripts utilizing custom algorithms or software that are central to the research but not yet described in published literature, software must be made available to editors and reviewers. We strongly encourage code deposition in a community repository (e.g. GitHub). See the Nature Portfolio guidelines for submitting code & software for further information.

Data

Policy information about availability of data

All manuscripts must include a data availability statement. This statement should provide the following information, where applicable:

- Accession codes, unique identifiers, or web links for publicly available datasets
- A description of any restrictions on data availability
- For clinical datasets or third party data, please ensure that the statement adheres to our policy

Data Availability. The raw and normalized microarray data of isolated PIII, PIV and BM endothelial cells generated in this study have been deposited in the Gene Expression Omnibus database under the accession number GSE180522 [http://www.ncbi.nlm.nih.gov/geo/] and made publicly available. The processed data of statistically significant differentially expressed genes of isolated erythroid PIII and PIV populations are provided in the Supplementary Data files 1 and 2.

Field-specific reporting

Please select the one below that is the best fit for your research. If you are not sure, read the appropriate sections before making your selection.

☒ Life sciences ☐ Behavioural & social sciences ☐ Ecological, evolutionary & environmental sciences

For a reference copy of the document with all sections, see nature.com/documents/nr-reporting-summary-flat.pdf

Life sciences study design

All studies must disclose on these points even when the disclosure is negative.

Sample size	No sample size calculation was performed. Sample size for in vivo experiments were determined using previous studies (minimum 3 animals per genotype, age and sex).
Data exclusions	There have been no data samples excluded.
Replication	At least three replicates per analysed group have been used for statistical analysis. We analyzed a sufficient number of mice (3-30 mice per group) to ensure that the results are representative.
Randomization	There was no randomization, since replicate groups have been chosen according to their phenotype.
Blinding	The investigators of this study were not blinded for analysis of FACS, ELISA and stainings including microscopy imaging since the same gating or protocols/settings were used for both groups. The investigators of the blood analysis were blinded to group allocation and data analysis.

Reporting for specific materials, systems and methods

We require information from authors about some types of materials, experimental systems and methods used in many studies. Here, indicate whether each material, system or method listed is relevant to your study. If you are not sure if a list item applies to your research, read the appropriate section before selecting a response.

Materials & experimental systems

n/a	Involved in the study	
☐	☒	Antibodies
☐	☒	Eukaryotic cell lines
☒	☐	Palaeontology and archaeology
☐	☒	Animals and other organisms
☒	☐	Human research participants
☒	☐	Clinical data
☒	☐	Dual use research of concern

Methods

n/a	Involved in the study	
☒	☐	ChIP-seq
☐	☒	Flow cytometry
☒	☐	MRI-based neuroimaging

Antibodies

Antibodies used	Primary antibodies: Monoclonal rat anti-Endomucin (Clone V.7C7, Cat. No. 14-5851-82, eBioscience; dilution 1:100), polyclonal chicken anti-GFP/YFP (Cat. No. ab13970, abcam; dilution 1:800), polyclonal goat anti-CD32 (Cat. No. AF1460, R&DSystems; dilution 1:75), polyclonal rabbit anti-alpha smooth muscle Actin (Cat. No. ab5694, abcam; dilution 1:500), monoclonal rat anti-VEGFR-3 (Cat. No. 552857, BD Pharmigen; dilution 1:100), polyclonal rabbit anti-Cav1 (Cat. No. sc-7305, Santa Cruz; dilution 1:250), monoclonal rat anti-CD45 (Clone 30-F11, Cat. No. 550539, , BD Pharmigen; dilution 1:400), monoclonal rat anti-Ter119 (Clone TER-119, Cat. No. 550565, BD Pharmingen; dilution 1:200), polyclonal goat anti-c-kit-antibody (Cat. No. AF1356, R&D Systems; dilution 1:200), monoclonal rat anti-CD31 (Clone MEC13.3, Cat. No. 102502, BioLegend; dilution 1:300), polyclonal rabbit anti-Collagen type I (Cat. No. R1038, Acris; dilution 1:200), polyclonal rabbit anti-Collagen type III alpha 1 chain (Cat. No. R1040, Acris; dilution 1:200), polyclonal rabbit anti-Collagen IV (Cat. No. GTX19808, Genetex; dilution 1:200), polyclonal rabbit anti-fibronectin (Cat. No. ab23750, Abcam; dilution 1:100), polyclonal goat anti-Podocalyxin (Cat. No. AF1556, R&D Systems; dilution 1:100), polyclonal rabbit anti-Glutamine Synthetase (Cat. No. G2781, Sigma-Aldrich; dilution 1:2000), polyclonal goat anti-Arginase I (Cat. No. sc-18351, Santa Cruz; dilution 1:50), polyclonal goat anti-Lyve1 (Cat. No. AF2125, R&D Systems; dilution 1:400), mouse anti–Stabilin-2 clone 3.1 antibody (dilution 1:700). The following secondary antibodies were all purchased from Dianova and diluted 1:400: Alexa-Fluor 488 (donkey-anti-chicken (Cat. No. 703-545-155), donkey-anti-goat (Cat. No. 705-545-147), donkey-anti-rabbit (Cat. No. 711-545-152), donkey-anti-rat (Cat. No. 712-545-153), Alexa-Fluor 647 (donkey-anti-chicken (Cat. No. 703-605-155), donkey-anti-goat (Cat. No. 705-605-147), donkey-anti-rabbit (Cat. No. 711-605-152), donkey-anti-rat (Cat. No. 712-605-153), and Cy3 (donkey-anti-chicken (Cat. No. 703-165-155), donkey-anti-goat (Cat. No. 705-165-147), donkey-anti-rabbit (Cat. No. 711-165-152), donkey-anti-rat (Cat. No. 712-165-153). For FACS staining the following antibodies were used: monoclonal rat anti-CD3ε–APC–eFluor 780 (Clone 17A2, Cat. No. 47-0032-82, eBioscience; dilution 1:25), monoclonal rat anti-CD3ε–PE (Clone 145-2C11, Cat. No. 553063, BD Pharmingen; dilution 1:25), monoclonal rat anti-CD3ε–BV510 (Clone 17-A2, Cat. No. 100233, BioLegend; dilution 1:100), monoclonal rat anti-CD16/32–PE–Cy7 (Clone 93, Cat. No. 25-0161-82, eBioscience; dilution 1:400), monoclonal rat anti-CD34–eFluor660 (Clone RAM34, Cat. No. 50-0341, eBioscience; dilution 1:400), monoclonal rat anti-CD48-AF700 (Clone HM48-1, Cat. No. 103426, BioLegend; dilution

1:400), monoclonal rat anti-Sca1–PerCP–Cy5.5 (Clone D7, Cat. No. 45-5981, eBioscience; dilution 1:400), monoclonal rat anti-CD19–BV421 (Clone 6D5, Cat. No. 50-0341, eBioscience; dilution 1:400), monoclonal rat anti-CD19–BV605 (Clone 6D5, Cat. No. 115540, BioLegend; dilution 1:800), monoclonal rat anti-CD19–PerCPCy5.5 (Clone 6D5, Cat. No. 115534, BioLegend; dilution 1:100), monoclonal rat anti-CD117–BV711 (Clone 2B8, Cat. No. 105835, BioLegend; dilution 1:800), monoclonal rat anti-CD150–BV605 (Clone TC15–12F12.2, Cat. No. 115927, BioLegend; dilution 1:100), monoclonal rat anti-Gr1-APC (Clone RB6-8C5, Cat. No. 553129, BD Pharmingen; dilution 1:400), monoclonal rat anti-Gr1-PE (Clone RB6-8C5, Cat. No. 553128, BD Pharmingen; dilution 1:400), monoclonal rat anti-CD45-PE-Cy7 (Clone 30-F11, Cat. No. 25-0451-82, eBioscience; dilution 1:400), monoclonal rat anti-CD45-APC (Clone 30-F11, Cat. No. 559864, BD Pharmingen; dilution 1:50), monoclonal rat anti-CD11b-PerCP-Cy5.5 (Clone M1/70, Cat. No. 45-0112-82, eBioscience; dilution 1:400), monoclonal rat anti-CD11b-PE (Clone M1/70, Cat. No. 12-0112-82, eBioscience; dilution 1:800), monoclonal rat anti-CD44–BV605 (Clone IM7, Cat. No. 103047, BioLegend; dilution 1:400), monoclonal rat anti-Ter119–PE (Clone Ter119, Cat. No. 553673, BD Pharmingen; dilution 1:50), monoclonal rat anti-Ter119–APC-Cy7 (Clone Ter119, Cat. No. 116223, BioLegend; dilution 1:100), monoclonal rat anti-CD71–PE-Cy7 (Clone RI7217, Cat. No. 113811, BioLegend; dilution 1:100), monoclonal rat anti-CD71–PE (Clone RI7217, Cat. No. 113808, BioLegend; dilution 1:100), monoclonal rat anti-CD45.1-BV785 (Clone A20, Cat. No. 110743, BioLegend; dilution 1:100), monoclonal rat anti-CD45.2-BV711 (Clone 104, Cat. No. 563685, eBioscience; dilution 1:400) monoclonal rat anti-CD45R-BV421 (Clone RA3-6B2, Cat. No. 103251, BioLegend; 1:100), monoclonal rat anti-IgM-FITC (Clone 121-15F9, Cat. No. 11-5890-85, eBioscience; dilution 1:100), monoclonal rat anti-MHCII-APC (Clone M5/114.15.2, Cat. No. 17-5321-81, eBioscience; dilution 1:400), monoclonal rat anti-Ly6C-APC-Cy7 (Clone AL-21, Cat. No. 560596, BD Pharmingen; dilution 1:100), monoclonal rat anti-Ly6G-PerCP-Cy5.5 (Clone 1A8, Cat. No. 560602, BD Pharmingen; dilution 1:100), monoclonal rat anti-CD115-BV605 (Clone AFS98, Cat. No. 135517, BioLegend; dilution 1:400) monoclonal rat anti-EMCN-PE (Clone V.7C7, Cat. No. 12-5851-82, eBioscience).
The lineage cocktail was composed of monoclonal rat anti-CD3ε-BV421 (Clone 17A2, Cat. No. 100228, BioLegend; dilution 1:200), monoclonal rat anti-CD4-BV421 (Clone GK1.5, Cat. No. 100443, BioLegend; dilution 1:800), monoclonal rat anti-CD8-BV421 (Clone 53-6.7, Cat. No. 100753, BioLegend; dilution 1:800), monoclonal rat anti-CD11b-BV421 (Clone M1/70, Cat. No. 101251, BioLegend; dilution 1:800), monoclonal rat anti-CD19-BV421 (Clone 6D5, Cat. No. 115549, BioLegend; dilution 1:400), monoclonal rat anti-Gr-1-BV421 (Clone RB6-8C5, Cat. No. 108445, BioLegend; dilution 1:800) and monoclonal rat anti-Ter119-BV421 (Clone Ter119, Cat. No. 116234, BioLegend; dilution 1:200). Sytox blue (Cat. No. S34857, ThermoFisher Scientific; dilution 1:25000) and Fixable Viability Dye eFluor 780 (Cat. No. 65-0865-18, eBioscience; dilution 1:1000) were used for dead cell discrimination. All populations were gated for live single cells. The gating strategies are shown in Supplementary Fig. 8a-h. Stem cell populations were defined as: HSCs (Lin-Kit+Sca-1+CD150+CD48-), ST-HSCs (Lin-Kit+Sca-1+CD150-CD48-), MPPs (Lin-Kit+Sca-1+CD150-CD48+), CMPs (Lin-Kit+Sca-1-CD34+CD16/32low), GMPs (Lin-Kit+Sca-1-CD34+CD16/32+), MEPs (Lin-Kit+Sca-1-CD34-CD16/32-). Erythroid progenitors were defined as: PII (Basophilic erythroblasts: CD3-, CD19-, CD71+, Ter119+, CD44+, FSC+), PIII (Polychromatic erythroblasts: CD3-, CD19-, CD71+, Ter119+, CD44med, FSCmed), PIV (Orthochromatic erythroblasts and reticulocytes: CD3-, CD19-, CD71+, Ter119+, CD44low, FSClow), PV (Erythrocytes: CD3-, CD19-, CD71-, Ter119+, CD44-, FSClow). EMCN+ cells were defined as CD11b-EMCN+. Immune cells were defined as: CD3+ (Size/CD3+CD19-), CD19+ (Size/CD3-CD19+), CD11b+Gr- (Size/CD3-CD19-CD11b+Gr-), CD11b+Gr+ (Size/CD3-CD19-CD11b+Gr+), Pro B Cells (CD43midCD45R+), Pre B Cells (CD43-CD45R+IgM-), Immature B Cells (CD43-CD45RloIgM+), Mature B Cells (CD43-CD45RhiIgM+), Ly6G+ (Size/Ly6CmidLy6G+), Ly6CloCD115+ (Size/Ly6CloLy6G-CD115+), Ly6C+CD115+ (Size/Ly6C+Ly6G-CD115+).

Validation

IF
Monoclonal rat anti-Endomucin (Clone V.7C7, Cat. No. 14-5851-82, eBioscience; dilution 1:100) is validated by the vendor using Western blot analysis of differential expression of the target protein in mouse kidney versus mouse bone marrow lysates. (https://www.thermofisher.com/antibody/product/Endomucin-Antibody-clone-eBioV-7C7-V-7C7-Monoclonal/14-5851-82).
polyclonal chicken anti-GFP/YFP (Cat. No. ab13970, abcam; dilution 1:800) is validated by the vendor by immune fluorescence staining using transgenic cell lines expressing variants of the green fluorescent protein that are derived from the jellyfish Aequorea Victoria. (https://www.abcam.com/GFP-antibody-ab13970.html?gclsrc=aw.ds|aw.ds&gclid=EAIaIQobChMIzsOlj_Dl8wIV0xoGAB1OPQF2EAAYASAAEgLi5vD_BwE).
polyclonal goat anti-CD32 (Cat. No. AF1460, R&DSystems; dilution 1:75), is validated by the vendor by immune fluorescence staining using mouse splenocytes. (https://www.rndsystems.com/products/mouse-fcgamma-rii-riii-cd32-cd16-antibody_af1460).
polyclonal rabbit anti-alpha smooth muscle Actin (Cat. no. ab5694, abcam; dilution 1:500) is validated by the vendor using Western blot analysis of alpha Actin transgenic HEK-293 cells. (https://www.abcam.com/alpha-smooth-muscle-actin-antibody-ab5694.html).
monoclonal rat anti-VEGFR-3 (Cat. No. 552857, BD Pharmigen; dilution 1:100), is validated by the vendor using immunohistochemistry on paraffin sections of mouse liver with appropriate controls. (https://www.bdbiosciences.com/en-de/products/reagents/microscopy-imaging-reagents/immunohistochemistry-reagents/purified-rat-anti-mouse-vegfr-3.552857).
polyclonal rabbit anti-Cav1 (Cat. No. sc- sc-894, Santa Cruz; dilution 1:250) is validated by the vendor using Western blot analysis of caveolin-1 expression in CHO-K cells with appropriate controls. (https://www.scbt.com/p/caveolin-1-antibody-n-20).
monoclonal rat anti-CD45 (Clone 30-F11, Cat. No. 550539, BD Pharmigen; dilution 1:400) is validated by the vendor using immunohistochemistry staining of acetone-fixed frozen sections of mouse spleen. (https://www.bdbiosciences.com/en-us/products/reagents/microscopy-imaging-reagents/immunohistochemistry-reagents/purified-rat-anti-mouse-cd45.550539).
monoclonal rat anti-Ter119 (Clone TER-119, Cat. No. 550565, BD Pharmingen; dilution 1:200) is validated by the vendor using immunohistochemistry staining of formalin-fixed paraffin sections of mouse kidney. (https://www.bdbiosciences.com/en-de/products/reagents/flow-cytometry-reagents/research-reagents/single-color-antibodies-ruo/purified-rat-anti-mouse-ter-119-erythroid-cells.550565).
polyclonal goat anti-c-kit-antibody (Cat. No. AF1356, R&D Systems; dilution 1:200) is validated by the vendor using Western blot analysis on megakaryocyte cell lines and immunohistochemistry of mouse embryo frozen sections. (https://www.rndsystems.com/products/human-mouse-cd117-c-kit-antibody_af1356).
monoclonal rat anti-CD31 (Clone MEC13.3, Cat. No. 102502, BioLegend; dilution 1:300), is validated by the vendor using flow cytometry analysis of mouse splenocytes. (https://www.biolegend.com/en-us/products/purified-anti-mouse-cd31-antibody-380?GroupID=BLG10531).
polyclonal rabbit anti-Collagen type I (Cat. No. R1038, Acris; dilution 1:200), is validated by the vendor using ELISA against purified reference proteins. (https://www.origene.com/catalog/antibodies/primary-antibodies/r1038/collagen-i-col1a1-rabbit-polyclonal-antibody).
polyclonal rabbit anti-Collagen type III alpha 1 chain (Cat. No. R1040, Acris; dilution 1:200) is validated by the vendor using ELISA against purified reference proteins. (https://www.origene.com/catalog/antibodies/primary-antibodies/r1040/collagen-iii-col3a1-rabbit-polyclonal-antibody).
polyclonal rabbit anti-Collagen IV (Cat. No. GTX19808, Genetex; dilution 1:200) is validated by the vendor using ELISA against purified

standards. (https://www.genetex.com/Product/Detail/Collagen-IV-antibody/GTX19808).
polyclonal rabbit anti-fibronectin (Cat. No. ab23750, Abcam; dilution 1:100) is validated by the vendor using automatic immunohistochemistry staining of formalin-fixed paraffin sections of human aorta. (https://www.abcam.com/fibronectin-antibody-bsa-and-azide-free-ab23750.html#lb).
polyclonal goat anti-Podocalyxin (Cat. No. AF1556, R&D Systems; dilution 1:100) is validated by the vendor using direct ELISAs and Western blots. (https://www.rndsystems.com/products/mouse-podocalyxin-antibody_af1556).
polyclonal rabbit anti-Glutamine Synthetase (Cat. No. G2781, Sigma-Aldrich; dilution 1:2000) is validated by the vendor using Western blot analysis on rat brain lysates and immunohistochemistry of formalin-fixed rat brain sections. (https://www.sigmaaldrich.com/DE/de/product/sigma/g2781).
polyclonal goat anti-Arginase I (Cat. No. sc-18351, Santa Cruz; dilution 1:50) is validated by the vendor using Western blot analysis on human liver and immunohistochemistry of formalin-fixed human liver sections. (https://www.scbt.com/p/arginase-i-antibody-n-20).
polyclonal goat anti-Lyve1 (Cat. No. AF2125, R&D Systems; dilution 1:400), is validated by the vendor using Western blot analysis on mouse liver and immunohistochemistry of formalin-fixed mouse liver sections. (https://www.rndsystems.com/products/mouse-lyve-1-antibody_af2125).
mouse anti–Stabilin-2 clone 3.1 antibody (dilution 1:700) is validated by our laboratory using direct ELISA against purified Stabilin-2 protein and immunohistochemistry staining of formalin-fixed mouse liver sections against appropriate stabilin-2 knockout controls 28.
Secondary antibodies:
Cross adsorbed fluorescence labelled secondary antybodies were all purchased from Jackson ImmunoResearch Labs via Dianova and diluted 1:400:
Donkey IgG anti-Chicken IgY (H+L)-Alexa Fluor® 488 (Cat. No. 703-545-155),
Donkey IgG anti-Goat IgG (H+L)-Alexa Fluor® 488 (Cat. No. 705-545-147),
Donkey IgG anti-Rabbit IgG (H+L)-Alexa Fluor® 488 (Cat. No. 711-545-152),
Donkey IgG anti-Rat IgG (H+L)-Alexa Fluor® 488 (Cat. No. 712-545-153),
Donkey IgG anti-Chicken IgY (H+L)-Alexa Fluor® 647 (Cat. No. 703-605-155),
Donkey IgG anti-Goat IgG (H+L)-Alexa Fluor® 647 (Cat. No. 705-605-147),
Donkey IgG anti-Rabbit IgG (H+L)-Alexa Fluor® 647 (Cat. No. 711-605-152),
Donkey IgG anti-Rat IgG (H+L)-Alexa Fluor® 647 (Cat. No. 712-605-153),
Donkey IgG anti-Chicken IgY (H+L)-Cy3 (Cat. No. 703-165-155),
Donkey IgG anti-Goat IgG (H+L)-Cy3 (Cat. No. 705-165-147),
Donkey IgG anti-Rabbit IgG (H+L)-Cy3 (Cat. No. 711-165-152),
Donkey IgG anti-Rat IgG (H+L)-Cy3 (Cat. No. 712-165-153).
FACS:
monoclonal rat anti-CD3ε–APC–eFluor 780 (Clone 17A2, Cat. No. 47-0032-82, eBioscience; dilution 1:25) is validated by the vendor using immune fluorescence and flow cytometry analysis of mouse splenocytes (https://www.thermofisher.com/antibody/product/CD3-Antibody-clone-17A2-Monoclonal/47-0032-82 and https://www.thermofisher.com/antibody/product/CD3-Antibody-clone-17A2-Monoclonal/14-0032-82).
monoclonal rat anti-CD3ε–PE (Clone 145-2C11, Cat. No. 553063, BD Pharmingen; dilution 1:25) is validated by the vendor using flow cytometry analysis of mouse splenocytes (https://www.bdbiosciences.com/content/bdb/paths/generate-tds-document.us.553063.pdf).
monoclonal rat anti-CD3ε–BV510 (Clone 17-A2, Cat. No. 100233, BioLegend; dilution 1:100) is validated by the vendor using flow cytometry analysis of mouse splenocytes with a series of titration dilutions for each lot (https://www.biolegend.com/en-us/search-results/brilliant-violet-510-anti-mouse-cd3-antibody-7990).
monoclonal rat anti-CD16/32–PE–Cy7 (Clone 93, Cat. No. 25-0161-82, eBioscience; dilution 1:400) is validated by the vendor using flow cytometry analysis of mouse splenocytes (https://www.thermofisher.com/antibody/product/CD16-CD32-Antibody-clone-93-Monoclonal/25-0161-82).
monoclonal rat anti-CD34–eFluor 660 (Clone RAM34, Cat. No. 50-0341, eBioscience; dilution 1:400) is validated by the vendor using flow cytometry analysis of lineage negative (https://www.thermofisher.com/antibody/product/Mouse-Hematopoietic-Lineage-Antibody-Cocktail/88-7772-72) mouse bone marrow cells (https://www.thermofisher.com/antibody/product/CD34-Antibody-clone-RAM34-Monoclonal/50-0341-82).
monoclonal rat anti-CD48-AF700 (Clone HM48-1, Cat. No. 103426, BioLegend; dilution 1:400) is validated by the vendor using flow cytometry analysis of mouse splenocytes with a series of titration dilutions for each lot (https://www.biolegend.com/en-us/search-results/alexa-Fluor-700-anti-mouse-cd48-antibody-6670).
monoclonal rat anti-Sca1–PerCP–Cy5.5 (Clone D7, Cat. No. 45-5981, eBioscience; dilution 1:400) is validated by the vendor using flow cytometry analysis of lineage negative (https://www.thermofisher.com/antibody/product/Mouse-Hematopoietic-Lineage-Antibody-Cocktail/88-7772-72) mouse bone marrow cells (https://www.thermofisher.com/antibody/product/Ly-6A-E-Sca-1-Antibody-clone-D7-Monoclonal/45-5981-82).
monoclonal rat anti-CD19–BV421 (Clone 6D5, Cat. No. 115537, BioLegend; dilution 1:400), monoclonal rat anti-CD19–BV605 (Clone 6D5, Cat. No. 115540, BioLegend; dilution 1:800) and monoclonal rat anti-CD19–PerCPCy5.5 (Clone 6D5, Cat. No. 115534, BioLegend; dilution 1:100) are validated by the vendor using flow cytometry analysis of mouse splenocytes with a series of titration dilutions for each lot (https://www.biolegend.com/en-us/search-results/brilliant-violet-421-anti-mouse-cd19-antibody-7160, https://www.biolegend.com/en-us/products/brilliant-violet-605-anti-mouse-cd19-antibody-7645?GroupID=BLG10556 and https://www.biolegend.com/en-us/products/percp-cyanine5-5-anti-mouse-cd19-antibody-4261?GroupID=BLG10556).
monoclonal rat anti-CD117–BV711 (Clone 2B8, Cat. No. 105835, BioLegend; dilution 1:800) is validated by the vendor using flow cytometry analysis of mouse splenocytes with a series of titration dilutions for each lot (https://www.biolegend.com/en-us/products/brilliant-violet-711-anti-mouse-cd117-c-kit-antibody-12049)
monoclonal rat anti-CD150–BV605 (Clone TC15–12F12.2, Cat. No. 115927, BioLegend; dilution 1:100) is validated by the vendor using flow cytometry analysis of mouse splenocytes with a series of titration dilutions for each lot (https://www.biolegend.com/en-gb/search-results/brilliant-violet-605-anti-mouse-cd150-slam-antibody-7871).
monoclonal rat anti-Gr1-APC (Clone RB6-8C5, Cat. No. 553129, BD Pharmingen; dilution 1:400) and monoclonal rat anti-Gr1-PE (Clone RB6-8C5, Cat. No. 553128, BD Pharmingen; dilution 1:400) are validated by the vendor using flow cytometry analysis of mouse splenocytes (https://www.bdbiosciences.com/en-us/products/reagents/flow-cytometry-reagents/research-reagents/single-color-antibodies-ruo/apc-rat-anti-mouse-ly-6g-and-ly-6c.553129 and https://www.bdbiosciences.com/en-us/products/reagents/flow-cytometry-reagents/research-reagents/single-color-antibodies-ruo/pe-rat-anti-mouse-ly-6g-and-ly-6c.553128).
monoclonal rat anti-CD45-PE-Cy7 (Clone 30-F11, Cat. No. 25-0451-82, eBioscience; dilution 1:400) is validated by the vendor using flow cytometry analysis of mouse splenocytes (https://www.thermofisher.com/antibody/product/CD45-Antibody-clone-30-F11-

Monoclonal/25-0451-82).
monoclonal rat anti-CD45-APC (Clone 30-F11, Cat. No. 559864, BD Pharmingen; dilution 1:50) is validated by the vendor using flow cytometry analysis of mouse splenocytes (https://www.bdbiosciences.com/en-us/products/reagents/flow-cytometry-reagents/research-reagents/single-color-antibodies-ruo/apc-rat-anti-mouse-cd45.559864).
monoclonal rat anti-CD11b-PerCP-Cy5.5 (Clone M1/70, Cat. No. 45-0112-82, eBioscience; dilution 1:400) and monoclonal rat anti-CD11b-PE (Clone M1/70, Cat. No. 12-0112-82, eBioscience; dilution 1:800) are validated by the vendor using flow cytometry analysis of mouse bone marrow cells (https://www.thermofisher.com/antibody/product/CD11b-Antibody-clone-M1-70-Monoclonal/45-0112-82 and https://www.thermofisher.com/antibody/product/CD11b-Antibody-clone-M1-70-Monoclonal/12-0112-82).
monoclonal rat anti-CD44–BV605 (Clone IM7, Cat. No. 103047, BioLegend; dilution 1:400) is validated by the vendor using flow cytometry analysis of mouse splenocytes with a series of titration dilutions for each lot (https://www.biolegend.com/en-us/products/brilliant-violet-605-anti-mouse-human-cd44-antibody-8807?GroupID=BLG10425).
monoclonal rat anti-Ter119–PE (Clone Ter119, Cat. No. 553673, BD Pharmingen; dilution 1:50) is validated by the vendor using flow cytometry analysis of mouse fetal liver (https://www.bdbiosciences.com/en-us/products/reagents/flow-cytometry-reagents/research-reagents/single-color-antibodies-ruo/pe-rat-anti-mouse-ter-119-erythroid-cells.553673).
monoclonal rat anti-Ter119–APC-Cy7 (Clone Ter119, Cat. No. 116223, BioLegend; dilution 1:100) is validated by the vendor using flow cytometry analysis of mouse bone marrow cells with a series of titration dilutions for each lot (https://www.biolegend.com/en-us/products/apc-cyanine7-anti-mouse-ter-119-erythroid-cells-antibody-3905?GroupID=ImportedGROUP1).
monoclonal rat anti-CD71–PE-Cy7 (Clone RI7217, Cat. No. 113811, BioLegend; dilution 1:100) and monoclonal rat anti-CD71–PE (Clone RI7217, Cat. No. 113808, BioLegend; dilution 1:100) are validated by the vendor using flow cytometry analysis of mouse bone marrow cells with a series of titration dilutions for each lot (https://www.biolegend.com/en-us/products/pe-cyanine7-anti-mouse-cd71-antibody-6185?GroupID=BLG4775 and https://www.biolegend.com/en-us/products/pe-anti-mouse-cd71-antibody-1631?GroupID=BLG4775).
monoclonal rat anti-CD45.1-BV785 (Clone A20, Cat. No. 110743, BioLegend; dilution 1:100) is validated by the vendor using flow cytometry analysis of mouse splenocytes with a series of titration dilutions for each lot (https://www.biolegend.com/en-us/products/brilliant-violet-785-anti-mouse-cd45-1-antibody-9654).
monoclonal rat anti-CD45.2-BV711 (Clone 104, Cat. No. 563685, eBioscience; dilution 1:400) is validated by the vendor using flow cytometry analysis of mouse splenocytes (https://www.bdbiosciences.com/en-us/products/reagents/flow-cytometry-reagents/research-reagents/single-color-antibodies-ruo/bv711-mouse-anti-mouse-cd45-2.563685).
monoclonal rat anti-CD45R-BV421 (Clone RA3-6B2, Cat. No. 103251, BioLegend; 1:100) is validated by the vendor using flow cytometry analysis of mouse splenocytes with a series of titration dilutions for each lot (https://www.biolegend.com/en-us/products/brilliant-violet-421-anti-mouse-human-cd45r-b220-antibody-7158).
monoclonal rat anti-IgM-FITC (Clone 121-15F9, Cat. No. 11-5890-85, eBioscience; dilution 1:100) is validated by the vendor using flow cytometry analysis of mouse bone marrow cells (https://www.thermofisher.com/antibody/product/IgM-Antibody-clone-eB121-15F9-Monoclonal/11-5890-82).
monoclonal rat anti-MHCII-APC (Clone M5/114.15.2, Cat. No. 17-5321-81, eBioscience; dilution 1:400) is validated by the vendor using flow cytometry analysis of mouse splenocytes (https://www.thermofisher.com/antibody/product/MHC-Class-II-I-A-I-E-Antibody-clone-M5-114-15-2-Monoclonal/17-5321-82).
monoclonal rat anti-Ly6C-APC-Cy7 (Clone AL-21, Cat. No. 560596, BD Pharmingen; dilution 1:100) is validated by the vendor using flow cytometry analysis of mouse splenocytes (https://www.bdbiosciences.com/en-us/products/reagents/flow-cytometry-reagents/research-reagents/single-color-antibodies-ruo/apc-cy-7-rat-anti-mouse-ly-6c.560596) monoclonal rat anti-Ly6G-PerCP-Cy5.5 (Clone 1A8, Cat. No. 560602, BD Pharmingen; dilution 1:100) is validated by the vendor using flow cytometry analysis of mouse splenocytes (https://www.bdbiosciences.com/en-us/products/reagents/flow-cytometry-reagents/research-reagents/single-color-antibodies-ruo/percp-cy-5-5-rat-anti-mouse-ly-6g.560602).
monoclonal rat anti-CD115-BV605 (Clone AFS98, Cat. No. 135517, BioLegend; dilution 1:400) is validated by the vendor using flow cytometry analysis of thioglycolate-elicited mouse peritoneal macrophages with a series of titration dilutions for each lot (https://www.biolegend.com/en-gb/products/brilliant-violet-605-anti-mouse-cd115-csf-1r-antibody-9013).
monoclonal rat anti-EMCN-PE (Clone V.7C7, Cat. No. 12-5851-82, eBioscience) is validated by the vendor using flow cytometry analysis of bEnd.3 cells (https://www.thermofisher.com/antibody/product/Endomucin-Antibody-clone-eBioV-7C7-V-7C7-Monoclonal/12-5851-82).
FACS (Lineage cocktail)
monoclonal rat anti-CD3ε-BV421 (Clone 17A2, Cat. No. 100228, BioLegend; dilution 1:200), monoclonal rat anti-CD4-BV421 (Clone GK1.5, Cat. No. 100443, BioLegend; dilution 1:800), monoclonal rat anti-CD8-BV421 (Clone 53-6.7, Cat. No. 100753, BioLegend; dilution 1:800), monoclonal rat anti-CD11b-BV421 (Clone M1/70, Cat. No. 101251, BioLegend; dilution 1:800), monoclonal rat anti-CD19-BV421 (Clone 6D5, Cat. No. 115549, BioLegend; dilution 1:400), monoclonal rat anti-Gr-1-BV421 (Clone RB6-8C5, Cat. No. 108445, BioLegend; dilution 1:800) and monoclonal rat anti-Ter119-BV421 (Clone Ter119, Cat. No. 116234, BioLegend; dilution 1:200) are validated by the vendor using flow cytometry analysis of mouse splenocytes, bone marrow cells or blood cells with a series of titration dilutions for each lot (https://www.biolegend.com/en-gb/products/brilliant-violet-421-anti-mouse-cd3-antibody-7326, and https://www.biolegend.com/en-us/search-results/brilliant-violet-421-anti-mouse-cd4-antibody-7142, and https://www.biolegend.com/en-us/products/brilliant-violet-421-anti-mouse-cd8a-antibody-7138?GroupID=BLG6765, and https://www.biolegend.com/en-us/search-results/brilliant-violet-421-anti-mouse-human-cd11b-antibody-7163, and https://www.biolegend.com/en-us/search-results/brilliant-violet-421-anti-mouse-cd19-antibody-7160, and https://www.biolegend.com/en-us/products/brilliant-violet-421-anti-mouse-ly-6g-ly-6c-gr-1-antibody-7201, and https://www.biolegend.com/en-us/products/brilliant-violet-421-anti-mouse-ter-119-erythroid-cells-antibody-7259?GroupID=ImportedGROUP1).

Eukaryotic cell lines

Policy information about cell lines

Cell line source(s)	Human (BEL-A) (https://doi.org/10.1038/ncomms14750)
Authentication	Cell line was not authenticated

Mycoplasma contamination	We have not performed any testing for mycoplasma contamination.
Commonly misidentified lines (See ICLAC register)	Cell line is not on the list of commonly misidentified lines

Animals and other organisms

Policy information about studies involving animals; ARRIVE guidelines recommended for reporting animal research

Laboratory animals	All animals were housed under specific pathogen-free conditions in an animal facility (Heidelberg University) at a 12 h/12 h day/night cycle in individually ventilated plastic cages (Green Line, Tecniplast, Buguggiate,Italy) with adjusted air temperature (21°C) and 50% relative humidity. All mice were fed ad libitum with a standard rodent diet (V1534-000, Ssniff, Soest, Germany) and free access to water. Female and male animals aged 2-5 months were used in this study. Female and male reporter animals were analyzed at the age of embryonic day (E)17.5 and older.
Wild animals	No wild animals were used in this study.
Field-collected samples	No field collected samples were used in this study.
Ethics oversight	Animal experiments were performed in accordance with Federal Animal Regulations and were institutionally approved by the district government Karlsruhe and performed under institutional guidelines.

Note that full information on the approval of the study protocol must also be provided in the manuscript.

Flow Cytometry

Plots

Confirm that:

☒ The axis labels state the marker and fluorochrome used (e.g. CD4-FITC).

☒ The axis scales are clearly visible. Include numbers along axes only for bottom left plot of group (a 'group' is an analysis of identical markers).

☒ All plots are contour plots with outliers or pseudocolor plots.

☒ A numerical value for number of cells or percentage (with statistics) is provided.

Methodology

Sample preparation	Hematological blood counts were done from EDTA whole blood samples with automated blood analysers (Sysmex XN-9000) using protocols accredited according to norm ISO 15189 (German Accreditation Body DAkkS). Bone marrow (BM) cells were released from the bones by flushing murine tibia and fibula bones with FACS buffer (5% FCS in PBS), and cells were harvested from the flow-through of a 70-µm filter (BD Falcon). Spleens were dissociated directly by passing the cells through a 40-µm filter. Livers were perfused via the portal vein with ice cold PBS, dissected, mechanically minced, digested at 38°C in collagenase/GBSS (G9779, Sigma-Aldrich) and filtered through a 250 µm filter (BD-Falcon). 30 µL of peripheral blood was collected using EDTA-containing microtubes (Sarstedt), then suspended in PBS; the erythrocytes cells were lysed with RBC Lysis Buffer (10X, BD Pharm Lyse) by incubating them for 15 min at room temperature. Cell suspensions (bone marrow, spleen, liver, peripheral blood) were incubated for 30 min with 500 µg/ml whole mouse IgG (Jackson ImmunoResearch Laboratories) for blocking Fc receptors and stained for 30 min with titrated concentrations of fluorescent dye-labeled antibodies in FACS buffer (5% FCS in PBS) on ice. Dead cells were stained with either Sytox Blue (Invitrogen) or Fixable viability stain 780 (BD Horizon).
Instrument	Flow cytometry was performed using a BD Biosciences FACSCanto II flow cytometer, a BD Biosciences LSR Fortessa cell analyzer or sorted by BD Bioscience FACSAriaIII. Reticulocytes and immature reticulocyte fraction (IRF) were measured with a Sysmex XN 9000 analyzer according to the manufacturer's protocols.
Software	All data were analyzed using FlowJo Versions 10.6.1 and 10.7.1 (Tree Star Inc.) or FACS Diva software (BD Bioscience).
Cell population abundance	Aliquots of sorted cells were reanalyzed using FACS. >80% of live cells feil within the original gates that were used for sorting this confirming the correct phenotype.
Gating strategy	All cells were gated on FSC-A vs. SSC-A morphology, doublet exclusion and live cells (negative staining for Sytox Blue or Fixable Viability dye). Gates were validated by backgating from target populations. Downstream gating is described for each experiment (Supp. Fig. 8).

☒ Tick this box to confirm that a figure exemplifying the gating strategy is provided in the Supplementary Information.
