# Supplementary material for: Bone marrow sinusoidal endothelium controls terminal erythroid differentiation and reticulocyte maturation
Source: Nat Commun. 2021 Nov 29;12:6963. doi: 10.1038/s41467-021-27161-3 (PMC8630019; doi:10.1038/s41467-021-27161-3)
Supplement: Supplementary file 3 — Description of Additional Supplementary Files [file 41467_2021_27161_MOESM3_ESM.pdf]

Description of Additional Supplementary Files

Supplementary Data 1. Significantly differentially expressed genes (DEGs) in PIII of *Ctnnb1*$^{OE\text{-}SEC}$ mice compared to *Ctnnb1*^{WT} controls.

Genes displayed are those significantly up or down regulated in PIII of *Ctnnb1*$^{OE\text{-}SEC}$ compared to *Ctnnb1*^{WT} controls (FC > |1|). Adjusted p-values were calculated for the differences of means of log2 of expressions values between PIII of *Ctnnb1*$^{OE\text{-}SEC}$ mice and *Ctnnb1*^{WT}. OneWay-ANOVA was performed to identify DEGs.

Supplementary Data 2. Significantly differentially expressed genes (DEGs) in PIV of *Ctnnb1*$^{OE\text{-}SEC}$ mice compared to *Ctnnb1*^{WT} controls.

Genes displayed are those significantly up or down regulated in PIV of *Ctnnb1*$^{OE\text{-}SEC}$ compared to *Ctnnb1*^{WT} controls (FC > |1|). Adjusted p-values were calculated for the differences of means of log2 of expressions values between PIV of *Ctnnb1*$^{OE\text{-}SEC}$ mice and *Ctnnb1*^{WT}. OneWay-ANOVA was performed to identify DEGs.
