# Supplementary material for: Bone marrow sinusoidal endothelium controls terminal erythroid differentiation and reticulocyte maturation
Source: Nat Commun. 2021 Nov 29;12:6963. doi: 10.1038/s41467-021-27161-3 (PMC8630019; doi:10.1038/s41467-021-27161-3)
Supplement: Supplementary file 4 — Supplementary Data 1 [file 41467_2021_27161_MOESM4_ESM.docx]

**Supplementary Data file 1. Significantly differentially expressed genes (DEGs) in PIII of *Ctnnb1^OE-SEC^* mice compared to *Ctnnb1^WT^* controls.**

Genes displayed are those significantly up or down regulated in PIII of *Ctnnb1^OE-SEC^* compared to *Ctnnb1^WT^* controls (FC > |1|). Adjusted p-values were calculated for the differences of means of log2 of expressions values between PIII of *Ctnnb1^OE-SEC^* mice and *Ctnnb1^WT^*. OneWay-ANOVA was performed to identify DEGs.

| ***Gene Symbol*** | **Gene Title** | **Fold change *Ctnnb1^OE-SEC^* > WT** | **Adjusted p-Value for Diff of experiment = *Ctnnb1^OE-SEC^* - WT** |
| --- | --- | --- | --- |
| *Nupr1* | nuclear protein transcription regulator 1 | 5.104394531 | 1.00111E-05 |
| *Trib3* | tribbles pseudokinase 3 | 4.909833984 | 2.42669E-05 |
| *Lrrc8b* | leucine rich repeat containing 8 family, member B | 4.480117188 | 1.17673E-08 |
| *Pstpip2* | proline-serine-threonine phosphatase-interacting protein 2 | 4.428251953 | 1.20054E-05 |
| *Leprotl1* | leptin receptor overlapping transcript-like 1 | 4.296757813 | 0.000195751 |
| *Eprs* | glutamyl-prolyl-tRNA synthetase | 4.246113281 | 1.62151E-05 |
| *Ppm1e* | protein phosphatase 1E (PP2C domain containing) | 3.705478516 | 3.01507E-05 |
| *Tspan14* | tetraspanin 14 | 3.492646484 | 2.25022E-05 |
| *Tubb3* | tubulin, beta 3 class III | 3.475615234 | 8.75761E-05 |
| *Cish* | cytokine inducible SH2-containing protein | 3.244511719 | 5.52009E-05 |
| *Spata1* | spermatogenesis associated 1 | 3.235683594 | 0.0002731 |
| *Gss* | glutathione synthetase | 3.136757813 | 0.001754151 |
| *Atp1b2* | ATPase, Na+/K+ transporting, beta 2 polypeptide | 2.9240625 | 0.000388207 |
| *Clic1* | chloride intracellular channel 1 | 2.905175781 | 4.20905E-05 |
| *Ccdc47* | coiled-coil domain containing 47 | 2.828603516 | 6.66777E-05 |
| *Aimp1* | aminoacyl tRNA synthetase complex-interacting multifunctional protein 1 | 2.824111328 | 1.20054E-05 |
| *Plek2* | pleckstrin 2 | 2.803691406 | 0.000362408 |
| *Ifngr2* | interferon gamma receptor 2 | 2.775761719 | 0.002365907 |
| *Trim15* | tripartite motif-containing 15 | 2.772958984 | 0.000175471 |
| *Dstn* | destrin | 2.711367188 | 0.005868359 |
| *Ddit3* | DNA-damage inducible transcript 3 | 2.709199219 | 0.000366406 |
| *Cdkn1a* | cyclin-dependent kinase inhibitor 1A (P21) | 2.685917969 | 0.001045764 |
| *Gm8995* | predicted gene 8995 | 2.638496094 | 0.001861525 |
| *2410006H16Rik* | RIKEN cDNA 2410006H16 gene | 2.610979938 | 0.000217415 |
| *Soat2* | sterol O-acyltransferase 2 | 2.550517578 | 0.000427135 |
| *Gramd4* | GRAM domain containing 4 | 2.541230469 | 4.26296E-05 |
| *Eef1a1* | eukaryotic translation elongation factor 1 alpha 1 | 2.528974609 | 0.001431705 |
| *Atf6* | activating transcription factor 6 | 2.482519531 | 1.31652E-05 |
| *Mt2* | metallothionein 2 | 2.446083984 | 0.001140121 |
| *Rpl14* | ribosomal protein L14 | 2.441796875 | 5.16966E-05 |
| *Prdx1* | peroxiredoxin 1 | 2.429658203 | 0.016307641 |
| *Sdf2* | stromal cell derived factor 2 | 2.401425781 | 0.000223289 |
| *Ankrd36* | ankyrin repeat domain 36 | 2.399072266 | 0.000237806 |
| *Gm4540* | predicted gene 4540 | 2.391591797 | 0.000953764 |
| *Reep6* | receptor accessory protein 6 | 2.384472656 | 4.57864E-05 |
| *Prpf6* | pre-mRNA splicing factor 6 | 2.363632813 | 1.55241E-06 |
| *Psmd3* | proteasome (prosome, macropain) 26S subunit, non-ATPase, 3 | 2.362714844 | 0.000238873 |
| *Akap9* | A kinase (PRKA) anchor protein (yotiao) 9 | 2.340019531 | 0.00022804 |
| *Gm3150* | guanine nucleotide binding protein (G protein), gamma 5 pseudogene | 2.330742188 | 0.003173274 |
| *1700097N02Rik* | RIKEN cDNA 1700097N02 gene | 2.32125 | 0.003685716 |
| *Snhg1* | small nucleolar RNA host gene 1 | 2.309316277 | 0.00013286 |
| *Nup62* | nucleoporin 62 | 2.2959375 | 9.82684E-05 |
| *Tsc22d2* | TSC22 domain family, member 2 | 2.274472656 | 4.62829E-05 |
| *Ccdc66* | coiled-coil domain containing 66 | 2.245830078 | 0.000245584 |
| *Agfg1* | ArfGAP with FG repeats 1 | 2.239238281 | 6.29021E-05 |
| *Pin4* | protein (peptidyl-prolyl cis/trans isomerase) NIMA-interacting, 4 (parvulin) | 2.23565918 | 0.000324212 |
| *Lgals8* | lectin, galactose binding, soluble 8 | 2.197392578 | 0.000824832 |
| *Tbpl1* | TATA box binding protein-like 1 | 2.189091797 | 0.000577698 |
| *Rab26os* | RAB26, member RAS oncogene family, opposite strand | 2.159619141 | 0.002710398 |
| *Kmt5c* | lysine methyltransferase 5C | 2.158300781 | 0.000790565 |
| *Etfdh* | electron transferring flavoprotein, dehydrogenase | 2.151845703 | 0.00034142 |
| *Eif2a* | eukaryotic translation initiation factor 2A | 2.114736328 | 0.000437038 |
| *Brd8* | bromodomain containing 8 | 2.110683594 | 0.001013054 |
| *2610524H06Rik* | RIKEN cDNA 2610524H06 gene | 2.101210938 | 0.018274105 |
| *Eif3l* | eukaryotic translation initiation factor 3, subunit L | 2.083256836 | 0.000274499 |
| *Mier3* | MIER family member 3 | 2.080546875 | 5.34176E-05 |
| *Uxt* | ubiquitously expressed transcript | 2.053007813 | 0.003556512 |
| *Il4ra* | interleukin 4 receptor, alpha | 2.045458984 | 0.024146769 |
| *Cecr2* | CECR2, histone acetyl-lysine reader | 2.037138672 | 0.011199183 |
| *Pycr1* | pyrroline-5-carboxylate reductase 1 | 2.028544922 | 0.00709901 |
| *Rpl29* | ribosomal protein L29 | 2.02765625 | 0.000160434 |
| *Popdc2* | popeye domain containing 2 | 2.026435547 | 0.00455158 |
| *Gm15429* | anterior pharynx defective 1a homolog pseudogene | 2.023496094 | 0.001894715 |
| *Uhrf1bp1l* | UHRF1 (ICBP90) binding protein 1-like | 2.014755859 | 0.002254148 |
| *Hprt* | hypoxanthine guanine phosphoribosyl transferase | 1.996884766 | 0.004087742 |
| *Ndrg3* | N-myc downstream regulated gene 3 | 1.995195313 | 1.20054E-05 |
| *Nsfl1c* | NSFL1 (p97) cofactor (p47) | 1.989697266 | 0.000154121 |
| *Sesn2* | sestrin 2 | 1.988476563 | 0.001295714 |
| *Pi4k2a* | phosphatidylinositol 4-kinase type 2 alpha | 1.959287109 | 0.008637644 |
| *Rps11* | ribosomal protein S11 | 1.953730469 | 0.005110328 |
| *Mia3* | melanoma inhibitory activity 3 | 1.948076172 | 0.000216434 |
| *Aaed1* | AhpC/TSA antioxidant enzyme domain containing 1 | 1.931894531 | 0.005346095 |
| *Psmc6* | proteasome (prosome, macropain) 26S subunit, ATPase, 6 | 1.929648438 | 1.00111E-05 |
| *Aoc3* | amine oxidase, copper containing 3 | 1.918837891 | 0.020575808 |
| *Cyb5r1* | cytochrome b5 reductase 1 | 1.904775391 | 0.000930973 |
| *Arl6ip5* | ADP-ribosylation factor-like 6 interacting protein 5 | 1.887558594 | 6.86925E-05 |
| *Tmed9* | transmembrane p24 trafficking protein 9 | 1.886523438 | 0.000299863 |
| *Rpl36al* | ribosomal protein L36A-like | 1.880458984 | 0.000914367 |
| *Braf* | Braf transforming gene | 1.869785156 | 0.002172244 |
| *Mob3a* | MOB kinase activator 3A | 1.864462891 | 0.02255542 |
| *Eif3e* | eukaryotic translation initiation factor 3, subunit E | 1.858417969 | 0.001604327 |
| *Ss18l2* | SS18, nBAF chromatin remodeling complex subunit like 2 | 1.856445313 | 0.001434573 |
| *Itgb1* | integrin beta 1 (fibronectin receptor beta) | 1.84984375 | 0.00051093 |
| *Plaa* | phospholipase A2, activating protein | 1.847304688 | 0.00120963 |
| *Tnfrsf21* | tumor necrosis factor receptor superfamily, member 21 | 1.835976563 | 0.003106095 |
| *Cldnd1* | claudin domain containing 1 | 1.833691406 | 0.000449001 |
| *Rpl27a* | ribosomal protein L27A | 1.831464844 | 0.002889913 |
| *Msl3* | MSL complex subunit 3 | 1.83050293 | 0.006058734 |
| *Cacna1c* | calcium channel, voltage-dependent, L type, alpha 1C subunit | 1.829897461 | 0.000716913 |
| *Ern1* | endoplasmic reticulum (ER) to nucleus signalling 1 | 1.822402344 | 0.003962474 |
| *Ccdc28a* | coiled-coil domain containing 28A | 1.814121094 | 0.006097886 |
| *Cdkn2aip* | CDKN2A interacting protein | 1.809648438 | 0.001575053 |
| *Dapp1* | dual adaptor for phosphotyrosine and 3-phosphoinositides 1 | 1.806650391 | 6.88489E-05 |
| *Psmc3* | proteasome (prosome, macropain) 26S subunit, ATPase 3 | 1.802617188 | 1.27754E-05 |
| *Mmel1* | membrane metallo-endopeptidase-like 1 | 1.801953125 | 0.000186454 |
| *Cebpg* | CCAAT/enhancer binding protein (C/EBP), gamma | 1.7925 | 0.026748437 |
| *Tmem259* | transmembrane protein 259 | 1.786660156 | 1.12937E-05 |
| *Kdm6b* | KDM1 lysine (K)-specific demethylase 6B | 1.785263672 | 0.000230994 |
| *Raly* | hnRNP-associated with lethal yellow | 1.783378906 | 0.000175471 |
| *Tusc3* | tumor suppressor candidate 3 | 1.770166016 | 0.000804092 |
| *Sdsl* | serine dehydratase-like | 1.766923828 | 0.001040208 |
| *Nup88* | nucleoporin 88 | 1.766777344 | 0.004620784 |
| *Upf1* | UPF1 regulator of nonsense transcripts homolog (yeast) | 1.762509766 | 0.000468244 |
| *Slirp* | SRA stem-loop interacting RNA binding protein | 1.755561523 | 0.000753896 |
| *Bcl10* | B cell leuk  emia/lymphoma 10 | 1.752285156 | 0.000495659 |
| *Zfas1* | zinc finger, NFX1-type containing 1, antisense RNA 1 | 1.746582031 | 0.000352999 |
| *Chm* | choroidermia (RAB escort protein 1) | 1.714511719 | 0.001585701 |
| *Gm10260* | ribosomal protein S18 pseudogene | 1.713828125 | 0.007723607 |
| *Surf1* | surfeit gene 1 | 1.712124023 | 0.001331306 |
| *Hspa9* | heat shock protein 9 | 1.704082031 | 0.000243933 |
| *Nfyb* | nuclear transcription factor-Y beta | 1.703095703 | 0.000710651 |
| *Iah1* | isoamyl acetate-hydrolyzing esterase 1 homolog | 1.699755859 | 0.000469946 |
| *Fdps* | farnesyl diphosphate synthetase | 1.696953125 | 0.00539246 |
| *Stt3b* | STT3, subunit of the oligosaccharyltransferase complex, homolog B (S. cerevisiae) | 1.695966797 | 0.001502151 |
| *Psmd13* | proteasome (prosome, macropain) 26S subunit, non-ATPase, 13 | 1.690664047 | 4.68105E-05 |
| *Ilk* | integrin linked kinase | 1.683261719 | 0.009343454 |
| *Derl1* | Der1-like domain family, member 1 | 1.680566406 | 0.001048933 |
| *Tmem128* | transmembrane protein 128 | 1.676982422 | 0.00051323 |
| *Maip1* | matrix AAA peptidase interacting protein 1 | 1.670249023 | 0.004528036 |
| *Eif3c* | eukaryotic translation initiation factor 3, subunit C | 1.667382813 | 0.002998063 |
| *Gm17352* | ribosomal protein S18 pseudogene | 1.662880859 | 0.002638189 |
| *Zbed6* | zinc finger, BED type containing 6 | 1.660976563 | 0.01305313 |
| *Cfap36* | cilia and flagella associated protein 36 | 1.658867188 | 0.023914448 |
| *Hist1h2ac* | histone cluster 1, H2ac | 1.658847656 | 0.01656139 |
| *Haus3* | HAUS augmin-like complex, subunit 3 | 1.651484375 | 7.0101E-05 |
| *Ubxn4* | UBX domain protein 4 | 1.648535156 | 0.000452027 |
| *Aars* | alanyl-tRNA synthetase | 1.647421875 | 0.005073424 |
| *Slc31a1* | solute carrier family 31, member 1 | 1.633759766 | 0.000292577 |
| *Got1* | glutamic-oxaloacetic transaminase 1, soluble | 1.631538086 | 0.004141152 |
| *Ndufc2* | NADH dehydrogenase (ubiquinone) 1, subcomplex unknown, 2 | 1.626972656 | 0.023503555 |
| *Stk40* | serine/threonine kinase 40 | 1.6225 | 0.005220341 |
| *Ube2a* | ubiquitin-conjugating enzyme E2A | 1.622324219 | 0.000113293 |
| *Bnip2* | BCL2/adenovirus E1B interacting protein 2 | 1.621513672 | 0.01228297 |
| *Fam3a* | family with sequence similarity 3, member A | 1.619833984 | 0.007206414 |
| *Lars* | leucyl-tRNA synthetase | 1.619472656 | 0.011569043 |
| *Egln2* | egl-9 family hypoxia-inducible factor 2 | 1.619140625 | 0.008593068 |
| *Etfrf1* | electron transfer flavoprotein regulatory factor 1 | 1.618818359 | 0.000257558 |
| *Snw1* | SNW domain containing 1 | 1.613632813 | 0.001388 |
| *Tapt1* | transmembrane anterior posterior transformation 1 | 1.607685547 | 0.002617673 |
| *Adamtsl5* | ADAMTS-like 5 | 1.606142512 | 0.00184101 |
| *Tspo* | translocator protein | 1.603945313 | 8.77132E-05 |
| *Ssbp2* | single-stranded DNA binding protein 2 | 1.598828125 | 0.003601113 |
| *Plxnc1* | plexin C1 | 1.598320313 | 0.02830541 |
| *Arhgap25* | Rho GTPase activating protein 25 | 1.597519531 | 0.00127812 |
| *Gdf15* | growth differentiation factor 15 | 1.595976563 | 0.012957228 |
| *Rpl36-ps3* | ribosomal protein L36, pseudogene 3 | 1.59387207 | 0.027287448 |
| *Timm17a* | translocase of inner mitochondrial membrane 17a | 1.585273438 | 0.000533083 |
| *Acadvl* | acyl-Coenzyme A dehydrogenase, very long chain | 1.584101563 | 0.000808415 |
| *Capza2* | capping protein (actin filament) muscle Z-line, alpha 2 | 1.571425781 | 0.011572104 |
| *Psmc2* | proteasome (prosome, macropain) 26S subunit, ATPase 2 | 1.565742275 | 5.64346E-06 |
| *Psmc4* | proteasome (prosome, macropain) 26S subunit, ATPase, 4 | 1.565556641 | 0.001037211 |
| *Gmpr* | guanosine monophosphate reductase | 1.564658203 | 0.035302279 |
| *Susd2* | sushi domain containing 2 | 1.557822266 | 0.001163789 |
| *Snora68* | small nucleolar RNA, H/ACA box 68 | 1.555732422 | 0.004739088 |
| *Fxr2* | fragile X mental retardation, autosomal homolog 2 | 1.554667969 | 0.001906063 |
| *Gm14586* | predicted gene 14586 | 1.548681641 | 0.017485218 |
| *Rmdn3* | regulator of microtubule dynamics 3 | 1.548134766 | 0.000828621 |
| *Tle4* | transducin-like enhancer of split 4 | 1.542382813 | 0.029824071 |
| *Noct* | nocturnin | 1.541972656 | 0.001406431 |
| *Abcg2* | ATP binding cassette subfamily G member 2 (Junior blood group) | 1.529960938 | 0.000449448 |
| *Shmt2* | serine hydroxymethyltransferase 2 (mitochondrial) | 1.526894531 | 0.040164054 |
| *Supt20* | suppressor of Ty 20 | 1.525375977 | 0.01828053 |
| *Cct3* | chaperonin containing Tcp1, subunit 3 (gamma) | 1.523388672 | 0.002032935 |
| *Acadsb* | acyl-Coenzyme A dehydrogenase, short/branched chain | 1.520380859 | 0.005471295 |
| *Dnajc14* | DnaJ heat shock protein family (Hsp40) member C14 | 1.518720703 | 0.000543088 |
| *Snd1* | staphylococcal nuclease and tudor domain containing 1 | 1.518291016 | 0.000650279 |
| *Xbp1* | X-box binding protein 1 | 1.515898438 | 0.00687634 |
| *Prkar1a* | protein kinase, cAMP dependent regulatory, type I, alpha | 1.512158203 | 0.002252102 |
| *Mrpl34* | mitochondrial ribosomal protein L34 | 1.511572266 | 0.002402385 |
| *Pomp* | proteasome maturation protein | 1.510214844 | 0.00249578 |
| *Mt1* | metallothionein 1 | 1.501191406 | 0.000208183 |
| *Fah* | fumarylacetoacetate hydrolase | 1.498017578 | 0.014505309 |
| *Mtmr12* | myotubularin related protein 12 | 1.494091797 | 0.005188212 |
| *M6pr* | mannose-6-phosphate receptor, cation dependent | 1.489414063 | 0.000203642 |
| *Gm26843* | predicted gene, 26843 | 1.474628906 | 0.008386753 |
| *Mtf1* | metal response element binding transcription factor 1 | 1.474335938 | 0.007798658 |
| *Lsm10* | U7 snRNP-specific Sm-like protein LSM10 | 1.474033203 | 0.000429386 |
| *Xpr1* | xenotropic and polytropic retrovirus receptor 1 | 1.47296875 | 0.002178893 |
| *Golim4* | golgi integral membrane protein 4 | 1.469960938 | 0.000926841 |
| *Ilf2* | interleukin enhancer binding factor 2 | 1.464189453 | 0.047703798 |
| *Blmh* | bleomycin hydrolase | 1.462460938 | 0.000764928 |
| *Riok1* | RIO kinase 1 (yeast) | 1.46227539 | 0.006211081 |
| *Cnppd1* | cyclin Pas1/PHO80 domain containing 1 | 1.458984375 | 0.012509568 |
| *Ptdss1* | phosphatidylserine synthase 1 | 1.455859375 | 0.000532572 |
| *Hmgcr* | 3-hydroxy-3-methylglutaryl-Coenzyme A reductase | 1.455429688 | 0.001620495 |
| *Mocs2* | molybdenum cofactor synthesis 2 | 1.454814453 | 0.000929802 |
| *Gm7591* | predicted gene 7591 | 1.454345703 | 0.040007234 |
| *Gm5481* | predicted gene 5481 | 1.449619141 | 0.006781945 |
| *Arfgap2* | ADP-ribosylation factor GTPase activating protein 2 | 1.449394531 | 0.003027366 |
| *Prr13* | proline rich 13 | 1.446621094 | 0.004288219 |
| *Zbtb22* | zinc finger and BTB domain containing 22 | 1.443920898 | 0.01308311 |
| *Atf5* | activating transcription factor 5 | 1.442080078 | 0.004977588 |
| *Cdc37* | cell division cycle 37 | 1.441972656 | 0.000165249 |
| *Tcf25* | transcription factor 25 (basic helix-loop-helix) | 1.439257813 | 0.005306165 |
| *Prpf40a* | pre-mRNA processing factor 40A | 1.438955078 | 0.000193425 |
| *Apool* | apolipoprotein O-like | 1.436142579 | 0.000727747 |
| *Psmb7* | proteasome (prosome, macropain) subunit, beta type 7 | 1.432246094 | 0.000241659 |
| *Ndel1* | nudE neurodevelopment protein 1 like 1 | 1.430634766 | 0.013085025 |
| *Snx1* | sorting nexin 1 | 1.429863281 | 0.003178079 |
| *Psmc5* | protease (prosome, macropain) 26S subunit, ATPase 5 | 1.427207031 | 0.000274185 |
| *Ndufc1* | NADH dehydrogenase (ubiquinone) 1, subcomplex unknown, 1 | 1.42203125 | 0.000380883 |
| *Dctn4* | dynactin 4 | 1.41984375 | 0.001028403 |
| *Sars* | seryl-aminoacyl-tRNA synthetase | 1.412304688 | 0.006313393 |
| *Tbc1d31* | TBC1 domain family, member 31 | 1.408417969 | 0.014737228 |
| *Spcs2-ps* | signal peptidase complex subunit 2, pseudogene | 1.407116159 | 0.00252336 |
| *Gm13680* | ribosomal protein S4, X-linked pseudogene | 1.406328125 | 0.007263373 |
| *Rny1* | RNA, Y1 small cytoplasmic, Ro-associated | 1.403886719 | 0.031981981 |
| *Ddt* | D-dopachrome tautomerase | 1.402460938 | 0.000410739 |
| *Trappc3* | trafficking protein particle complex 3 | 1.399677734 | 0.014489521 |
| *Rpsa* | ribosomal protein SA | 1.398671875 | 0.003536639 |
| *Tmbim4* | transmembrane BAX inhibitor motif containing 4 | 1.395722656 | 0.002763336 |
| *Zcchc11* | zinc finger, CCHC domain containing 11 | 1.392363281 | 0.023190481 |
| *Cisd1* | CDGSH iron sulfur domain 1 | 1.39050293 | 0.002817224 |
| *Epb41l4aos* | erythrocyte membrane protein band 4.1 like 4a, opposite strand | 1.390380859 | 0.021055727 |
| *Mrpl3* | mitochondrial ribosomal protein L3 | 1.389101563 | 0.028433279 |
| *Cnst* | consortin, connexin sorting protein | 1.388134766 | 0.002429815 |
| *Fgd6* | FYVE, RhoGEF and PH domain containing 6 | 1.386943359 | 0.000556367 |
| *Ufd1* | ubiquitin recognition factor in ER-associated degradation 1 | 1.385703125 | 0.000216319 |
| *Lnpk* | lunapark, ER junction formation factor | 1.385283203 | 0.00245121 |
| *Clint1* | clathrin interactor 1 | 1.382675781 | 0.000490247 |
| *4930523C07Rik* | RIKEN cDNA 4930523C07 gene | 1.380976563 | 0.049165878 |
| *Dap3* | death associated protein 3 | 1.379296875 | 0.006161688 |
| *Tmem9b* | TMEM9 domain family, member B | 1.378505859 | 0.013564139 |
| *Hint1* | histidine triad nucleotide binding protein 1 | 1.377539062 | 0.00348105 |
| *Osbpl2* | oxysterol binding protein-like 2 | 1.376044922 | 0.026498424 |
| *Crtc2* | CREB regulated transcription coactivator 2 | 1.369335938 | 0.036262848 |
| *Pex3* | peroxisomal biogenesis factor 3 | 1.369316406 | 0.000955335 |
| *Tmem30a* | transmembrane protein 30A | 1.361019018 | 0.0002394 |
| *NA* | NA | 1.356191406 | 0.034962155 |
| *Vps13c* | vacuolar protein sorting 13C | 1.35121582 | 2.15377E-05 |
| *Naa15* | N(alpha)-acetyltransferase 15, NatA auxiliary subunit | 1.349804688 | 0.001040372 |
| *Gm40346* | predicted gene, 40346 | 1.349174805 | 0.002714845 |
| *Psmd1* | proteasome (prosome, macropain) 26S subunit, non-ATPase, 1 | 1.348300781 | 9.36492E-05 |
| *Ginm1* | glycoprotein integral membrane 1 | 1.347080078 | 0.00606664 |
| *Creb3* | cAMP responsive element binding protein 3 | 1.342387695 | 0.011164349 |
| *Zpr1* | ZPR1 zinc finger | 1.342255859 | 0.001739621 |
| *Gm8394* | proteasome zeta chain | 1.342226563 | 0.000185584 |
| *Kmt2d* | lysine (K)-specific methyltransferase 2D | 1.339482422 | 1.72075E-05 |
| *Eif4g1* | eukaryotic translation initiation factor 4, gamma 1 | 1.337558594 | 0.000926933 |
| *Mindy1* | MINDY lysine 48 deubiquitinase 1 | 1.333671875 | 0.000400139 |
| *Tfpt* | TCF3 (E2A) fusion partner | 1.332421875 | 0.001866706 |
| *Rexo2* | RNA exonuclease 2 | 1.327304688 | 4.62829E-05 |
| *Psmb6* | proteasome (prosome, macropain) subunit, beta type 6 | 1.325507813 | 0.00027249 |
| *Nop53* | NOP53 ribosome biogenesis factor | 1.323125 | 0.001076729 |
| *Gm4609* | glyceraldehyde-3-phosphate dehydrogenase pseudogene | 1.320566406 | 0.02660223 |
| *Psmd14* | proteasome (prosome, macropain) 26S subunit, non-ATPase, 14 | 1.317147991 | 0.002754427 |
| *Lrp6* | low density lipoprotein receptor-related protein 6 | 1.31421875 | 0.0269864 |
| *Tceal9* | transcription elongation factor A like 9 | 1.313681641 | 0.004962288 |
| *Snhg12* | small nucleolar RNA host gene 12 | 1.312026367 | 0.005032466 |
| *Supt5* | suppressor of Ty 5 | 1.311279297 | 1.62151E-05 |
| *March7* | membrane-associated ring finger (C3HC4) 7 | 1.309179688 | 0.019127693 |
| *Sp4* | trans-acting transcription factor 4 | 1.307939453 | 0.00679947 |
| *Gm10709* | ribosomal protein L29 pseudogene | 1.307353516 | 0.001315808 |
| *Prelid3b* | PRELI domain containing 3B | 1.305019531 | 0.001611662 |
| *Psma7* | proteasome (prosome, macropain) subunit, alpha type 7 | 1.30421875 | 0.009514313 |
| *Tmem251* | transmembrane protein 251 | 1.302651367 | 0.001035122 |
| *Cdc42bpa* | CDC42 binding protein kinase alpha | 1.295639649 | 0.002705823 |
| *Igkv12-47* | immunoglobulin kappa variable 12-47 | 1.293862305 | 0.01308311 |
| *Mpc1-ps* | mitochondrial pyruvate carrier 1, pseudogene | 1.293740234 | 0.022493491 |
| *Eif3f* | eukaryotic translation initiation factor 3, subunit F | 1.291234515 | 0.006186764 |
| *Gng5-ps* | G protein subunit gamma 5, pseudogene | 1.289628906 | 0.037877605 |
| *Zfp592* | zinc finger protein 592 | 1.278808594 | 0.001824128 |
| *Tom1l1* | target of myb1-like 1 (chicken) | 1.278144531 | 0.000341245 |
| *Rpain* | RPA interacting protein | 1.277900391 | 0.023590279 |
| *1700094D03Rik* | RIKEN cDNA 1700094D03 gene | 1.276962891 | 0.003313897 |
| *Insig2* | insulin induced gene 2 | 1.272841797 | 0.000286914 |
| *Cul1* | cullin 1 | 1.271455078 | 0.001569687 |
| *Tmub2* | transmembrane and ubiquitin-like domain containing 2 | 1.271035156 | 0.000186403 |
| *Eif2b2* | eukaryotic translation initiation factor 2B, subunit 2 beta | 1.270893555 | 0.040208996 |
| *Dusp11* | dual specificity phosphatase 11 (RNA/RNP complex 1-interacting) | 1.270410156 | 1.79226E-05 |
| *Slc7a11* | solute carrier family 7 (cationic amino acid transporter, y+ system), member 11 | 1.266450195 | 0.042245123 |
| *Uba1* | ubiquitin-like modifier activating enzyme 1 | 1.265898438 | 5.53053E-06 |
| *Psmb5-ps* | proteasome (prosome, macropain) subunit, beta type 5, pseudogene | 1.264804688 | 0.004320566 |
| *Gnl3* | guanine nucleotide binding protein-like 3 (nucleolar) | 1.26375 | 9.50243E-06 |
| *Smg9* | smg-9 homolog, nonsense mediated mRNA decay factor (C. elegans) | 1.263237305 | 0.026748437 |
| *Gm9774* | adhesion regulating molecule 1 pseudogene | 1.260878906 | 0.00096154 |
| *Pycr2* | pyrroline-5-carboxylate reductase family, member 2 | 1.256137695 | 0.038028152 |
| *Churc1* | churchill domain containing 1 | 1.251484375 | 0.000601576 |
| *LOC102632778* | uncharacterized LOC102632778 | 1.250341797 | 0.013419206 |
| *Uck2* | uridine-cytidine kinase 2 | 1.248994141 | 0.00205482 |
| *Ppm1l* | protein phosphatase 1 (formerly 2C)-like | 1.244277344 | 0.017551303 |
| *Immt* | inner membrane protein, mitochondrial | 1.242001953 | 0.004410751 |
| *Spcs1* | signal peptidase complex subunit 1 homolog (S. cerevisiae) | 1.241523438 | 0.000332847 |
| *Grk6* | G protein-coupled receptor kinase 6 | 1.240009766 | 0.007885312 |
| *Smg1* | SMG1 homolog, phosphatidylinositol 3-kinase-related kinase (C. elegans) | 1.234121094 | 0.027193635 |
| *Mbd1* | methyl-CpG binding domain protein 1 | 1.233935547 | 0.008485084 |
| *Ddx52* | DEAD (Asp-Glu-Ala-Asp) box polypeptide 52 | 1.233632813 | 0.005502531 |
| *Copb1* | coatomer protein complex, subunit beta 1 | 1.232353516 | 0.020571542 |
| *Rbm22* | RNA binding motif protein 22 | 1.231044922 | 0.001012769 |
| *Vps51* | VPS51 GARP complex subunit | 1.230654297 | 0.0019414 |
| *Tmem11* | transmembrane protein 11 | 1.229228516 | 0.00157583 |
| *Akap8l* | A kinase (PRKA) anchor protein 8-like | 1.226152344 | 0.000273774 |
| *9330133O14Rik* | RIKEN cDNA 9330133O14 gene | 1.225512695 | 0.010993391 |
| *Gm31872* | predicted gene, 31872 | 1.223251953 | 0.023582326 |
| *Gm12508* | predicted gene 12508 | 1.221572266 | 0.001531694 |
| *C1galt1* | core 1 synthase, glycoprotein-N-acetylgalactosamine 3-beta-galactosyltransferase, 1 | 1.217412109 | 0.003226969 |
| *Ddx49* | DEAD (Asp-Glu-Ala-Asp) box polypeptide 49 | 1.216972656 | 0.005430313 |
| *Tmem214* | transmembrane protein 214 | 1.216435547 | 0.019852784 |
| *Cct4* | chaperonin containing Tcp1, subunit 4 (delta) | 1.214633789 | 0.001388 |
| *Spata13* | spermatogenesis associated 13 | 1.211054688 | 0.002402385 |
| *Cr1l* | complement component (3b/4b) receptor 1-like | 1.209902343 | 0.005899806 |
| *Vps13d* | vacuolar protein sorting 13D | 1.206904297 | 0.00013374 |
| *Map3k7* | mitogen-activated protein kinase kinase kinase 7 | 1.205439453 | 0.024381928 |
| *Acadl* | acyl-Coenzyme A dehydrogenase, long-chain | 1.2053125 | 0.0192108 |
| *Cib1* | calcium and integrin binding 1 (calmyrin) | 1.2025 | 0.016251522 |
| *Imp4* | IMP4, U3 small nucleolar ribonucleoprotein | 1.201621094 | 0.03883191 |
| *1110004F10Rik* | RIKEN cDNA 1110004F10 gene | 1.200810547 | 0.001009456 |
| *Gatc* | glutamyl-tRNA(Gln) amidotransferase, subunit C | 1.200668945 | 0.03380258 |
| *Srp72* | signal recognition particle 72 | 1.195996094 | 0.000123488 |
| *Gm3550* | ribosomal protein L29 pseudogene | 1.195596514 | 0.003063462 |
| *Psma1* | proteasome (prosome, macropain) subunit, alpha type 1 | 1.192910156 | 0.046039225 |
| *Mrpl16* | mitochondrial ribosomal protein L16 | 1.192348633 | 0.003547076 |
| *B4galt1* | UDP-Gal:betaGlcNAc beta 1,4- galactosyltransferase, polypeptide 1 | 1.191416016 | 0.006104592 |
| *Ddx39* | DEAD (Asp-Glu-Ala-Asp) box polypeptide 39 | 1.19140625 | 0.000412659 |
| *Sec24c* | Sec24 related gene family, member C (S. cerevisiae) | 1.19046875 | 0.009976061 |
| *Ndufa9* | NADH dehydrogenase (ubiquinone) 1 alpha subcomplex, 9 | 1.189306641 | 0.001425 |
| *Sugt1* | SGT1, suppressor of G2 allele of SKP1 (S. cerevisiae) | 1.183833008 | 0.043646106 |
| *Polh* | polymerase (DNA directed), eta (RAD 30 related) | 1.180625 | 0.046051125 |
| *Rnh1* | ribonuclease/angiogenin inhibitor 1 | 1.177607422 | 0.03209801 |
| *Crlf3* | cytokine receptor-like factor 3 | 1.176894531 | 4.01086E-05 |
| *Prkag1* | protein kinase, AMP-activated, gamma 1 non-catalytic subunit | 1.174794922 | 0.008236511 |
| *Prrc2b* | proline-rich coiled-coil 2B | 1.172607422 | 0.008120798 |
| *Klf1* | Kruppel-like factor 1 (erythroid) | 1.170976563 | 0.004394406 |
| *Rhbdd1* | rhomboid domain containing 1 | 1.168691406 | 0.040203977 |
| *Eif3h* | eukaryotic translation initiation factor 3, subunit H | 1.166855469 | 0.003801604 |
| *Gm9843* | predicted gene 9843 | 1.162128906 | 0.000932076 |
| *Ndufs2* | NADH dehydrogenase (ubiquinone) Fe-S protein 2 | 1.160761719 | 0.000727747 |
| *Mrpl37* | mitochondrial ribosomal protein L37 | 1.160332031 | 0.008478062 |
| *Srebf2* | sterol regulatory element binding factor 2 | 1.159716797 | 0.018068867 |
| *Gm4285* | predicted gene 4285 | 1.158544922 | 0.018456706 |
| *Clca3a1* | chloride channel accessory 3A1 | 1.156069336 | 0.025709015 |
| *Araf* | Araf proto-oncogene, serine/threonine kinase | 1.155620117 | 0.00786295 |
| *Psmb2* | proteasome (prosome, macropain) subunit, beta type 2 | 1.15515625 | 0.000647886 |
| *Rps14* | ribosomal protein S14 | 1.153330078 | 0.047914437 |
| *Asxl1* | additional sex combs like 1 | 1.152304688 | 0.017018451 |
| *Mysm1* | myb-like, SWIRM and MPN domains 1 | 1.151425781 | 0.019190394 |
| *Fam98a* | family with sequence similarity 98, member A | 1.151118164 | 0.028658838 |
| *Trove2* | TROVE domain family, member 2 | 1.149018555 | 0.011463323 |
| *Ip6k2* | inositol hexaphosphate kinase 2 | 1.148886719 | 0.003089274 |
| *St3gal4* | ST3 beta-galactoside alpha-2,3-sialyltransferase 4 | 1.148417969 | 0.022867752 |
| *Vps29* | VPS29 retromer complex component | 1.148417969 | 0.004402059 |
| *Rpl27-ps1* | ribosomal protein L27, pseudogene 1 | 1.148164063 | 0.02346427 |
| *Snhg8* | small nucleolar RNA host gene 8 | 1.146591797 | 0.008032702 |
| *Ergic2* | ERGIC and golgi 2 | 1.141347656 | 0.00175886 |
| *Snord104* | small nucleolar RNA, C/D box 104 | 1.141220703 | 0.022538396 |
| *Dnpep* | aspartyl aminopeptidase | 1.138422852 | 0.024176208 |
| *Rptor* | regulatory associated protein of MTOR, complex 1 | 1.138076172 | 0.003950693 |
| *Map3k20* | mitogen-activated protein kinase kinase kinase 20 | 1.137939459 | 0.002572279 |
| *Elp5* | elongator acetyltransferase complex subunit 5 | 1.137382813 | 0.005524309 |
| *Zswim7* | zinc finger SWIM-type containing 7 | 1.13734375 | 0.010453346 |
| *Gm13609* | predicted gene 13609 | 1.136806641 | 0.014313728 |
| *Mfap1a* | microfibrillar-associated protein 1A | 1.1359375 | 0.040978448 |
| *Grpel1* | GrpE-like 1, mitochondrial | 1.135898438 | 0.010755144 |
| *Rps19-ps3* | ribosomal protein S19, pseudogene 3 | 1.133774414 | 0.00965554 |
| *Anapc7* | anaphase promoting complex subunit 7 | 1.133193359 | 0.010737003 |
| *Akr1a1* | aldo-keto reductase family 1, member A1 (aldehyde reductase) | 1.13203125 | 0.042461133 |
| *Anapc1* | anaphase promoting complex subunit 1 | 1.131103516 | 0.004486695 |
| *Gm3222* | glyceraldehyde-3-phosphate dehydrogenase pseudogene | 1.13015625 | 0.007845801 |
| *Cct5* | chaperonin containing Tcp1, subunit 5 (epsilon) | 1.129208984 | 0.005508258 |
| *Rnf128* | ring finger protein 128 | 1.128183594 | 0.031491037 |
| *Phf23* | PHD finger protein 23 | 1.127080078 | 0.012785737 |
| *Ldah* | lipid droplet associated hydrolase | 1.124682617 | 0.024681221 |
| *Coq5* | coenzyme Q5 methyltransferase | 1.122016572 | 0.025004247 |
| *Cog1* | component of oligomeric golgi complex 1 | 1.121914063 | 0.004960645 |
| *Osbpl9* | oxysterol binding protein-like 9 | 1.121181641 | 0.009554797 |
| *Eif3d* | eukaryotic translation initiation factor 3, subunit D | 1.118343783 | 0.000947722 |
| *Cdk11b* | cyclin-dependent kinase 11B | 1.117265625 | 0.003164906 |
| *Ap2m1* | adaptor-related protein complex 2, mu 1 subunit | 1.110253906 | 0.029416523 |
| *Dnm2* | dynamin 2 | 1.108300781 | 0.000101565 |
| *Uso1* | USO1 vesicle docking factor | 1.107363281 | 0.006355447 |
| *Eci1* | enoyl-Coenzyme A delta isomerase 1 | 1.106962891 | 0.002094218 |
| *Alad* | aminolevulinate, delta-, dehydratase | 1.106289063 | 0.043180303 |
| *Epg5* | ectopic P-granules autophagy protein 5 homolog (C. elegans) | 1.104667969 | 0.008537865 |
| *Cryzl1* | crystallin, zeta (quinone reductase)-like 1 | 1.104189453 | 0.006030616 |
| *Iqcd* | IQ motif containing D | 1.102763672 | 0.004206315 |
| *Bcap31* | B cell receptor associated protein 31 | 1.102373047 | 0.027837436 |
| *Pa2g4* | proliferation-associated 2G4 | 1.101806641 | 0.03284032 |
| *Gtf2f1* | general transcription factor IIF, polypeptide 1 | 1.101044922 | 0.000439252 |
| *Usp47* | ubiquitin specific peptidase 47 | 1.100830078 | 0.004580978 |
| *Eif5b* | eukaryotic translation initiation factor 5B | 1.099873047 | 0.008623599 |
| *Selenoi* | selenoprotein I | 1.0990625 | 0.048949369 |
| *Gp6* | glycoprotein 6 (platelet) | 1.097851563 | 0.048712613 |
| *Psmb1* | proteasome (prosome, macropain) subunit, beta type 1 | 1.096425781 | 0.000159135 |
| *Sec23b* | SEC23 homolog B, COPII coat complex component | 1.095722656 | 0.013215189 |
| *Pcid2* | PCI domain containing 2 | 1.094516602 | 0.037342825 |
| *Abca4* | ATP-binding cassette, sub-family A (ABC1), member 4 | 1.094301758 | 0.006576181 |
| *Akirin1* | akirin 1 | 1.094130859 | 0.011209295 |
| *Mta2* | metastasis-associated gene family, member 2 | 1.092773438 | 0.007241611 |
| *Pgd* | phosphogluconate dehydrogenase | 1.092138672 | 0.037850343 |
| *Dpf2* | D4, zinc and double PHD fingers family 2 | 1.091894531 | 0.000602992 |
| *Mrpl54* | mitochondrial ribosomal protein L54 | 1.086303711 | 0.040009514 |
| *Vps36* | vacuolar protein sorting 36 | 1.085478516 | 0.028687849 |
| *Ercc1* | excision repair cross-complementing rodent repair deficiency, complementation group 1 | 1.085410156 | 0.006003949 |
| *Prpf39* | pre-mRNA processing factor 39 | 1.081464844 | 0.020933492 |
| *Rbm41* | RNA binding motif protein 41 | 1.080625 | 0.041971315 |
| *Thoc5* | THO complex 5 | 1.078964844 | 0.035155374 |
| *Mgat4b* | mannoside acetylglucosaminyltransferase 4, isoenzyme B | 1.073623047 | 0.010336665 |
| *Psmd12* | proteasome (prosome, macropain) 26S subunit, non-ATPase, 12 | 1.073144531 | 0.000101565 |
| *Nfatc2ip* | nuclear factor of activated T cells, cytoplasmic, calcineurin dependent 2 interacting protein | 1.072773437 | 0.005206323 |
| *Msn* | moesin | 1.072207031 | 0.005978289 |
| *Galt* | galactose-1-phosphate uridyl transferase | 1.070703125 | 0.034693029 |
| *Qars* | glutaminyl-tRNA synthetase | 1.065957031 | 0.004817579 |
| *Commd6* | COMM domain containing 6 | 1.065546875 | 0.048764714 |
| *Cystm1* | cysteine-rich transmembrane module containing 1 | 1.065039063 | 0.049284649 |
| *Sec16a* | SEC16 homolog A, endoplasmic reticulum export factor | 1.062304688 | 0.002233301 |
| *Idh3b* | isocitrate dehydrogenase 3 (NAD+) beta | 1.061650391 | 0.003063462 |
| *Otud7b* | OTU domain containing 7B | 1.061142578 | 0.001268566 |
| *Ssr3* | signal sequence receptor, gamma | 1.060537109 | 0.000139114 |
| *Gdap2* | ganglioside-induced differentiation-associated-protein 2 | 1.060214844 | 0.012160837 |
| *Psmb4* | proteasome (prosome, macropain) subunit, beta type 4 | 1.059707031 | 8.17539E-05 |
| *1110008F13Rik* | RIKEN cDNA 1110008F13 gene | 1.05953125 | 0.007062926 |
| *Rack1* | receptor for activated C kinase 1 | 1.057695312 | 0.005534822 |
| *Ptgr2* | prostaglandin reductase 2 | 1.057509766 | 0.000931245 |
| *Lsm7* | LSM7 homolog, U6 small nuclear RNA and mRNA degradation associated | 1.05734375 | 0.007132143 |
| *Rsbn1* | rosbin, round spermatid basic protein 1 | 1.056767578 | 0.006099964 |
| *Lsm4* | LSM4 homolog, U6 small nuclear RNA and mRNA degradation associated | 1.056582031 | 0.024410782 |
| *Galk2* | galactokinase 2 | 1.056533203 | 0.006730981 |
| *Ints4* | integrator complex subunit 4 | 1.056162109 | 0.006752265 |
| *Bckdha* | branched chain ketoacid dehydrogenase E1, alpha polypeptide | 1.055771484 | 0.026346527 |
| *Sbno1* | strawberry notch 1 | 1.055195313 | 0.010676069 |
| *Zswim8* | zinc finger SWIM-type containing 8 | 1.054472656 | 0.020351205 |
| *Tex261* | testis expressed gene 261 | 1.051894531 | 0.046862535 |
| *Selenow* | selenoprotein W | 1.048984375 | 0.010484191 |
| *Shoc2* | soc-2 (suppressor of clear) homolog (C. elegans) | 1.048759766 | 0.005385279 |
| *4932438A13Rik* | RIKEN cDNA 4932438A13 gene | 1.048720703 | 0.000473772 |
| *Dnttip2* | deoxynucleotidyltransferase, terminal, interacting protein 2 | 1.04828125 | 0.019560717 |
| *1810037I17Rik* | RIKEN cDNA 1810037I17 gene | 1.043242188 | 0.045667148 |
| *Pik3cb* | phosphatidylinositol-4,5-bisphosphate 3-kinase catalytic subunit beta | 1.040155744 | 0.023027248 |
| *Brox* | BRO1 domain and CAAX motif containing | 1.036884766 | 0.033139976 |
| *Pfdn1* | prefoldin 1 | 1.036630859 | 0.001028403 |
| *Ces2g* | carboxylesterase 2G | 1.035917969 | 0.028155712 |
| *Gnl2* | guanine nucleotide binding protein-like 2 (nucleolar) | 1.035888672 | 0.001663332 |
| *Rps19-ps4* | ribosomal protein S19, pseudogene 4 | 1.033291016 | 0.002252102 |
| *Cyc1* | cytochrome c-1 | 1.031767578 | 0.001509647 |
| *Htatip2* | HIV-1 Tat interactive protein 2 | 1.031074219 | 0.000100174 |
| *Arfgef1* | ADP-ribosylation factor guanine nucleotide-exchange factor 1(brefeldin A-inhibited) | 1.025957031 | 0.031092947 |
| *Gm5614* | ribosomal protein L36 pseudogene | 1.020976563 | 0.014810131 |
| *Slc2a1* | solute carrier family 2 (facilitated glucose transporter), member 1 | 1.01972168 | 0.007141874 |
| *Vps11* | VPS11, CORVET/HOPS core subunit | 1.018818359 | 0.019273594 |
| *LOC106740* | uncharacterized LOC106740 | 1.018662109 | 0.032647181 |
| *Pi4kb* | phosphatidylinositol 4-kinase beta | 1.017128906 | 0.004893912 |
| *Ndufaf1* | NADH dehydrogenase (ubiquinone) 1 alpha subcomplex, assembly factor 1 | 1.016396484 | 0.016172893 |
| *Nisch* | nischarin | 1.016337891 | 0.00211306 |
| *Lrp10* | low-density lipoprotein receptor-related protein 10 | 1.014492188 | 0.014134761 |
| *Pak4* | p21 protein (Cdc42/Rac)-activated kinase 4 | 1.014277344 | 0.001845859 |
| *Tmem9* | transmembrane protein 9 | 1.014189453 | 0.026584673 |
| *Farsa* | phenylalanyl-tRNA synthetase, alpha subunit | 1.01265625 | 0.035950033 |
| *Trappc1* | trafficking protein particle complex 1 | 1.007636719 | 0.037721683 |
| *Nmt1* | N-myristoyltransferase 1 | 1.004433424 | 5.65099E-05 |
| *Rab22a* | RAB22A, member RAS oncogene family | 1.004379883 | 0.00157125 |
| *Ube3a* | ubiquitin protein ligase E3A | 1.004238281 | 0.006099964 |
| *Tbccd1* | TBCC domain containing 1 | -1.002099609 | 0.027379928 |
| *Ago2* | argonaute RISC catalytic subunit 2 | -1.0021875 | 0.001201743 |
| *Gna12* | guanine nucleotide binding protein, alpha 12 | -1.002333984 | 0.002520559 |
| *Gm7676* | interferon induced transmembrane protein 1 pseudogene | -1.004462891 | 0.003820415 |
| *Flii* | flightless I actin binding protein | -1.006425781 | 0.009417604 |
| *Bola3* | bolA-like 3 (E. coli) | -1.008476563 | 0.012003299 |
| *H2afv* | H2A histone family, member V | -1.008759766 | 0.048970692 |
| *Unc5cl* | unc-5 family C-terminal like | -1.019238281 | 0.018318748 |
| *Selenbp2* | selenium binding protein 2 | -1.020224609 | 0.034827429 |
| *Bloc1s1* | biogenesis of lysosomal organelles complex-1, subunit 1 | -1.020244141 | 0.004441644 |
| *Uqcr11* | ubiquinol-cytochrome c reductase, complex III subunit XI | -1.020546875 | 0.0280505 |
| *Ube2s* | ubiquitin-conjugating enzyme E2S | -1.023554688 | 0.020152815 |
| *Gm10080* | predicted gene 10080 | -1.0253125 | 0.041826286 |
| *Akr1b3* | aldo-keto reductase family 1, member B3 (aldose reductase) | -1.028388672 | 0.012269259 |
| *Gm10785* | predicted gene 10785 | -1.03125 | 0.007009903 |
| *Nmi* | N-myc (and STAT) interactor | -1.032421875 | 0.047789001 |
| *Slc12a6* | solute carrier family 12, member 6 | -1.034003906 | 0.044113015 |
| *Gdi1* | guanosine diphosphate (GDP) dissociation inhibitor 1 | -1.034492188 | 0.004589494 |
| *Ube4a* | ubiquitination factor E4A | -1.035927734 | 0.011495494 |
| *Vat1* | vesicle amine transport 1 | -1.036420898 | 0.031907647 |
| *Lpcat3* | lysophosphatidylcholine acyltransferase 3 | -1.036943359 | 0.023258781 |
| *Pnp2* | purine-nucleoside phosphorylase 2 | -1.03765625 | 0.019868957 |
| *Fads3* | fatty acid desaturase 3 | -1.039076773 | 0.023515663 |
| *Gltp* | glycolipid transfer protein | -1.040625 | 0.033739587 |
| *Fcna* | ficolin A | -1.042392578 | 0.048870535 |
| *Ddx58* | DEAD (Asp-Glu-Ala-Asp) box polypeptide 58 | -1.043935547 | 0.004219827 |
| *Fam32a* | family with sequence similarity 32, member A | -1.047382813 | 0.009014626 |
| *Zbtb45* | zinc finger and BTB domain containing 45 | -1.052666016 | 0.025004247 |
| *Ppbp* | pro-platelet basic protein | -1.053491211 | 0.013298719 |
| *AI480526* | expressed sequence AI480526 | -1.055639648 | 0.006109126 |
| *Tnk1* | tyrosine kinase, non-receptor, 1 | -1.056582031 | 0.003296153 |
| *B2m* | beta-2 microglobulin | -1.057324219 | 0.002552463 |
| *Swt1* | SWT1 RNA endoribonuclease homolog (S. cerevisiae) | -1.057753906 | 0.019800573 |
| *D030056L22Rik* | RIKEN cDNA D030056L22 gene | -1.058862305 | 0.002155829 |
| *Ap1ar* | adaptor-related protein complex 1 associated regulatory protein | -1.060712891 | 0.012509568 |
| *Mvb12b* | multivesicular body subunit 12B | -1.062314454 | 0.008032702 |
| *Ptpn12* | protein tyrosine phosphatase, non-receptor type 12 | -1.063759766 | 0.002080031 |
| *Fam50a* | family with sequence similarity 50, member A | -1.065029297 | 0.038548336 |
| *Dennd2c* | DENN/MADD domain containing 2C | -1.066591797 | 0.016858224 |
| *Bbs7* | Bardet-Biedl syndrome 7 (human) | -1.066728516 | 0.014137985 |
| *Dnmt1* | DNA methyltransferase (cytosine-5) 1 | -1.067987119 | 0.035417463 |
| *Hace1* | HECT domain and ankyrin repeat containing, E3 ubiquitin protein ligase 1 | -1.070585937 | 0.00012328 |
| *Rcl1* | RNA terminal phosphate cyclase-like 1 | -1.072285156 | 0.002427369 |
| *Golga3* | golgi autoantigen, golgin subfamily a, 3 | -1.074238281 | 0.041589914 |
| *Hnrnpul1* | heterogeneous nuclear ribonucleoprotein U-like 1 | -1.076474609 | 9.45063E-05 |
| *Mical3* | microtubule associated monooxygenase, calponin and LIM domain containing 3 | -1.084394531 | 0.001597545 |
| *Srek1ip1* | splicing regulatory glutamine/lysine-rich protein 1interacting protein 1 | -1.087539062 | 0.016151026 |
| *Gm10244* | predicted gene 10244 | -1.087597656 | 0.00723317 |
| *Sde2* | SDE2 telomere maintenance homolog (S. pombe) | -1.087822266 | 0.023883307 |
| *Katnal1* | katanin p60 subunit A-like 1 | -1.090947266 | 0.007643404 |
| *Usp25* | ubiquitin specific peptidase 25 | -1.094316406 | 0.008361625 |
| *Timm17b* | translocase of inner mitochondrial membrane 17b | -1.094765625 | 0.034446769 |
| *Dleu2* | deleted in lymphocytic leukemia, 2 | -1.0953125 | 0.016640302 |
| *Cenpf* | centromere protein F | -1.095957031 | 0.012470217 |
| *Kcnj10* | potassium inwardly-rectifying channel, subfamily J, member 10 | -1.098256836 | 0.005054421 |
| *Ccl22* | chemokine (C-C motif) ligand 22 | -1.09878418 | 0.014268098 |
| *Limd1* | LIM domains containing 1 | -1.109697266 | 0.017655953 |
| *Ncoa7* | nuclear receptor coactivator 7 | -1.115644531 | 0.010685584 |
| *9330188P03Rik* | RIKEN cDNA 9330188P03 gene | -1.118525391 | 0.003900689 |
| *Myl12b* | myosin, light chain 12B, regulatory | -1.122197266 | 0.008170384 |
| *Nfix* | nuclear factor I/X | -1.125332031 | 0.001166454 |
| *Rfx2* | regulatory factor X, 2 (influences HLA class II expression) | -1.128574219 | 0.031336283 |
| *Zfp526* | zinc finger protein 526 | -1.128691406 | 0.043234488 |
| *Pop5* | processing of precursor 5, ribonuclease P/MRP family (S. cerevisiae) | -1.129746094 | 0.000251147 |
| *Clgn* | calmegin | -1.132348633 | 0.010047526 |
| *Fcer1g* | Fc receptor, IgE, high affinity I, gamma polypeptide | -1.134511719 | 0.017104332 |
| *Klhl12* | kelch-like 12 | -1.137802734 | 0.005615415 |
| *Psmb9* | proteasome (prosome, macropain) subunit, beta type 9 (large multifunctional peptidase 2) | -1.14706543 | 0.00328876 |
| *9130221H12Rik* | RIKEN cDNA 9130221H12 gene | -1.147929688 | 0.007129004 |
| *Sri* | sorcin | -1.150058594 | 0.002054241 |
| *Ccdc167* | coiled-coil domain containing 167 | -1.153183594 | 0.023600672 |
| *Inpp5d* | inositol polyphosphate-5-phosphatase D | -1.154941406 | 0.009874783 |
| *Vamp5* | vesicle-associated membrane protein 5 | -1.156757813 | 0.000551222 |
| *Plpp1* | phospholipid phosphatase 1 | -1.158251953 | 0.006615022 |
| *Lst1* | leukocyte specific transcript 1 | -1.158544922 | 0.011358359 |
| *Mcee* | methylmalonyl CoA epimerase | -1.163579102 | 0.036477297 |
| *Syt14* | synaptotagmin XIV | -1.165078125 | 0.040301545 |
| *LOC102634065* | protein transport protein Sec61 subunit gamma pseudogene | -1.165888672 | 0.046145843 |
| *Arl2bp* | ADP-ribosylation factor-like 2 binding protein | -1.170693359 | 0.009586815 |
| *Plbd1* | phospholipase B domain containing 1 | -1.171943359 | 0.039676502 |
| *Mpeg1* | macrophage expressed gene 1 | -1.173017578 | 0.004174819 |
| *Gch1* | GTP cyclohydrolase 1 | -1.17484375 | 3.7066E-05 |
| *Cfp* | complement factor properdin | -1.174887695 | 0.00867754 |
| *Trappc2l* | trafficking protein particle complex 2-like | -1.180908203 | 0.049284649 |
| *Pacs2* | phosphofurin acidic cluster sorting protein 2 | -1.183193359 | 0.013951278 |
| *Cyth4* | cytohesin 4 | -1.190141602 | 0.037705213 |
| *Tusc1* | tumor suppressor candidate 1 | -1.192631742 | 0.000572288 |
| *Fam213b* | family with sequence similarity 213, member B | -1.193046875 | 0.006479969 |
| *Hbb-bh1* | hemoglobin Z, beta-like embryonic chain | -1.20355957 | 0.003225742 |
| *Ethe1* | ethylmalonic encephalopathy 1 | -1.204189453 | 0.006038981 |
| *Irak1* | interleukin-1 receptor-associated kinase 1 | -1.208203125 | 0.00057362 |
| *Naaladl1* | N-acetylated alpha-linked acidic dipeptidase-like 1 | -1.209575195 | 0.002537405 |
| *Parvb* | parvin, beta | -1.21515625 | 0.005296507 |
| *Cib3* | calcium and integrin binding family member 3 | -1.22015625 | 0.008368233 |
| *2700097O09Rik* | RIKEN cDNA 2700097O09 gene | -1.223276367 | 0.004709416 |
| *Ammecr1* | Alport syndrome, mental retardation, midface hypoplasia and elliptocytosis chromosomal region gene 1 | -1.224179688 | 0.033722223 |
| *Gipc2* | GIPC PDZ domain containing family, member 2 | -1.230527344 | 0.005889819 |
| *Hcls1* | hematopoietic cell specific Lyn substrate 1 | -1.238408203 | 0.019612637 |
| *Anxa5* | annexin A5 | -1.238535156 | 0.007207985 |
| *Sirpa* | signal-regulatory protein alpha | -1.243085938 | 0.002569473 |
| *Golga7* | golgi autoantigen, golgin subfamily a, 7 | -1.24453125 | 0.016965098 |
| *Gm38448* | predicted gene, 38448 | -1.245810547 | 0.023021438 |
| *2010109A12Rik* | RIKEN cDNA 2010109A12 gene | -1.248662109 | 0.017537638 |
| *Oip5* | Opa interacting protein 5 | -1.249375 | 0.003494425 |
| *Chfr* | checkpoint with forkhead and ring finger domains | -1.250498047 | 0.005743516 |
| *Tbc1d10b* | TBC1 domain family, member 10b | -1.253759766 | 0.009100684 |
| *Cpeb3* | cytoplasmic polyadenylation element binding protein 3 | -1.260546875 | 9.1097E-05 |
| *Ogdh* | oxoglutarate (alpha-ketoglutarate) dehydrogenase (lipoamide) | -1.261894471 | 0.000512272 |
| *Grcc10* | gene rich cluster, C10 gene | -1.27125 | 0.000248825 |
| *Ugdh* | UDP-glucose dehydrogenase | -1.278085938 | 0.041151279 |
| *Vapa* | vesicle-associated membrane protein, associated protein A | -1.278525391 | 0.002246948 |
| *Slc38a9* | solute carrier family 38, member 9 | -1.281318359 | 0.000228912 |
| *Sec61g* | SEC61, gamma subunit | -1.281601563 | 0.008082323 |
| *Tifa* | TRAF-interacting protein with forkhead-associated domain | -1.282490234 | 0.015725267 |
| *Ell2* | elongation factor RNA polymerase II 2 | -1.282734375 | 0.014256681 |
| *Extl3* | exostoses (multiple)-like 3 | -1.282919922 | 0.02905 |
| *Anapc13* | anaphase promoting complex subunit 13 | -1.283613281 | 0.021064613 |
| *Ap2a1* | adaptor-related protein complex 2, alpha 1 subunit | -1.287822266 | 0.000824195 |
| *Unc93b1* | unc-93 homolog B1 (C. elegans) | -1.288564453 | 0.000905663 |
| *Lrpprc* | leucine-rich PPR-motif containing | -1.2909375 | 0.003646514 |
| *2310010J17Rik* | RIKEN cDNA 2310010J17 gene | -1.292260742 | 7.36647E-05 |
| *6030458C11Rik* | RIKEN cDNA 6030458C11 gene | -1.292900391 | 0.021938433 |
| *Nup133* | nucleoporin 133 | -1.294580078 | 0.035021059 |
| *Ifih1* | interferon induced with helicase C domain 1 | -1.2946875 | 0.033720104 |
| *Ccdc18* | coiled-coil domain containing 18 | -1.298125 | 0.00132514 |
| *Ncf4* | neutrophil cytosolic factor 4 | -1.29888194 | 0.01746759 |
| *Hist1h2bp* | histone cluster 1, H2bp | -1.307470703 | 0.033408293 |
| *Myh10* | myosin, heavy polypeptide 10, non-muscle | -1.312353516 | 0.003605199 |
| *Btrc* | beta-transducin repeat containing protein | -1.313857422 | 0.000383993 |
| *Dpf3* | D4, zinc and double PHD fingers, family 3 | -1.313974609 | 4.54942E-05 |
| *Dcun1d1* | DCN1, defective in cullin neddylation 1, domain containing 1 (S. cerevisiae) | -1.317734375 | 0.03128724 |
| *Txn2* | thioredoxin 2 | -1.322851563 | 0.001531007 |
| *Dtd2* | D-tyrosyl-tRNA deacylase 2 | -1.323134766 | 0.016906214 |
| *Cd68* | CD68 antigen | -1.323364258 | 0.013653578 |
| *Lgals1* | lectin, galactose binding, soluble 1 | -1.333056641 | 0.020508315 |
| *Sdc3* | syndecan 3 | -1.33375 | 0.005020032 |
| *Optn* | optineurin | -1.334101563 | 0.0008427 |
| *Sertad3* | SERTA domain containing 3 | -1.336308593 | 0.01377566 |
| *Tmco6* | transmembrane and coiled-coil domains 6 | -1.350292969 | 0.00281652 |
| *Vmn1r9* | vomeronasal 1 receptor 9 | -1.350527344 | 0.032964283 |
| *Med21* | mediator complex subunit 21 | -1.362363281 | 0.032639331 |
| *1700020I14Rik* | RIKEN cDNA 1700020I14 gene | -1.363085938 | 0.00500429 |
| *Trim26* | tripartite motif-containing 26 | -1.368808594 | 0.009632761 |
| *Gm13822* | predicted gene 13822 | -1.369682617 | 0.03257347 |
| *Ap3m1* | adaptor-related protein complex 3, mu 1 subunit | -1.383515625 | 0.004350929 |
| *Myl6* | myosin, light polypeptide 6, alkali, smooth muscle and non-muscle | -1.383652344 | 0.045651412 |
| *Pfas* | phosphoribosylformylglycinamidine synthase (FGAR amidotransferase) | -1.384169922 | 0.008025107 |
| *Axl* | AXL receptor tyrosine kinase | -1.398515625 | 0.007362653 |
| *Pea15a* | phosphoprotein enriched in astrocytes 15A | -1.40293457 | 0.000349065 |
| *Gm9949* | predicted gene 9949 | -1.408466797 | 0.019868336 |
| *Foxp1* | forkhead box P1 | -1.408662109 | 0.000248825 |
| *9530026F06Rik* | RIKEN cDNA 9530026F06 gene | -1.410874023 | 0.003651966 |
| *Zfp808* | zinc finger protein 80 | -1.4128125 | 0.0348608 |
| *Lgals3* | lectin, galactose binding, soluble 3 | -1.413642578 | 0.007451985 |
| *Hspbap1* | Hspb associated protein 1 | -1.414990234 | 0.008302899 |
| *Laptm5* | lysosomal-associated protein transmembrane 5 | -1.418134766 | 0.034629479 |
| *Rnf14* | ring finger protein 14 | -1.422841797 | 0.009328184 |
| *Selplg* | selectin, platelet (p-selectin) ligand | -1.425830078 | 0.004019497 |
| *Foxn3* | forkhead box N3 | -1.4271875 | 0.004293654 |
| *Gm39590* | predicted gene, 39590 | -1.440756836 | 0.000148035 |
| *Aldh1a1* | aldehyde dehydrogenase family 1, subfamily A1 | -1.444570313 | 0.007683349 |
| *Rab18* | RAB18, member RAS oncogene family | -1.444716797 | 0.046796287 |
| *Tm7sf3* | transmembrane 7 superfamily member 3 | -1.446533203 | 0.002050447 |
| *Slc9a8* | solute carrier family 9 (sodium/hydrogen exchanger), member 8 | -1.448564453 | 0.000852492 |
| *Nap1l4* | nucleosome assembly protein 1-like 4 | -1.451726752 | 0.000144891 |
| *Gm15787* | predicted gene 15787 | -1.452890625 | 0.008730729 |
| *Hbb-y* | hemoglobin Y, beta-like embryonic chain | -1.455976563 | 0.002180912 |
| *Gyg* | glycogenin | -1.459291992 | 0.003614676 |
| *Man1a* | mannosidase 1, alpha | -1.466435547 | 0.001073282 |
| *Asb3* | ankyrin repeat and SOCS box-containing 3 | -1.475449219 | 0.006414615 |
| *Stx7* | syntaxin 7 | -1.475986328 | 0.000521514 |
| *Gm20337* | predicted gene, 20337 | -1.48503418 | 0.000575451 |
| *Ldlrad4* | low density lipoprotein receptor class A domain containing 4 | -1.486694336 | 0.003044849 |
| *Neurl3* | neuralized E3 ubiquitin protein ligase 3 | -1.504746094 | 6.16865E-05 |
| *Pttg1ip* | pituitary tumor-transforming 1 interacting protein | -1.504882813 | 0.008682533 |
| *G3bp2* | GTPase activating protein (SH3 domain) binding protein 2 | -1.518398438 | 0.02484916 |
| *Stat2* | signal transducer and activator of transcription 2 | -1.540371094 | 0.001437329 |
| *Rpph1* | ribonuclease P RNA component H1 | -1.541191406 | 0.014809548 |
| *Rbbp4* | retinoblastoma binding protein 4, chromatin remodeling factor | -1.559277344 | 0.001117545 |
| *Cul4a* | cullin 4A | -1.589179688 | 0.002762912 |
| *Irf2* | interferon regulatory factor 2 | -1.593007813 | 0.002047056 |
| *Asap2* | ArfGAP with SH3 domain, ankyrin repeat and PH domain 2 | -1.595375977 | 0.001080297 |
| *Hebp1* | heme binding protein 1 | -1.597568359 | 0.000163595 |
| *Tyrobp* | TYRO protein tyrosine kinase binding protein | -1.602695313 | 0.025368563 |
| *Ank* | progressive ankylosis | -1.608769531 | 0.00045309 |
| *Fads1* | fatty acid desaturase 1 | -1.612246094 | 0.007505423 |
| *Cmpk2* | cytidine monophosphate (UMP-CMP) kinase 2, mitochondrial | -1.61265625 | 0.02604254 |
| *Grap2* | GRB2-related adaptor protein 2 | -1.615136719 | 0.000357745 |
| *Adck5* | aarF domain containing kinase 5 | -1.616611328 | 0.002891093 |
| *Pts* | 6-pyruvoyl-tetrahydropterin synthase | -1.616640625 | 0.000208183 |
| *Rfxank* | regulatory factor X-associated ankyrin-containing protein | -1.619130859 | 0.016877663 |
| *Ifit1bl1* | interferon induced protein with tetratricpeptide repeats 1B like 1 | -1.620698242 | 0.029725287 |
| *Lrrc39* | leucine rich repeat containing 39 | -1.623520508 | 0.000292577 |
| *Selenop* | selenoprotein P | -1.629277344 | 0.049878029 |
| *1700001K23Rik* | RIKEN cDNA 1700001K23 gene | -1.634970703 | 0.002653957 |
| *Gm16793* | predicted gene, 16793 | -1.643105469 | 0.031721642 |
| *H2-Aa* | histocompatibility 2, class II antigen A, alpha | -1.645771484 | 0.041164976 |
| *Brk1* | BRICK1, SCAR/WAVE actin-nucleating complex subunit | -1.652724609 | 0.001678999 |
| *4930430F08Rik* | RIKEN cDNA 4930430F08 gene | -1.666938477 | 0.006820148 |
| *Mylip* | myosin regulatory light chain interacting protein | -1.668886719 | 0.002828682 |
| *Polr3b* | polymerase (RNA) III (DNA directed) polypeptide B | -1.678730469 | 0.000487469 |
| *Cit* | citron | -1.681240234 | 0.007836843 |
| *Sgk3* | serum/glucocorticoid regulated kinase 3 | -1.692822266 | 0.000197505 |
| *Ankrd28* | ankyrin repeat domain 28 | -1.693300781 | 0.002582017 |
| *Hbq1a* | hemoglobin, theta 1A | -1.695644531 | 0.036943629 |
| *Gm15915* | predicted gene 15915 | -1.699667969 | 0.001565588 |
| *Cd74* | CD74 antigen (invariant polypeptide of major histocompatibility complex, class II antigen-associated) | -1.704160156 | 0.00630176 |
| *Mapre2* | microtubule-associated protein, RP/EB family, member 2 | -1.707119141 | 0.011179957 |
| *Ndrg2* | N-myc downstream regulated gene 2 | -1.713505859 | 0.046682368 |
| *Irf7* | interferon regulatory factor 7 | -1.730722656 | 0.012574906 |
| *Cacna1g* | calcium channel, voltage-dependent, T type, alpha 1G subunit | -1.731152344 | 0.007869105 |
| *Clec7a* | C-type lectin domain family 7, member a | -1.745170898 | 0.011248661 |
| *Ogt* | O-linked N-acetylglucosamine (GlcNAc) transferase (UDP-N-acetylglucosamine:polypeptide-N-acetylglucosaminyl transferase) | -1.757011719 | 0.000799213 |
| *Tmem65* | transmembrane protein 65 | -1.764550781 | 0.000356384 |
| *Slc44a1* | solute carrier family 44, member 1 | -1.769082031 | 5.65326E-05 |
| *Scarna13* | small Cajal body-specific RNA 1 | -1.796728516 | 0.003098968 |
| *Lgals3bp* | lectin, galactoside-binding, soluble, 3 binding protein | -1.798188477 | 0.000886043 |
| *Rnf216* | ring finger protein 216 | -1.798701172 | 1.00111E-05 |
| *St5* | suppression of tumorigenicity 5 | -1.801191406 | 0.00960889 |
| *Gm15518* | predicted gene 15518 | -1.812861328 | 0.004988713 |
| *Igsf6* | immunoglobulin superfamily, member 6 | -1.81440918 | 0.040569223 |
| *1110034G24Rik* | RIKEN cDNA 1110034G24 gene | -1.819306641 | 0.000109043 |
| *Aif1* | allograft inflammatory factor 1 | -1.823037109 | 0.002074911 |
| *Def8* | differentially expressed in FDCP 8 | -1.849306641 | 0.01649739 |
| *Slc25a44* | solute carrier family 25, member 44 | -1.852949219 | 0.000724657 |
| *Trim34a* | tripartite motif-containing 34A | -1.871914063 | 0.003443038 |
| *Ap5s1* | adaptor-related protein 5 complex, sigma 1 subunit | -1.89515625 | 0.000368658 |
| *Agtr1a* | angiotensin II receptor, type 1a | -1.917353516 | 0.013595489 |
| *Fancd2* | Fanconi anemia, complementation group D2 | -1.946044922 | 0.004486971 |
| *Cbr1* | carbonyl reductase 1 | -1.94875 | 0.001837116 |
| *Ccdc6* | coiled-coil domain containing 6 | -2.008378906 | 7.81191E-06 |
| *Samd9l* | sterile alpha motif domain containing 9-like | -2.01546875 | 0.025240667 |
| *Ppdpf* | pancreatic progenitor cell differentiation and proliferation factor | -2.046494141 | 0.006685013 |
| *Ogfrl1* | opioid growth factor receptor-like 1 | -2.056611328 | 0.001328967 |
| *AA388235* | expressed sequence AA388235 | -2.060732422 | 4.26296E-05 |
| *Pkd1l1* | polycystic kidney disease 1 like 1 | -2.086992188 | 0.004734117 |
| *Parp12* | poly (ADP-ribose) polymerase family, member 12 | -2.090234375 | 0.016379059 |
| *Aldh3b2* | aldehyde dehydrogenase 3 family, member B2 | -2.102158203 | 0.000551696 |
| *Tet3* | tet methylcytosine dioxygenase 3 | -2.115488281 | 0.001274152 |
| *Anp32e* | acidic (leucine-rich) nuclear phosphoprotein 32 family, member E | -2.131279297 | 4.37799E-05 |
| *Gm10371* | predicted gene 10371 | -2.188710938 | 0.013075214 |
| *Cetn2* | centrin 2 | -2.200800781 | 0.004910191 |
| *Fbxw2* | F-box and WD-40 domain protein 2 | -2.295214844 | 0.001271865 |
| *Runx1t1* | runt-related transcription factor 1; translocated to, 1 (cyclin D-related) | -2.378544922 | 0.000315164 |
| *H3f3a* | H3 histone, family 3A | -2.415400391 | 0.012590788 |
| *Ppp1r13b* | protein phosphatase 1, regulatory (inhibitor) subunit 13B | -2.421787109 | 0.00110231 |
| *Grk5* | G protein-coupled receptor kinase 5 | -2.471289063 | 0.002575233 |
| *Scd1* | stearoyl-Coenzyme A desaturase 1 | -2.535488281 | 5.2084E-05 |
| *Selenbp1* | selenium binding protein 1 | -2.577529297 | 0.000273774 |
| *Fam107b* | family with sequence similarity 107, member B | -2.628916016 | 0.000101565 |
| *Cnnm2* | cyclin M2 | -2.693837891 | 5.34176E-05 |
| *Ssh1* | slingshot protein phosphatase 1 | -2.801083984 | 0.00029169 |
| *Pepd* | peptidase D | -2.907094727 | 9.65758E-05 |
| *Xaf1* | XIAP associated factor 1 | -2.969169922 | 0.00304442 |
| *Chst10* | carbohydrate sulfotransferase 10 | -3.0425 | 0.002543811 |
| *Sqor* | sulfide quinone oxidoreductase | -3.458740234 | 4.62829E-05 |
| *Gm14719* | predicted gene 14719 | -3.962529297 | 0.000492575 |
