# Supplementary material for: Bone marrow sinusoidal endothelium controls terminal erythroid differentiation and reticulocyte maturation
Source: Nat Commun. 2021 Nov 29;12:6963. doi: 10.1038/s41467-021-27161-3 (PMC8630019; doi:10.1038/s41467-021-27161-3)
Supplement: Supplementary file 5 — Supplementary Data 2 [file 41467_2021_27161_MOESM5_ESM.docx]

**Supplementary Data file 2. Significantly differentially expressed genes (DEGs) in** **PIV of *Ctnnb1^OE-SEC^* mice compared to *Ctnnb1^WT^* controls.**

Genes displayed are those significantly up or down regulated in PIV of *Ctnnb1^OE-SEC^* compared to *Ctnnb1^WT^* controls (FC > |1|). Adjusted p-values were calculated for the differences of means of log2 of expressions values between PIV of *Ctnnb1^OE-SEC^* mice and *Ctnnb1^WT^*. OneWay-ANOVA was performed to identify DEGs.

| ***Gene Symbol*** | **Gene Title** | **Fold change *Ctnnb1^OE-SEC^* > *Ctnnb1^WT^*** | **Adjusted p-Value for Diff of experiment = *Ctnnb1^OE-SEC^* - *Ctnnb1^WT^*** |
| --- | --- | --- | --- |
| *Mt2* | metallothionein 2 | 2.978710938 | 0.000241142 |
| *4930594O21Rik* | RIKEN cDNA 4930594O21 gene | 2.9671875 | 0.026087269 |
| *Klhl21* | kelch-like 21 | 2.966015625 | 1.9041E-05 |
| *Tsc22d2* | TSC22 domain family, member 2 | 2.946875 | 1.00111E-05 |
| *Ankrd36* | ankyrin repeat domain 36 | 2.874023438 | 6.39191E-05 |
| *Sesn2* | sestrin 2 | 2.845507813 | 8.21406E-05 |
| *Cyb5r1* | cytochrome b5 reductase 1 | 2.836132813 | 4.67103E-05 |
| *Reep6* | receptor accessory protein 6 | 2.819335938 | 1.62151E-05 |
| *2610318N02Rik* | RIKEN cDNA 2610318N02 gene | 2.759472656 | 2.5434E-05 |
| *Gmpr* | guanosine monophosphate reductase | 2.714257813 | 0.000909706 |
| *Aaed1* | AhpC/TSA antioxidant enzyme domain containing 1 | 2.696679688 | 0.000428984 |
| *Calcrl* | calcitonin receptor-like | 2.68984375 | 0.001183116 |
| *Hspa9* | heat shock protein 9 | 2.657617188 | 1.27754E-05 |
| *Lgals8* | lectin, galactose binding, soluble 8 | 2.650585938 | 0.000189846 |
| *Srp72* | signal recognition particle 72 | 2.643945313 | 3.10244E-07 |
| *Tubb3* | tubulin, beta 3 class III | 2.570605469 | 0.000898311 |
| *Dennd5a* | DENN/MADD domain containing 5A | 2.566992188 | 0.002771512 |
| *Plxnc1* | plexin C1 | 2.547070313 | 0.001271865 |
| *Trim15* | tripartite motif-containing 15 | 2.466015625 | 0.000431523 |
| *Agfg1* | ArfGAP with FG repeats 1 | 2.390234375 | 4.23734E-05 |
| *4930523C07Rik* | RIKEN cDNA 4930523C07 gene | 2.38515625 | 0.001568522 |
| *Got1* | glutamic-oxaloacetic transaminase 1, soluble | 2.377636719 | 0.000230994 |
| *Pnpla2* | patatin-like phospholipase domain containing 2 | 2.377148437 | 0.001272163 |
| *Il4ra* | interleukin 4 receptor, alpha | 2.364160156 | 0.010091207 |
| *Mob3a* | MOB kinase activator 3A | 2.359570313 | 0.005092711 |
| *Ndrg3* | N-myc downstream regulated gene 3 | 2.339648438 | 5.53053E-06 |
| *Arl6ip5* | ADP-ribosylation factor-like 6 interacting protein 5 | 2.330664062 | 1.75312E-05 |
| *Braf* | Braf transforming gene | 2.327929688 | 0.000393362 |
| *Eif4ebp1* | eukaryotic translation initiation factor 4E binding protein 1 | 2.29921875 | 0.007672235 |
| *Rph3al* | rabphilin 3A-like (without C2 domains) | 2.287304688 | 0.002043283 |
| *Eif3c* | eukaryotic translation initiation factor 3, subunit C | 2.280078125 | 0.000265996 |
| *Gss* | glutathione synthetase | 2.271679688 | 0.015971771 |
| *Selenow* | selenoprotein W | 2.260742188 | 4.00184E-05 |
| *Erfe* | erythroferrone | 2.252636719 | 0.000141895 |
| *Spata1* | spermatogenesis associated 1 | 2.243554688 | 0.004514441 |
| *Chac1* | ChaC, cation transport regulator 1 | 2.243261719 | 0.024632782 |
| *Ddit3* | DNA-damage inducible transcript 3 | 2.225 | 0.001712156 |
| *Prpf6* | pre-mRNA splicing factor 6 | 2.206054687 | 3.10608E-06 |
| *Gm3150* | guanine nucleotide binding protein (G protein), gamma 5 pseudogene | 2.2 | 0.004829658 |
| *Serp1* | stress-associated endoplasmic reticulum protein 1 | 2.178125004 | 4.37799E-05 |
| *Spata13* | spermatogenesis associated 13 | 2.166796875 | 3.35226E-05 |
| *1700097N02Rik* | RIKEN cDNA 1700097N02 gene | 2.159765625 | 0.006096878 |
| *Ssbp2* | single-stranded DNA binding protein 2 | 2.15703125 | 0.000361715 |
| *Slc7a11* | solute carrier family 7 (cationic amino acid transporter, y+ system), member 11 | 2.154980469 | 0.00139501 |
| *Dhrs3* | dehydrogenase/reductase (SDR family) member 3 | 2.124414063 | 0.01227928 |
| *Mmel1* | membrane metallo-endopeptidase-like 1 | 2.121875 | 5.60474E-05 |
| *Mt1* | metallothionein 1 | 2.103222656 | 2.09265E-05 |
| *Aoc3* | amine oxidase, copper containing 3 | 2.0984375 | 0.011977797 |
| *Trafd1* | TRAF type zinc finger domain containing 1 | 2.0890625 | 0.000891894 |
| *Uhrf1bp1l* | UHRF1 (ICBP90) binding protein 1-like | 2.077539063 | 0.001780753 |
| *Klf6* | Kruppel-like factor 6 | 2.06796875 | 0.006212373 |
| *Lrp6* | low density lipoprotein receptor-related protein 6 | 2.061425781 | 0.001334554 |
| *Atf6* | activating transcription factor 6 | 2.048828125 | 4.37799E-05 |
| *Tmod3* | tropomodulin 3 | 2.03984375 | 0.01101964 |
| *Mocs2* | molybdenum cofactor synthesis 2 | 2.015625 | 7.62274E-05 |
| *Slc31a1* | solute carrier family 31, member 1 | 2.014453125 | 6.39191E-05 |
| *Mtf1* | metal response element binding transcription factor 1 | 2.009765625 | 0.000810437 |
| *Mthfr* | 5,10-methylenetetrahydrofolate reductase | 2.00078125 | 0.000142584 |
| *Iah1* | isoamyl acetate-hydrolyzing esterase 1 homolog | 1.99375 | 0.000134972 |
| *Ascc2* | activating signal cointegrator 1 complex subunit 2 | 1.986132813 | 2.66957E-05 |
| *Elp5* | elongator acetyltransferase complex subunit 5 | 1.979492188 | 8.26283E-05 |
| *Gm7334* | B-cell translocation gene 3 pseudogene | 1.97890625 | 0.00303174 |
| *Snord13* | small nucleolar RNA, C/D box 13 | 1.964160156 | 0.027894484 |
| *Ubxn4* | UBX domain protein 4 | 1.928515625 | 0.00013374 |
| *Clic1* | chloride intracellular channel 1 | 1.922070313 | 0.000866235 |
| *Gramd4* | GRAM domain containing 4 | 1.915234375 | 0.000311886 |
| *Ndrg1* | N-myc downstream regulated gene 1 | 1.915234375 | 0.032867072 |
| *Mob1b* | MOB kinase activator 1B | 1.91484375 | 7.81191E-06 |
| *Atp1b2* | ATPase, Na+/K+ transporting, beta 2 polypeptide | 1.909570313 | 0.009111127 |
| *Rexo2* | RNA exonuclease 2 | 1.907421875 | 6.01842E-06 |
| *Smim3* | small integral membrane protein 3 | 1.893164063 | 0.000895408 |
| *Raly* | hnRNP-associated with lethal yellow | 1.887109375 | 0.000110978 |
| *Susd2* | sushi domain containing 2 | 1.875390625 | 0.000268601 |
| *Sec23a* | SEC23 homolog A, COPII coat complex component | 1.874102808 | 0.000451229 |
| *Plaa* | phospholipase A2, activating protein | 1.862988281 | 0.001132067 |
| *Rundc3a* | RUN domain containing 3A | 1.859570313 | 0.007234725 |
| *Cacna1c* | calcium channel, voltage-dependent, L type, alpha 1C subunit | 1.858496094 | 0.000634074 |
| *Nsfl1c* | NSFL1 (p97) cofactor (p47) | 1.855078125 | 0.000261212 |
| *Zswim4* | zinc finger SWIM-type containing 4 | 1.845898438 | 0.000368769 |
| *Zcchc11* | zinc finger, CCHC domain containing 11 | 1.833007812 | 0.003965012 |
| *Adamtsl5* | ADAMTS-like 5 | 1.831054849 | 0.000668347 |
| *Mbnl2* | muscleblind like splicing factor 2 | 1.818164063 | 0.000800121 |
| *Uba1* | ubiquitin-like modifier activating enzyme 1 | 1.814453125 | 9.01399E-08 |
| *Sfxn3* | sideroflexin 3 | 1.80859375 | 0.006949988 |
| *Atp6v1b2* | ATPase, H+ transporting, lysosomal V1 subunit B2 | 1.806250004 | 7.46954E-05 |
| *Ehd1* | EH-domain containing 1 | 1.804882813 | 0.00063132 |
| *Cct3* | chaperonin containing Tcp1, subunit 3 (gamma) | 1.798632813 | 0.00055742 |
| *Uso1* | USO1 vesicle docking factor | 1.797460938 | 0.000163687 |
| *Tcf25* | transcription factor 25 (basic helix-loop-helix) | 1.795507813 | 0.001035938 |
| *Trappc3* | trafficking protein particle complex 3 | 1.79375 | 0.002741512 |
| *2410006H16Rik* | RIKEN cDNA 2410006H16 gene | 1.789860094 | 0.003957994 |
| *Ndfip1* | Nedd4 family interacting protein 1 | 1.778710938 | 0.008420059 |
| *Otub1* | OTU domain, ubiquitin aldehyde binding 1 | 1.777734375 | 0.000208183 |
| *Prokr1* | prokineticin receptor 1 | 1.766796875 | 0.000103465 |
| *Gm4285* | predicted gene 4285 | 1.765625 | 0.000995073 |
| *Zfp106* | zinc finger protein 106 | 1.764062499 | 0.000212224 |
| *Cox14* | cytochrome c oxidase assembly protein 14 | 1.758789063 | 0.000283052 |
| *Tbpl1* | TATA box binding protein-like 1 | 1.752734375 | 0.003164906 |
| *Msn* | moesin | 1.749609375 | 0.000145569 |
| *Selenoi* | selenoprotein I | 1.746679688 | 0.002913014 |
| *Shmt2* | serine hydroxymethyltransferase 2 (mitochondrial) | 1.73828125 | 0.020085991 |
| *Dynlt3* | dynein light chain Tctex-type 3 | 1.735546875 | 0.001248121 |
| *Junb* | jun B proto-oncogene | 1.73359375 | 3.52518E-05 |
| *Fam118b* | family with sequence similarity 118, member B | 1.729882813 | 0.00012736 |
| *Ppa1* | pyrophosphatase (inorganic) 1 | 1.714648437 | 0.013911505 |
| *1110012L19Rik* | RIKEN cDNA 1110012L19 gene | 1.711523438 | 0.039223173 |
| *Rnf114* | ring finger protein 114 | 1.705859375 | 0.003335179 |
| *Fbxo38* | F-box protein 38 | 1.701367188 | 0.000623625 |
| *Zfp369* | zinc finger protein 369 | 1.6890625 | 0.001906063 |
| *Eif5b* | eukaryotic translation initiation factor 5B | 1.663476563 | 0.00039972 |
| *Dohh* | deoxyhypusine hydroxylase/monooxygenase | 1.654492187 | 0.001731782 |
| *Atf5* | activating transcription factor 5 | 1.646875 | 0.001892899 |
| *Atg2a* | autophagy related 2A | 1.638867187 | 8.21406E-05 |
| *Copb1* | coatomer protein complex, subunit beta 1 | 1.629101563 | 0.003312924 |
| *Mtmr12* | myotubularin related protein 12 | 1.628320313 | 0.00281652 |
| *Crebrf* | CREB3 regulatory factor | 1.626953125 | 0.007314143 |
| *Gm4013* | predicted gene 4013 | 1.616992188 | 0.018095236 |
| *Pak4* | p21 protein (Cdc42/Rac)-activated kinase 4 | 1.615625 | 5.34176E-05 |
| *Pycr1* | pyrroline-5-carboxylate reductase 1 | 1.611621094 | 0.028556492 |
| *Tnfrsf13c* | tumor necrosis factor receptor superfamily, member 13c | 1.6046875 | 2.76051E-05 |
| *Pi4k2a* | phosphatidylinositol 4-kinase type 2 alpha | 1.59453125 | 0.029502708 |
| *Acadvl* | acyl-Coenzyme A dehydrogenase, very long chain | 1.590039063 | 0.000786051 |
| *Snap23* | synaptosomal-associated protein 23 | 1.587890625 | 0.000107983 |
| *Lrrc28* | leucine rich repeat containing 28 | 1.575585937 | 0.005820909 |
| *Tox4* | TOX high mobility group box family member 4 | 1.57467374 | 2.69033E-05 |
| *Cox6b2* | cytochrome c oxidase subunit VIb polypeptide 2 | 1.573242187 | 0.005263328 |
| *Xpr1* | xenotropic and polytropic retrovirus receptor 1 | 1.571679688 | 0.00132514 |
| *Smim4* | small integral membrane protein 4 | 1.571289062 | 0.018918083 |
| *Rwdd4a* | RWD domain containing 4A | 1.570507813 | 0.003143387 |
| *Ctsf* | cathepsin F | 1.568554688 | 0.000886043 |
| *Crlf3* | cytokine receptor-like factor 3 | 1.55546875 | 7.81191E-06 |
| *Snw1* | SNW domain containing 1 | 1.550195313 | 0.00188805 |
| *Chmp4b* | charged multivesicular body protein 4B | 1.549023438 | 0.005452637 |
| *Creb1* | cAMP responsive element binding protein 1 | 1.548046875 | 7.7709E-05 |
| *Adk* | adenosine kinase | 1.54453125 | 0.001924663 |
| *Ints13* | integrator complex subunit 13 | 1.54140625 | 0.000512457 |
| *Ppp2r5b* | protein phosphatase 2, regulatory subunit B_, beta | 1.539453125 | 5.46369E-05 |
| *Nfe2l1* | nuclear factor, erythroid derived 2,-like 1 | 1.531054688 | 2.53596E-05 |
| *Mau2* | MAU2 sister chromatid cohesion factor | 1.5265625 | 0.000427206 |
| *Vps29* | VPS29 retromer complex component | 1.525390625 | 0.00051093 |
| *Fez2* | fasciculation and elongation protein zeta 2 (zygin II) | 1.523633097 | 0.00674816 |
| *F2r* | coagulation factor II (thrombin) receptor | 1.522265625 | 0.001793169 |
| *Zmym2* | zinc finger, MYM-type 2 | 1.513476563 | 0.00951382 |
| *Ubl7* | ubiquitin-like 7 (bone marrow stromal cell-derived) | 1.5125 | 0.004410751 |
| *B4galt1* | UDP-Gal:betaGlcNAc beta 1,4- galactosyltransferase, polypeptide 1 | 1.511328125 | 0.001066751 |
| *Pgd* | phosphogluconate dehydrogenase | 1.509375 | 0.005408311 |
| *Fah* | fumarylacetoacetate hydrolase | 1.508203125 | 0.01393418 |
| *Gpd1* | glycerol-3-phosphate dehydrogenase 1 (soluble) | 1.507226563 | 0.018224155 |
| *Med16* | mediator complex subunit 16 | 1.505664062 | 0.000534977 |
| *Akap9* | A kinase (PRKA) anchor protein (yotiao) 9 | 1.504589844 | 0.006408242 |
| *Htatip2* | HIV-1 Tat interactive protein 2 | 1.50078125 | 1.00111E-05 |
| *Sars* | seryl-aminoacyl-tRNA synthetase | 1.496484375 | 0.004232323 |
| *Stk3* | serine/threonine kinase 3 | 1.48984375 | 0.006390438 |
| *Crbn* | cereblon | 1.489257813 | 0.004450406 |
| *Arhgap25* | Rho GTPase activating protein 25 | 1.488476563 | 0.002191666 |
| *Tmem123* | transmembrane protein 123 | 1.4859375 | 0.000875606 |
| *Stat3* | signal transducer and activator of transcription 3 | 1.485351563 | 0.000666169 |
| *Kdm5b* | lysine (K)-specific demethylase 5B | 1.481054688 | 0.001163137 |
| *Noct* | nocturnin | 1.475976563 | 0.001964044 |
| *Osbpl9* | oxysterol binding protein-like 9 | 1.463085938 | 0.001448906 |
| *Rnf19b* | ring finger protein 19B | 1.458007813 | 0.00136927 |
| *Plekhm1* | pleckstrin homology domain containing, family M (with RUN domain) member 1 | 1.457421875 | 0.0060246 |
| *Rfk* | riboflavin kinase | 1.45546875 | 0.003694116 |
| *Derl1* | Der1-like domain family, member 1 | 1.450390625 | 0.003169091 |
| *Akirin1* | akirin 1 | 1.449804688 | 0.001552658 |
| *Ccdc47* | coiled-coil domain containing 47 | 1.447167969 | 0.009481336 |
| *Aasdhppt* | aminoadipate-semialdehyde dehydrogenase-phosphopantetheinyl transferase | 1.446679688 | 0.001675346 |
| *Cops2* | COP9 signalosome subunit 2 | 1.445898438 | 0.000298083 |
| *Rc3h2* | ring finger and CCCH-type zinc finger domains 2 | 1.4453125 | 2.39642E-05 |
| *1700094D03Rik* | RIKEN cDNA 1700094D03 gene | 1.44140625 | 0.001345699 |
| *Smap2* | small ArfGAP 2 | 1.440234375 | 0.000749579 |
| *Uhrf1bp1* | UHRF1 (ICBP90) binding protein 1 | 1.43359375 | 0.005782524 |
| *Bin3* | bridging integrator 3 | 1.43359375 | 0.000601576 |
| *Dapp1* | dual adaptor for phosphotyrosine and 3-phosphoinositides 1 | 1.432617188 | 0.000383993 |
| *Vwa5a* | von Willebrand factor A domain containing 5A | 1.432421875 | 0.044459357 |
| *Rnf20* | ring finger protein 20 | 1.431055949 | 0.019382026 |
| *Mrpl32* | mitochondrial ribosomal protein L32 | 1.429840269 | 0.003836691 |
| *Saraf* | store-operated calcium entry-associated regulatory factor | 1.427929687 | 0.000799213 |
| *Rab5a* | RAB5A, member RAS oncogene family | 1.426171875 | 0.002710398 |
| *Cnppd1* | cyclin Pas1/PHO80 domain containing 1 | 1.424804688 | 0.014454708 |
| *Borcs6* | BLOC-1 related complex subunit 6 | 1.424414063 | 0.002945431 |
| *Dcaf7* | DDB1 and CUL4 associated factor 7 | 1.415429688 | 0.008730326 |
| *Ift20* | intraflagellar transport 20 | 1.41484375 | 0.000176851 |
| *Psmd4* | proteasome (prosome, macropain) 26S subunit, non-ATPase, 4 | 1.41328125 | 0.000902217 |
| *Eif3b* | eukaryotic translation initiation factor 3, subunit B | 1.409375 | 0.002569933 |
| *Ccdc66* | coiled-coil domain containing 66 | 1.407324219 | 0.008162671 |
| *Hsp90ab1* | heat shock protein 90 alpha (cytosolic), class B member 1 | 1.402539063 | 0.00204385 |
| *Psmd10* | proteasome (prosome, macropain) 26S subunit, non-ATPase, 10 | 1.400585938 | 0.000704347 |
| *Nub1* | negative regulator of ubiquitin-like proteins 1 | 1.386914062 | 0.003175507 |
| *Pitrm1* | pitrilysin metallepetidase 1 | 1.3859375 | 5.2084E-05 |
| *Ccdc32* | coiled-coil domain containing 32 | 1.384765625 | 0.001718041 |
| *Wars* | tryptophanyl-tRNA synthetase | 1.382421875 | 0.000203465 |
| *Aip* | aryl-hydrocarbon receptor-interacting protein | 1.374414063 | 0.000601019 |
| *Zfp131* | zinc finger protein 131 | 1.371875 | 0.000450892 |
| *Kmt5c* | lysine methyltransferase 5C | 1.371289063 | 0.018787727 |
| *Stam* | signal transducing adaptor molecule (SH3 domain and ITAM motif) 1 | 1.369726563 | 0.003703852 |
| *Tusc3* | tumor suppressor candidate 3 | 1.368359375 | 0.005439222 |
| *Gdf15* | growth differentiation factor 15 | 1.367578125 | 0.031561212 |
| *Psmc5* | protease (prosome, macropain) 26S subunit, ATPase 5 | 1.36640625 | 0.000389595 |
| *Rhbdd1* | rhomboid domain containing 1 | 1.364453125 | 0.017329205 |
| *Senp6* | SUMO/sentrin specific peptidase 6 | 1.358593749 | 0.0060115 |
| *Tfe3* | transcription factor E3 | 1.356835938 | 0.00515595 |
| *Copz1* | coatomer protein complex, subunit zeta 1 | 1.35625 | 9.50243E-06 |
| *Pfkp* | phosphofructokinase, platelet | 1.355566406 | 0.002040263 |
| *Prrc2b* | proline-rich coiled-coil 2B | 1.353125 | 0.00302012 |
| *Grk6* | G protein-coupled receptor kinase 6 | 1.351757813 | 0.004382751 |
| *Rbm41* | RNA binding motif protein 41 | 1.35078125 | 0.012064831 |
| *Spty2d1* | SPT2, Suppressor of Ty, domain containing 1 (S. cerevisiae) | 1.348046875 | 0.000992977 |
| *Pak6* | p21 protein (Cdc42/Rac)-activated kinase 6 | 1.347558594 | 0.010563137 |
| *Creb3* | cAMP responsive element binding protein 3 | 1.334179688 | 0.011585038 |
| *Aamp* | angio-associated migratory protein | 1.330664063 | 0.001877473 |
| *Pkd1l3* | polycystic kidney disease 1 like 3 | 1.329785156 | 0.021882745 |
| *1110008F13Rik* | RIKEN cDNA 1110008F13 gene | 1.325390625 | 0.001400156 |
| *Etf1* | eukaryotic translation termination factor 1 | 1.324804688 | 0.000506865 |
| *Sike1* | suppressor of IKBKE 1 | 1.32421875 | 0.004283303 |
| *Ndufa7* | NADH dehydrogenase (ubiquinone) 1 alpha subcomplex, 7 (B14.5a) | 1.322460938 | 0.007624995 |
| *Tbck* | TBC1 domain containing kinase | 1.317578125 | 0.018961302 |
| *Timm17a* | translocase of inner mitochondrial membrane 17a | 1.310546875 | 0.002331915 |
| *Atg12* | autophagy related 12 | 1.303515625 | 0.017865933 |
| *Slc2a1* | solute carrier family 2 (facilitated glucose transporter), member 1 | 1.301757813 | 0.001218751 |
| *March7* | membrane-associated ring finger (C3HC4) 7 | 1.298046875 | 0.020094124 |
| *Ctr9* | CTR9 homolog, Paf1/RNA polymerase II complex component | 1.297851563 | 0.011958867 |
| *Ube2a* | ubiquitin-conjugating enzyme E2A | 1.295703125 | 0.000666169 |
| *Por* | P450 (cytochrome) oxidoreductase | 1.294921875 | 0.000910385 |
| *Dctn2* | dynactin 2 | 1.286328125 | 0.004054561 |
| *Zmym5* | zinc finger, MYM-type 5 | 1.284570313 | 0.002074911 |
| *Crkl* | v-crk avian sarcoma virus CT10 oncogene homolog-like | 1.283007812 | 0.000962686 |
| *Ttc1* | tetratricopeptide repeat domain 1 | 1.27734375 | 0.001244979 |
| *Psmb7* | proteasome (prosome, macropain) subunit, beta type 7 | 1.26484375 | 0.000647886 |
| *Tfpt* | TCF3 (E2A) fusion partner | 1.264453125 | 0.002759652 |
| *Ddb1* | damage specific DNA binding protein 1 | 1.263085938 | 0.001531007 |
| *Cyp4f39* | cytochrome P450, family 4, subfamily f, polypeptide 39 | 1.261230469 | 0.014062212 |
| *Ppard* | peroxisome proliferator activator receptor delta | 1.261132814 | 0.005415449 |
| *Trappc11* | trafficking protein particle complex 11 | 1.258007813 | 0.033955424 |
| *Psmd2* | proteasome (prosome, macropain) 26S subunit, non-ATPase, 2 | 1.2578125 | 1.62151E-05 |
| *Trip12* | thyroid hormone receptor interactor 12 | 1.255859375 | 0.000199642 |
| *Ccng2* | cyclin G2 | 1.255273438 | 0.01056167 |
| *Ap2m1* | adaptor-related protein complex 2, mu 1 subunit | 1.252148437 | 0.014767957 |
| *Sgta* | small glutamine-rich tetratricopeptide repeat (TPR)-containing, alpha | 1.250976563 | 0.008550427 |
| *Prps1* | phosphoribosyl pyrophosphate synthetase 1 | 1.242578125 | 0.003161266 |
| *Kmt2d* | lysine (K)-specific methyltransferase 2D | 1.240625 | 2.85831E-05 |
| *Avl9* | AVL9 cell migration associated | 1.240234375 | 0.042906149 |
| *Zfp691* | zinc finger protein 691 | 1.238867188 | 0.011947118 |
| *Mea1* | male enhanced antigen 1 | 1.238085938 | 0.00177436 |
| *Rnh1* | ribonuclease/angiogenin inhibitor 1 | 1.238085938 | 0.024609451 |
| *Ywhag* | tyrosine 3-monooxygenase/tryptophan 5-monooxygenase activation protein, gamma polypeptide | 1.237109375 | 0.002836036 |
| *Trp53* | transformation related protein 53 | 1.235546875 | 0.007118709 |
| *Ubr4* | ubiquitin protein ligase E3 component n-recognin 4 | 1.2296875 | 0.000697935 |
| *Idnk* | idnK gluconokinase homolog (E. coli) | 1.224804688 | 0.027899822 |
| *Ufd1* | ubiquitin recognition factor in ER-associated degradation 1 | 1.223046875 | 0.000575451 |
| *Epg5* | ectopic P-granules autophagy protein 5 homolog (C. elegans) | 1.222070313 | 0.004293654 |
| *Ostc* | oligosaccharyltransferase complex subunit (non-catalytic) | 1.219335938 | 0.034195489 |
| *Uqcrc2* | ubiquinol cytochrome c reductase core protein 2 | 1.219335937 | 0.001069064 |
| *Gpn3* | GPN-loop GTPase 3 | 1.218359375 | 0.000311886 |
| *Col4a3bp* | collagen, type IV, alpha 3 (Goodpasture antigen) binding protein | 1.21796875 | 0.010099939 |
| *Tlk2* | tousled-like kinase 2 (Arabidopsis) | 1.217578125 | 0.004948318 |
| *Dync1li2* | dynein, cytoplasmic 1 light intermediate chain 2 | 1.216601563 | 0.00787583 |
| *Chchd10* | coiled-coil-helix-coiled-coil-helix domain containing 10 | 1.2140625 | 0.004744496 |
| *Upf3a* | UPF3 regulator of nonsense transcripts homolog A (yeast) | 1.213476563 | 0.003295623 |
| *Lancl1* | LanC (bacterial lantibiotic synthetase component C)-like 1 | 1.211914063 | 0.01151046 |
| *Mrps18a* | mitochondrial ribosomal protein S18A | 1.210742188 | 0.00803161 |
| *Sra1* | steroid receptor RNA activator 1 | 1.208203126 | 0.000719601 |
| *Zwint* | ZW10 interactor | 1.208203125 | 0.016066687 |
| *Tmem259* | transmembrane protein 259 | 1.20703125 | 0.000125852 |
| *M6pr* | mannose-6-phosphate receptor, cation dependent | 1.205664063 | 0.001060979 |
| *Gosr2* | golgi SNAP receptor complex member 2 | 1.203710938 | 0.009405419 |
| *Exoc6* | exocyst complex component 6 | 1.201367188 | 1.62151E-05 |
| *Golim4* | golgi integral membrane protein 4 | 1.199023438 | 0.004232799 |
| *Ubr3* | ubiquitin protein ligase E3 component n-recognin 3 | 1.198242188 | 0.000383993 |
| *Atxn3* | ataxin 3 | 1.195898438 | 0.002320439 |
| *Fam3a* | family with sequence similarity 3, member A | 1.195800781 | 0.042179294 |
| *Rnf40* | ring finger protein 40 | 1.194531261 | 0.002893444 |
| *Mef2a* | myocyte enhancer factor 2A | 1.192578125 | 0.007193192 |
| *Ccdc12* | coiled-coil domain containing 12 | 1.1890625 | 0.014558736 |
| *Prdx6* | peroxiredoxin 6 | 1.188867188 | 0.004910191 |
| *Psma2* | proteasome (prosome, macropain) subunit, alpha type 2 | 1.18515625 | 0.000219124 |
| *Elk1* | ELK1, member of ETS oncogene family | 1.18359375 | 0.004071864 |
| *Frg1* | FSHD region gene 1 | 1.18359375 | 0.01481424 |
| *Crcp* | calcitonin gene-related peptide-receptor component protein | 1.1828125 | 0.001479171 |
| *Ss18l2* | SS18, nBAF chromatin remodeling complex subunit like 2 | 1.182617188 | 0.028445923 |
| *Ndufb11* | NADH dehydrogenase (ubiquinone) 1 beta subcomplex, 11 | 1.181054688 | 0.047631422 |
| *Eif4g1* | eukaryotic translation initiation factor 4, gamma 1 | 1.18046875 | 0.002398419 |
| *Gm9774* | adhesion regulating molecule 1 pseudogene | 1.179882813 | 0.001589823 |
| *Psme4* | proteasome (prosome, macropain) activator subunit 4 | 1.177734375 | 0.001663332 |
| *Aldh9a1* | aldehyde dehydrogenase 9, subfamily A1 | 1.17734375 | 0.014131337 |
| *Kdm6b* | KDM1 lysine (K)-specific demethylase 6B | 1.175195311 | 0.005538985 |
| *Eloa* | elongin A | 1.175 | 0.004180307 |
| *Actr10* | ARP10 actin-related protein 10 | 1.172668805 | 2.66957E-05 |
| *Mmadhc* | methylmalonic aciduria (cobalamin deficiency) cblD type, with homocystinuria | 1.172460938 | 0.001517224 |
| *Sirt2* | sirtuin 2 | 1.171136059 | 0.005407101 |
| *Zfp266* | zinc finger protein 266 | 1.170117187 | 0.036400103 |
| *Zfp959* | zinc finger protein 959 | 1.16953125 | 0.003462954 |
| *Mtpn* | myotrophin | 1.165429688 | 0.001166278 |
| *Pim1* | proviral integration site 1 | 1.165234375 | 0.002422324 |
| *Etfrf1* | electron transfer flavoprotein regulatory factor 1 | 1.161816406 | 0.003317641 |
| *Hyal3* | hyaluronoglucosaminidase 3 | 1.16171875 | 0.000156348 |
| *Selenot* | selenoprotein T | 1.159765625 | 2.54387E-05 |
| *Hist2h3b* | histone cluster 2, H3b | 1.154980469 | 0.001027733 |
| *Ddx49* | DEAD (Asp-Glu-Ala-Asp) box polypeptide 49 | 1.154296875 | 0.007745545 |
| *Aimp1* | aminoacyl tRNA synthetase complex-interacting multifunctional protein 1 | 1.1515625 | 0.006239517 |
| *Cope* | coatomer protein complex, subunit epsilon | 1.15078125 | 0.001172128 |
| *Tmem214* | transmembrane protein 214 | 1.15 | 0.027107595 |
| *Gnl3l* | guanine nucleotide binding protein-like 3 (nucleolar)-like | 1.148632813 | 0.004450253 |
| *Imp4* | IMP4, U3 small nucleolar ribonucleoprotein | 1.1484375 | 0.048392836 |
| *Rptor* | regulatory associated protein of MTOR, complex 1 | 1.1484375 | 0.003703435 |
| *Ginm1* | glycoprotein integral membrane 1 | 1.14609375 | 0.017041734 |
| *Vps11* | VPS11, CORVET/HOPS core subunit | 1.1453125 | 0.009343308 |
| *Ncor1* | nuclear receptor co-repressor 1 | 1.144921875 | 4.62829E-05 |
| *Trove2* | TROVE domain family, member 2 | 1.1390625 | 0.012112199 |
| *Kmt5b* | lysine methyltransferase 5B | 1.138476561 | 0.000649846 |
| *Ofd1* | OFD1, centriole and centriolar satellite protein | 1.132519531 | 0.023213819 |
| *Atp7a* | ATPase, Cu++ transporting, alpha polypeptide | 1.128320313 | 0.004471216 |
| *Cops5* | COP9 signalosome subunit 5 | 1.1265625 | 0.003024427 |
| *Anapc16* | anaphase promoting complex subunit 16 | 1.124609375 | 3.35226E-05 |
| *Calcoco1* | calcium binding and coiled coil domain 1 | 1.12421875 | 0.00339948 |
| *Farsb* | phenylalanyl-tRNA synthetase, beta subunit | 1.123632813 | 0.00097339 |
| *Cd2ap* | CD2-associated protein | 1.123632813 | 0.00417012 |
| *Larp1* | La ribonucleoprotein domain family, member 1 | 1.12265625 | 0.000979253 |
| *Ndel1* | nudE neurodevelopment protein 1 like 1 | 1.121289063 | 0.049671705 |
| *Zyg11b* | zyg-ll family member B, cell cycle regulator | 1.119824219 | 0.011337976 |
| *Upf1* | UPF1 regulator of nonsense transcripts homolog (yeast) | 1.119140625 | 0.012616218 |
| *Rbm33* | RNA binding motif protein 33 | 1.116992188 | 0.019568853 |
| *Rfesd* | Rieske (Fe-S) domain containing | 1.11484375 | 0.001107887 |
| *Eipr1* | EARP complex and GARP complex interacting protein 1 | 1.112109375 | 0.01678729 |
| *Mknk1* | MAP kinase-interacting serine/threonine kinase 1 | 1.111523438 | 0.008980202 |
| *Ube2j1* | ubiquitin-conjugating enzyme E2J 1 | 1.111328124 | 0.018678404 |
| *Uck2* | uridine-cytidine kinase 2 | 1.109375 | 0.004860195 |
| *Psenen* | presenilin enhancer gamma secretase subunit | 1.107421875 | 0.000984301 |
| *Aff4* | AF4/FMR2 family, member 4 | 1.103710938 | 0.019279171 |
| *Dera* | deoxyribose-phosphate aldolase (putative) | 1.103710938 | 0.042499104 |
| *Cds2* | CDP-diacylglycerol synthase (phosphatidate cytidylyltransferase) 2 | 1.101367188 | 0.043667368 |
| *Nsun2* | NOL1/NOP2/Sun domain family member 2 | 1.101171875 | 0.013951889 |
| *Supt6* | suppressor of Ty 6 | 1.101171806 | 0.000255436 |
| *Chordc1* | cysteine and histidine-rich domain (CHORD)-containing, zinc-binding protein 1 | 1.100976562 | 0.03351325 |
| *Xpot* | exportin, tRNA (nuclear export receptor for tRNAs) | 1.099316406 | 0.015034141 |
| *Chmp1a* | charged multivesicular body protein 1A | 1.093554687 | 0.014966026 |
| *Mthfd2* | methylenetetrahydrofolate dehydrogenase (NAD+ dependent), methenyltetrahydrofolate cyclohydrolase | 1.091601563 | 0.040135614 |
| *Ammecr1l* | AMME chromosomal region gene 1-like | 1.088476563 | 0.012617806 |
| *Bach1* | BTB and CNC homology 1, basic leucine zipper transcription factor 1 | 1.082617188 | 0.004366328 |
| *Dpf2* | D4, zinc and double PHD fingers family 2 | 1.081835937 | 0.000650279 |
| *Psmb6* | proteasome (prosome, macropain) subunit, beta type 6 | 1.080078125 | 0.001365018 |
| *Arhgap17* | Rho GTPase activating protein 17 | 1.077929688 | 0.007332123 |
| *Tmem167b* | transmembrane protein 167B | 1.075390625 | 0.00087362 |
| *Bbc3* | BCL2 binding component 3 | 1.074609375 | 0.028549337 |
| *Prcc* | papillary renal cell carcinoma (translocation-associated) | 1.074023438 | 0.031806426 |
| *Psmd3* | proteasome (prosome, macropain) 26S subunit, non-ATPase, 3 | 1.074023438 | 0.049376149 |
| *Xpnpep1* | X-prolyl aminopeptidase (aminopeptidase P) 1, soluble | 1.072851563 | 0.027152945 |
| *Higd2a* | HIG1 domain family, member 2A | 1.0703125 | 0.002036648 |
| *Efhd2* | EF hand domain containing 2 | 1.069726562 | 0.005534554 |
| *Psmc3* | proteasome (prosome, macropain) 26S subunit, ATPase 3 | 1.069140625 | 0.000441311 |
| *Snd1* | staphylococcal nuclease and tudor domain containing 1 | 1.068359375 | 0.008550427 |
| *Kpna6* | karyopherin (importin) alpha 6 | 1.066601559 | 0.007206414 |
| *Rmdn3* | regulator of microtubule dynamics 3 | 1.066015625 | 0.011958054 |
| *Bcl10* | B cell leukemia/lymphoma 10 | 1.064257813 | 0.017337401 |
| *Tbrg1* | transforming growth factor beta regulated gene 1 | 1.060742188 | 0.028070759 |
| *Slc52a2* | solute carrier protein 52, member 2 | 1.060253906 | 0.000572239 |
| *Mtr* | 5-methyltetrahydrofolate-homocysteine methyltransferase | 1.06015625 | 0.006199205 |
| *Anxa7* | annexin A7 | 1.059960938 | 3.97526E-05 |
| *Mat2b* | methionine adenosyltransferase II, beta | 1.058789063 | 0.036320718 |
| *Osm* | oncostatin M | 1.055664063 | 0.001022325 |
| *Acox1* | acyl-Coenzyme A oxidase 1, palmitoyl | 1.055566406 | 0.000602639 |
| *Abca4* | ATP-binding cassette, sub-family A (ABC1), member 4 | 1.053222656 | 0.008488476 |
| *Ahsa1* | AHA1, activator of heat shock protein ATPase 1 | 1.050597658 | 0.042023516 |
| *Fars2* | phenylalanine-tRNA synthetase 2 (mitochondrial) | 1.050390625 | 0.038752475 |
| *Ankrd44* | ankyrin repeat domain 44 | 1.049902344 | 0.030576024 |
| *Nop10* | NOP10 ribonucleoprotein | 1.04921875 | 0.048716878 |
| *Gm10709* | ribosomal protein L29 pseudogene | 1.047265625 | 0.006572273 |
| *Sqstm1* | sequestosome 1 | 1.044921875 | 0.001425 |
| *Eci1* | enoyl-Coenzyme A delta isomerase 1 | 1.044726563 | 0.003191328 |
| *Mnat1* | menage a trois 1 | 1.044628906 | 0.006980791 |
| *Mdm2* | transformed mouse 3T3 cell double minute 2 | 1.04296875 | 0.010959143 |
| *Nek7* | NIMA (never in mitosis gene a)-related expressed kinase 7 | 1.04140625 | 0.007122151 |
| *Nfkbib* | nuclear factor of kappa light polypeptide gene enhancer in B cells inhibitor, beta | 1.03984375 | 0.002655417 |
| *Tmem242* | transmembrane protein 242 | 1.034960938 | 0.0447484 |
| *Arih2* | ariadne RBR E3 ubiquitin protein ligase 2 | 1.034426428 | 0.00019 |
| *Slc1a4* | solute carrier family 1 (glutamate/neutral amino acid transporter), member 4 | 1.034375 | 0.000510265 |
| *LOC102633809* | guanine nucleotide-binding protein G(I)/G(S)/G(O) subunit gamma-5 pseudogene | 1.033984375 | 0.029833509 |
| *Pex3* | peroxisomal biogenesis factor 3 | 1.030078125 | 0.007556502 |
| *Uba3* | ubiquitin-like modifier activating enzyme 3 | 1.029492187 | 0.011552041 |
| *Snx1* | sorting nexin 1 | 1.029101563 | 0.026303943 |
| *Treml2* | triggering receptor expressed on myeloid cells-like 2 | 1.027929688 | 0.024410782 |
| *Naa25* | N(alpha)-acetyltransferase 25, NatB auxiliary subunit | 1.026464844 | 0.000139114 |
| *Drap1* | Dr1 associated protein 1 (negative cofactor 2 alpha) | 1.02578125 | 0.040301545 |
| *Zscan25* | zinc finger and SCAN domain containing 25 | 1.025 | 0.005119564 |
| *Tceanc2* | transcription elongation factor A (SII) N-terminal and central domain containing 2 | 1.024023438 | 0.021123924 |
| *Uqcr10* | ubiquinol-cytochrome c reductase, complex III subunit X | 1.022265625 | 0.000274185 |
| *Mgea5* | meningioma expressed antigen 5 (hyaluronidase) | 1.022265625 | 0.003536639 |
| *Klhdc10* | kelch domain containing 10 | 1.021289063 | 0.003901882 |
| *Gm40346* | predicted gene, 40346 | 1.021191406 | 0.017361398 |
| *D11Wsu47e* | DNA segment, Chr 11, Wayne State University 47, expressed | 1.020898438 | 0.010171547 |
| *Gm8394* | proteasome zeta chain | 1.019921875 | 0.001564741 |
| *Bap1* | Brca1 associated protein 1 | 1.018945313 | 0.001915746 |
| *Chm* | choroidermia (RAB escort protein 1) | 1.018652344 | 0.043590595 |
| *Dync1li1* | dynein cytoplasmic 1 light intermediate chain 1 | 1.016796875 | 0.000566942 |
| *Rlim* | ring finger protein, LIM domain interacting | 1.015625 | 0.004163663 |
| *Txndc17* | thioredoxin domain containing 17 | 1.015380395 | 0.015719452 |
| *Psmc6* | proteasome (prosome, macropain) 26S subunit, ATPase, 6 | 1.0140625 | 0.000753896 |
| *Psmc2* | proteasome (prosome, macropain) 26S subunit, ATPase 2 | 1.013672038 | 7.03733E-05 |
| *Blmh* | bleomycin hydrolase | 1.0125 | 0.010846224 |
| *Calr* | calreticulin | 1.01015625 | 4.26296E-05 |
| *Rpl27-ps1* | ribosomal protein L27, pseudogene 1 | 1.01015625 | 0.045385009 |
| *Eif4g3* | eukaryotic translation initiation factor 4 gamma, 3 | 1.009373756 | 0.003720673 |
| *Rnf115* | ring finger protein 115 | 1.006640625 | 0.02856401 |
| *Dedd2* | death effector domain-containing DNA binding protein 2 | 1.006445312 | 0.037826704 |
| *Gpr150* | G protein-coupled receptor 150 | 1.00390625 | 0.003212046 |
| *Mindy1* | MINDY lysine 48 deubiquitinase 1 | 1.00390625 | 0.003536639 |
| *Bag3* | BCL2-associated athanogene 3 | 1.003125 | 0.007468987 |
| *Trp53bp1* | transformation related protein 53 binding protein 1 | 1.003125 | 0.003265528 |
| *Gnb1* | guanine nucleotide binding protein (G protein), beta 1 | 1.000976563 | 0.001358111 |
| *Hnrnpa2b1* | heterogeneous nuclear ribonucleoprotein A2/B1 | -1.0015625 | 0.010829818 |
| *Knstrn* | kinetochore-localized astrin/SPAG5 binding | -1.00234375 | 0.003538392 |
| *Srsf3* | serine/arginine-rich splicing factor 3 | -1.003125 | 0.019515407 |
| *Mettl7a3* | methyltransferase like 7A3 | -1.004785156 | 0.021339926 |
| *Gins2* | GINS complex subunit 2 (Psf2 homolog) | -1.008007813 | 0.035020632 |
| *Slc2a4* | solute carrier family 2 (facilitated glucose transporter), member 4 | -1.01015625 | 0.044295077 |
| *Rbm44* | RNA binding motif protein 44 | -1.010449219 | 0.037292131 |
| *Gba2* | glucosidase beta 2 | -1.013476563 | 0.030969737 |
| *Tnk2* | tyrosine kinase, non-receptor, 2 | -1.013476563 | 0.00413466 |
| *Ap4s1* | adaptor-related protein complex AP-4, sigma 1 | -1.013476563 | 0.019619236 |
| *Rnase6* | ribonuclease, RNase A family, 6 | -1.014746094 | 1.62151E-05 |
| *Brd3* | bromodomain containing 3 | -1.016210938 | 0.000996568 |
| *Tap1* | transporter 1, ATP-binding cassette, sub-family B (MDR/TAP) | -1.018652344 | 0.003600432 |
| *Tmed10* | transmembrane p24 trafficking protein 10 | -1.018945312 | 0.0060115 |
| *Tti1* | TELO2 interacting protein 1 | -1.019824219 | 0.045804934 |
| *Wdfy3* | WD repeat and FYVE domain containing 3 | -1.02109375 | 0.000175471 |
| *Fads3* | fatty acid desaturase 3 | -1.021872804 | 0.025700224 |
| *Tm7sf3* | transmembrane 7 superfamily member 3 | -1.022460938 | 0.020782626 |
| *Slf2* | SMC5-SMC6 complex localization factor 2 | -1.026269531 | 0.000310113 |
| *Nrm* | nurim (nuclear envelope membrane protein) | -1.026367188 | 0.031226735 |
| *Mir669n* | microRNA 669n | -1.028613281 | 0.049186271 |
| *LOC105246710* | uncharacterized LOC105246710 | -1.029296875 | 0.023605108 |
| *Mcu* | mitochondrial calcium uniporter | -1.029394531 | 0.042782915 |
| *Gga2* | golgi associated, gamma adaptin ear containing, ARF binding protein 2 | -1.029785156 | 0.016498006 |
| *9230116N13Rik* | RIKEN cDNA 9230116N13 gene | -1.030566406 | 0.000596328 |
| *2310033P09Rik* | RIKEN cDNA 2310033P09 gene | -1.035742187 | 0.007073464 |
| *Prkdc* | protein kinase, DNA activated, catalytic polypeptide | -1.036914063 | 0.000280317 |
| *Suz12* | SUZ12 polycomb repressive complex 2 subunit | -1.03828125 | 0.007751968 |
| *Atrx* | ATRX, chromatin remodeler | -1.038671875 | 0.011279688 |
| *Mylip* | myosin regulatory light chain interacting protein | -1.044140625 | 0.04926635 |
| *Slc35g1* | solute carrier family 35, member G1 | -1.044433594 | 0.039820667 |
| *Cdkn2d* | cyclin-dependent kinase inhibitor 2D (p19, inhibits CDK4) | -1.04453125 | 0.009552292 |
| *Steap3* | STEAP family member 3 | -1.044726566 | 0.025240667 |
| *Ssx2ip* | synovial sarcoma, X 2 interacting protein | -1.044921875 | 0.01202957 |
| *Stard10* | START domain containing 10 | -1.044921875 | 0.003085996 |
| *Erlec1* | endoplasmic reticulum lectin 1 | -1.045898438 | 0.00887589 |
| *H2afy* | H2A histone family, member Y | -1.046875 | 0.042593174 |
| *Tecr* | trans-2,3-enoyl-CoA reductase | -1.047265625 | 0.010474458 |
| *Tcerg1* | transcription elongation regulator 1 (CA150) | -1.050195313 | 0.000600966 |
| *Ran* | RAN, member RAS oncogene family | -1.051367188 | 0.036765118 |
| *Cmc4* | C-x(9)-C motif containing 4 | -1.0515625 | 0.000625402 |
| *Klf10* | Kruppel-like factor 10 | -1.052246094 | 0.036320718 |
| *Fanci* | Fanconi anemia, complementation group I | -1.055078125 | 0.00455158 |
| *Mtrf1l* | mitochondrial translational release factor 1-like | -1.058886719 | 0.017391681 |
| *Phf2* | PHD finger protein 2 | -1.059765625 | 0.031854683 |
| *Snord12* | small nucleolar RNA, C/D box 12 | -1.060742188 | 0.020388617 |
| *Mbd4* | methyl-CpG binding domain protein 4 | -1.063574219 | 0.003855589 |
| *Rnf216* | ring finger protein 216 | -1.067578125 | 0.000302998 |
| *Dnajb11* | DnaJ heat shock protein family (Hsp40) member B11 | -1.071484375 | 0.039281233 |
| *Slc44a1* | solute carrier family 44, member 1 | -1.072460938 | 0.002568478 |
| *Selplg* | selectin, platelet (p-selectin) ligand | -1.072851563 | 0.024668268 |
| *Asap1* | ArfGAP with SH3 domain, ankyrin repeat and PH domain1 | -1.0734375 | 0.001826224 |
| *Hist1h2bb* | histone cluster 1, H2bb | -1.075 | 0.018319614 |
| *Cdca2* | cell division cycle associated 2 | -1.076953125 | 0.01576519 |
| *Akap7* | A kinase (PRKA) anchor protein 7 | -1.078126485 | 0.007649981 |
| *Smarcc1* | SWI/SNF related, matrix associated, actin dependent regulator of chromatin, subfamily c, member 1 | -1.078710938 | 0.027581496 |
| *Actn4* | actinin alpha 4 | -1.079101563 | 0.028395974 |
| *Lsm8* | LSM8 homolog, U6 small nuclear RNA associated | -1.080859375 | 0.018956675 |
| *Dot1l* | DOT1-like, histone H3 methyltransferase (S. cerevisiae) | -1.081054688 | 0.047640162 |
| *C530008M17Rik* | RIKEN cDNA C530008M17 gene | -1.08125 | 0.012130937 |
| *Snord61* | small nucleolar RNA, C/D box 61 | -1.082617188 | 0.00208353 |
| *Pcgf5* | polycomb group ring finger 5 | -1.084765625 | 0.003234306 |
| *E2f3* | E2F transcription factor 3 | -1.086621094 | 0.00169 |
| *Ccna2* | cyclin A2 | -1.0875 | 0.010382086 |
| *Cetn3* | centrin 3 | -1.089453125 | 0.035624343 |
| *Pnpla7* | patatin-like phospholipase domain containing 7 | -1.090917969 | 0.000689365 |
| *Aim2* | absent in melanoma 2 | -1.09296875 | 0.022811415 |
| *Gm8741* | Nhp2 non-histone chromosome protein 2-like 1 pseudogene | -1.09296875 | 0.007633327 |
| *Wdr90* | WD repeat domain 90 | -1.096582031 | 0.000533083 |
| *Notch2* | notch 2 | -1.097265625 | 0.010142906 |
| *Gatad1* | GATA zinc finger domain containing 1 | -1.097851563 | 0.037470124 |
| *Psip1* | PC4 and SFRS1 interacting protein 1 | -1.099121094 | 0.005168929 |
| *D730005E14Rik* | RIKEN cDNA D730005E14 gene | -1.100097656 | 0.040882913 |
| *Hnrnpu* | heterogeneous nuclear ribonucleoprotein U | -1.101171875 | 0.001071527 |
| *Espl1* | extra spindle pole bodies 1, separase | -1.103150137 | 0.009752978 |
| *Gm550* | predicted gene 550 | -1.103710938 | 0.000561316 |
| *Ttk* | Ttk protein kinase | -1.104199219 | 0.026877777 |
| *Purb* | purine rich element binding protein B | -1.106835938 | 0.026748437 |
| *Hmgb1-ps7* | high-mobility group high mobility group box 1, pseudogene 7 | -1.1078125 | 0.043871737 |
| *Ntn4* | netrin 4 | -1.109570313 | 0.037371247 |
| *Ccdc34* | coiled-coil domain containing 34 | -1.109570313 | 0.016946965 |
| *Zmiz1* | zinc finger, MIZ-type containing 1 | -1.110351563 | 0.002365939 |
| *Bex3* | brain expressed X-linked 3 | -1.111132813 | 0.013512221 |
| *Pml* | promyelocytic leukemia | -1.11171875 | 0.003422492 |
| *Taf6* | TATA-box binding protein associated factor 6 | -1.112402344 | 0.041001161 |
| *Ranbp1* | RAN binding protein 1 | -1.112890625 | 0.040569223 |
| *Trim30a* | tripartite motif-containing 30A | -1.113085938 | 0.001974649 |
| *Sun1* | Sad1 and UNC84 domain containing 1 | -1.1140625 | 0.009998513 |
| *Aurka* | aurora kinase A | -1.1140625 | 0.003803218 |
| *Ano6* | anoctamin 6 | -1.114648436 | 0.004191078 |
| *Eed* | embryonic ectoderm development | -1.115039063 | 0.010516332 |
| *Syce2* | synaptonemal complex central element protein 2 | -1.116015625 | 0.008543674 |
| *Ckap2l* | cytoskeleton associated protein 2-like | -1.11640625 | 0.016441918 |
| *Asap2* | ArfGAP with SH3 domain, ankyrin repeat and PH domain 2 | -1.116992188 | 0.013297805 |
| *LOC102640775* | cyclin-dependent kinases regulatory subunit 1 pseudogene | -1.119726563 | 0.005953417 |
| *Smc5* | structural maintenance of chromosomes 5 | -1.121679688 | 0.022311412 |
| *Usp48* | ubiquitin specific peptidase 48 | -1.123242187 | 0.006195631 |
| *Polr2j* | polymerase (RNA) II (DNA directed) polypeptide J | -1.126953125 | 0.022045477 |
| *Ska1* | spindle and kinetochore associated complex subunit 1 | -1.129101563 | 0.045046977 |
| *Igf1r* | insulin-like growth factor I receptor | -1.130078125 | 2.48408E-05 |
| *Tardbp* | TAR DNA binding protein | -1.131445313 | 0.043662622 |
| *Primpol* | primase and polymerase (DNA-directed) | -1.1328125 | 0.004133659 |
| *Sgo1* | shugoshin 1 | -1.133007813 | 0.027856107 |
| *Gm6745* | predicted gene 6745 | -1.136914063 | 0.030581311 |
| *Chtf18* | CTF18, chromosome transmission fidelity factor 18 | -1.137304688 | 0.003424012 |
| *Phtf1os* | putative homeodomain transcription factor 1, opposite strand | -1.138867188 | 0.001071569 |
| *Gm15290* | predicted gene 15290 | -1.138964844 | 0.002230234 |
| *Psmc3ip* | proteasome (prosome, macropain) 26S subunit, ATPase 3, interacting protein | -1.139160156 | 0.039392356 |
| *Nptn* | neuroplastin | -1.139257813 | 0.00041935 |
| *Spag5* | sperm associated antigen 5 | -1.140820313 | 0.043877765 |
| *6030458C11Rik* | RIKEN cDNA 6030458C11 gene | -1.1453125 | 0.041447151 |
| *Foxd2os* | forkhead box D2, opposite strand | -1.146386719 | 0.032594077 |
| *P4hb* | prolyl 4-hydroxylase, beta polypeptide | -1.147949219 | 0.042779606 |
| *Fam173a* | family with sequence similarity 173, member A | -1.149023438 | 0.010634591 |
| *Gm6669* | glyceraldehyde-3-phosphate dehydrogenase pseudogene | -1.149609375 | 0.032639331 |
| *Cep135* | centrosomal protein 135 | -1.149707031 | 0.041268133 |
| *Vamp5* | vesicle-associated membrane protein 5 | -1.15 | 0.000577552 |
| *Kctd6* | potassium channel tetramerisation domain containing 6 | -1.152441406 | 0.002711178 |
| *Cherp* | calcium homeostasis endoplasmic reticulum protein | -1.15625 | 0.000808866 |
| *Rcc1* | regulator of chromosome condensation 1 | -1.15703125 | 0.007081536 |
| *Ankrd54* | ankyrin repeat domain 54 | -1.158007813 | 0.003966691 |
| *Cep295* | centrosomal protein 295 | -1.159375 | 0.040014753 |
| *Rell1* | RELT-like 1 | -1.159570313 | 0.013630084 |
| *Nfya* | nuclear transcription factor-Y alpha | -1.160546875 | 0.011198773 |
| *C2cd3* | C2 calcium-dependent domain containing 3 | -1.161523438 | 0.029985232 |
| *Bub1b* | BUB1B, mitotic checkpoint serine/threonine kinase | -1.162695313 | 0.008161723 |
| *Fam120a* | family with sequence similarity 120, member A | -1.165535354 | 0.008789688 |
| *Suv39h2* | suppressor of variegation 3-9 2 | -1.167089844 | 0.002108644 |
| *Xpo1* | exportin 1 | -1.16796875 | 0.017219857 |
| *Nap1l4* | nucleosome assembly protein 1-like 4 | -1.168819924 | 0.000789929 |
| *Rcbtb1* | regulator of chromosome condensation (RCC1) and BTB (POZ) domain containing protein 1 | -1.169628906 | 0.001080297 |
| *Kif23* | kinesin family member 23 | -1.169921875 | 0.014881066 |
| *Emc8* | ER membrane protein complex subunit 8 | -1.170703125 | 0.009312187 |
| *Mcm10* | minichromosome maintenance 10 replication initiation factor | -1.176757813 | 0.005156469 |
| *Ipo9* | importin 9 | -1.18125 | 0.014144982 |
| *Dpf3* | D4, zinc and double PHD fingers, family 3 | -1.182226563 | 8.86075E-05 |
| *Rbm4b* | RNA binding motif protein 4B | -1.184277344 | 0.019339349 |
| *Slc20a2* | solute carrier family 20, member 2 | -1.18671875 | 0.001581199 |
| *Sppl2b* | signal peptide peptidase like 2B | -1.18671875 | 0.003524405 |
| *Smc4* | structural maintenance of chromosomes 4 | -1.187109375 | 0.00064927 |
| *Mrpl49* | mitochondrial ribosomal protein L49 | -1.188476563 | 0.033990185 |
| *B230219D22Rik* | RIKEN cDNA B230219D22 gene | -1.189648438 | 0.003614909 |
| *Gpr146* | G protein-coupled receptor 146 | -1.190039062 | 0.002742567 |
| *Tonsl* | tonsoku-like, DNA repair protein | -1.194335938 | 0.002205784 |
| *Ccp110* | centriolar coiled coil protein 110 | -1.195507813 | 0.012888806 |
| *Gm16675* | predicted gene, 16675 | -1.198730469 | 0.00017832 |
| *Hemgn* | hemogen | -1.19921875 | 0.044191022 |
| *Sass6* | SAS-6 centriolar assembly protein | -1.20078125 | 0.001134397 |
| *Stub1* | STIP1 homology and U-Box containing protein 1 | -1.200976563 | 0.007329774 |
| *Olfr67* | olfactory receptor 67 | -1.201074219 | 0.004007024 |
| *Hist1h1d* | histone cluster 1, H1d | -1.204296875 | 0.00797234 |
| *Paxip1* | PAX interacting (with transcription-activation domain) protein 1 | -1.2078125 | 0.013987858 |
| *Akap8* | A kinase (PRKA) anchor protein 8 | -1.209277344 | 0.019325943 |
| *Rbbp8* | retinoblastoma binding protein 8, endonuclease | -1.209570313 | 0.011564176 |
| *Pank4* | pantothenate kinase 4 | -1.211523438 | 0.001387875 |
| *Lbr* | lamin B receptor | -1.212695312 | 0.02787989 |
| *Fbxo48* | F-box protein 48 | -1.215234375 | 0.005289589 |
| *Gm5620* | tubulin, alpha 1B pseudogene | -1.216796875 | 0.002001845 |
| *Spcs3* | signal peptidase complex subunit 3 homolog (S. cerevisiae) | -1.217285156 | 0.005644811 |
| *1110034G24Rik* | RIKEN cDNA 1110034G24 gene | -1.217382813 | 0.002489994 |
| *Cep152* | centrosomal protein 152 | -1.219921875 | 0.000510702 |
| *Nfx1* | nuclear transcription factor, X-box binding 1 | -1.221386719 | 0.00860226 |
| *Ggnbp2os* | gametogenetin binding protein 2, opposite strand | -1.222460938 | 0.005414019 |
| *Dxo* | decapping exoribonuclease | -1.223242188 | 0.000902908 |
| *Hjurp* | Holliday junction recognition protein | -1.2234375 | 0.033840066 |
| *Timeless* | timeless circadian clock 1 | -1.223730469 | 0.000850378 |
| *Gm39590* | predicted gene, 39590 | -1.224121094 | 0.000523335 |
| *Nans* | N-acetylneuraminic acid synthase (sialic acid synthase) | -1.224804688 | 0.015575229 |
| *Tm9sf2* | transmembrane 9 superfamily member 2 | -1.2328125 | 0.038749891 |
| *Cep70* | centrosomal protein 70 | -1.233203125 | 0.011603716 |
| *Nop56* | NOP56 ribonucleoprotein | -1.233398438 | 0.018942435 |
| *Mfhas1* | malignant fibrous histiocytoma amplified sequence 1 | -1.234375 | 6.05488E-06 |
| *Orc5* | origin recognition complex, subunit 5 | -1.235253906 | 0.001017435 |
| *Mzt1* | mitotic spindle organizing protein 1 | -1.235546875 | 0.001324712 |
| *Irf2* | interferon regulatory factor 2 | -1.237109375 | 0.011735056 |
| *Mybl1* | myeloblastosis oncogene-like 1 | -1.237988281 | 0.028326059 |
| *Vrk1* | vaccinia related kinase 1 | -1.238671875 | 0.00536155 |
| *Pagr1a* | PAXIP1 associated glutamate rich protein 1A | -1.241015625 | 0.006058734 |
| *Cdc42ep3* | CDC42 effector protein (Rho GTPase binding) 3 | -1.241015625 | 0.025124678 |
| *Bcl2l11* | BCL2-like 11 (apoptosis facilitator) | -1.242675781 | 0.000209646 |
| *Mapk3* | mitogen-activated protein kinase 3 | -1.242773438 | 0.006010321 |
| *Wee1* | WEE 1 homolog 1 (S. pombe) | -1.243847656 | 0.014106685 |
| *Tubg1* | tubulin, gamma 1 | -1.2453125 | 0.021641869 |
| *Zfp955b* | zinc finger protein 955B | -1.246875 | 0.037146409 |
| *Cklf* | chemokine-like factor | -1.24765625 | 0.014835326 |
| *Dguok* | deoxyguanosine kinase | -1.24983156 | 4.80374E-05 |
| *Amd1* | S-adenosylmethionine decarboxylase 1 | -1.251171875 | 0.035321958 |
| *Actl6a* | actin-like 6A | -1.251464844 | 0.047715001 |
| *Srrm1* | serine/arginine repetitive matrix 1 | -1.253320313 | 0.017365129 |
| *Gse1* | genetic suppressor element 1, coiled-coil protein | -1.257421875 | 0.001631383 |
| *Sfmbt1* | Scm-like with four mbt domains 1 | -1.261914063 | 0.001003948 |
| *Ankle1* | ankyrin repeat and LEM domain containing 1 | -1.26484375 | 0.000778238 |
| *Dnase1l1* | deoxyribonuclease 1-like 1 | -1.265625 | 0.038535779 |
| *Gnai2* | guanine nucleotide binding protein (G protein), alpha inhibiting 2 | -1.266992188 | 0.002336514 |
| *Pot1b* | protection of telomeres 1B | -1.270605469 | 0.005854216 |
| *Cenpf* | centromere protein F | -1.272265625 | 0.004663569 |
| *Trmt2a* | TRM2 tRNA methyltransferase 2A | -1.273828125 | 0.009722372 |
| *Adck5* | aarF domain containing kinase 5 | -1.2765625 | 0.014137059 |
| *Cd36* | CD36 molecule | -1.279296875 | 4.26296E-05 |
| *Tubgcp3* | tubulin, gamma complex associated protein 3 | -1.280078125 | 0.024446413 |
| *BC030867* | cDNA sequence BC030867 | -1.281640625 | 0.006674327 |
| *9530026F06Rik* | RIKEN cDNA 9530026F06 gene | -1.288671875 | 0.006859723 |
| *Pgp* | phosphoglycolate phosphatase | -1.289648438 | 0.043759282 |
| *Dnase2a* | deoxyribonuclease II alpha | -1.28984375 | 0.006617518 |
| *Kras* | Kirsten rat sarcoma viral oncogene homolog | -1.290820313 | 0.025700224 |
| *Tet3* | tet methylcytosine dioxygenase 3 | -1.291992187 | 0.03282244 |
| *Ppp4r3a* | protein phosphatase 4 regulatory subunit 3A | -1.2921875 | 0.027987949 |
| *Ammecr1* | Alport syndrome, mental retardation, midface hypoplasia and elliptocytosis chromosomal region gene 1 | -1.2921875 | 0.025314717 |
| *Hmgb1* | high mobility group box 1 | -1.298828125 | 0.000913598 |
| *Gm6104* | high mobility group box 3 pseudogene | -1.300097656 | 0.021158058 |
| *2610020C07Rik* | RIKEN cDNA 2610020C07 gene | -1.302832031 | 0.008624304 |
| *Mcm9* | minichromosome maintenance 9 homologous recombination repair factor | -1.303027344 | 0.00398812 |
| *Gm15421* | ribosomal protein L22 like 1 pseudogene | -1.303808594 | 0.000318873 |
| *Mettl9* | methyltransferase like 9 | -1.305761719 | 0.00110393 |
| *4930579G24Rik* | RIKEN cDNA 4930579G24 gene | -1.307875788 | 0.021514821 |
| *Pold2* | polymerase (DNA directed), delta 2, regulatory subunit | -1.30859375 | 0.025580228 |
| *Fam122a* | family with sequence similarity 122, member A | -1.310742187 | 0.044927038 |
| *Tdp2* | tyrosyl-DNA phosphodiesterase 2 | -1.310839844 | 0.026990447 |
| *4833417C18Rik* | RIKEN cDNA 4833417C18 gene | -1.311230469 | 0.000163839 |
| *Helq* | helicase, POLQ-like | -1.317089844 | 0.010869033 |
| *Rnaseh2b* | ribonuclease H2, subunit B | -1.321289063 | 0.001021736 |
| *Nek2* | NIMA (never in mitosis gene a)-related expressed kinase 2 | -1.321875 | 0.00752692 |
| *Gch1* | GTP cyclohydrolase 1 | -1.32265625 | 1.69222E-05 |
| *Fam45a* | family with sequence similarity 45, member A | -1.323828125 | 0.031055133 |
| *Trmo* | tRNA methyltransferase O | -1.324804688 | 0.008346953 |
| *Msh2* | mutS homolog 2 | -1.329003906 | 0.001471831 |
| *Ccdc82* | coiled-coil domain containing 82 | -1.329492188 | 0.020515083 |
| *Haus4* | HAUS augmin-like complex, subunit 4 | -1.331384324 | 6.88489E-05 |
| *Gm10282* | high mobility group nucleosomal binding domain 2 pseudogene | -1.332910156 | 0.036943629 |
| *Ache* | acetylcholinesterase | -1.333984375 | 0.002461047 |
| *B2m* | beta-2 microglobulin | -1.335546875 | 0.000415025 |
| *Tmc3* | transmembrane channel-like gene family 3 | -1.336816406 | 0.000263271 |
| *Snrnp40* | small nuclear ribonucleoprotein 40 (U5) | -1.341210938 | 0.006446249 |
| *Rad54b* | RAD54 homolog B (S. cerevisiae) | -1.341699219 | 0.00104996 |
| *Ermp1* | endoplasmic reticulum metallopeptidase 1 | -1.342578125 | 0.002043068 |
| *Bcas3os1* | breast carcinoma amplified sequence 3, opposite strand 1 | -1.34296875 | 0.000956325 |
| *Ect2* | ect2 oncogene | -1.34440322 | 0.036144203 |
| *9130221H12Rik* | RIKEN cDNA 9130221H12 gene | -1.345703125 | 0.002320663 |
| *Zw10* | zw10 kinetochore protein | -1.349804688 | 0.009382368 |
| *Me2* | malic enzyme 2, NAD(+)-dependent, mitochondrial | -1.35029297 | 0.000543088 |
| *Foxm1* | forkhead box M1 | -1.352539062 | 0.015664505 |
| *Kif24* | kinesin family member 24 | -1.353906606 | 0.001478534 |
| *Cenpi* | centromere protein I | -1.355664062 | 0.012704674 |
| *Fn3k* | fructosamine 3 kinase | -1.355664063 | 0.047021115 |
| *Shcbp1* | Shc SH2-domain binding protein 1 | -1.358105469 | 0.031544081 |
| *Olfr68* | olfactory receptor 68 | -1.358496094 | 0.006578766 |
| *Pnn* | pinin | -1.36171875 | 0.014300832 |
| *Ezr* | ezrin | -1.364648438 | 0.002992816 |
| *Fbxw2* | F-box and WD-40 domain protein 2 | -1.367773437 | 0.037162872 |
| *Chd6* | chromodomain helicase DNA binding protein 6 | -1.369433594 | 0.001468312 |
| *Setdb2* | SET domain, bifurcated 2 | -1.370019531 | 0.031327837 |
| *Pmf1* | polyamine-modulated factor 1 | -1.370117188 | 0.042499104 |
| *Smc2* | structural maintenance of chromosomes 2 | -1.3703125 | 0.011614951 |
| *Gm10012* | cytochrome c oxidase, subunit VIIc pseudogene | -1.37890625 | 0.007709592 |
| *Akna* | AT-hook transcription factor | -1.380273438 | 0.001203224 |
| *Yeats4* | YEATS domain containing 4 | -1.380664063 | 0.009833609 |
| *Fancl* | Fanconi anemia, complementation group L | -1.381054688 | 0.00054197 |
| *Nup160* | nucleoporin 160 | -1.386523438 | 0.048077994 |
| *Tubgcp2* | tubulin, gamma complex associated protein 2 | -1.387109375 | 0.010099634 |
| *Hebp1* | heme binding protein 1 | -1.393945312 | 0.000467326 |
| *Gclc* | glutamate-cysteine ligase, catalytic subunit | -1.396484375 | 0.004482778 |
| *Mboat2* | membrane bound O-acyltransferase domain containing 2 | -1.39765625 | 0.000796477 |
| *Ppm1g* | protein phosphatase 1G (formerly 2C), magnesium-dependent, gamma isoform | -1.397851563 | 0.001297354 |
| *Kif14* | kinesin family member 14 | -1.39921875 | 0.008745257 |
| *Dnmt1* | DNA methyltransferase (cytosine-5) 1 | -1.40022089 | 0.007096831 |
| *Car1* | carbonic anhydrase 1 | -1.400390625 | 0.015440981 |
| *Lrrc8c* | leucine rich repeat containing 8 family, member C | -1.402148438 | 0.009807527 |
| *Rrm2* | ribonucleotide reductase M2 | -1.40234375 | 0.012633988 |
| *Extl3* | exostoses (multiple)-like 3 | -1.406445312 | 0.017334719 |
| *Ube2t* | ubiquitin-conjugating enzyme E2T | -1.409472656 | 0.00069984 |
| *Nsd2* | nuclear receptor binding SET domain protein 2 | -1.409570313 | 0.001924663 |
| *Yaf2* | YY1 associated factor 2 | -1.410253906 | 0.00191975 |
| *Nde1* | nudE neurodevelopment protein 1 | -1.412109375 | 0.02043433 |
| *Nmral1* | NmrA-like family domain containing 1 | -1.415039063 | 0.005504369 |
| *Tyms* | thymidylate synthase | -1.416796875 | 0.013264435 |
| *Sirt1* | sirtuin 1 | -1.418164063 | 0.004455264 |
| *Abca3* | ATP-binding cassette, sub-family A (ABC1), member 3 | -1.4203125 | 0.006127037 |
| *Zgrf1* | zinc finger, GRF-type containing 1 | -1.424414063 | 7.46924E-05 |
| *Pank2* | pantothenate kinase 2 | -1.425 | 0.000634074 |
| *Sap18* | Sin3-associated polypeptide 18 | -1.426953125 | 0.030292661 |
| *Neat1* | nuclear paraspeckle assembly transcript 1 (non-protein coding) | -1.427929688 | 0.005206323 |
| *Fancd2* | Fanconi anemia, complementation group D2 | -1.428125 | 0.030605506 |
| *Gm13710* | predicted gene 13710 | -1.428222656 | 0.02166121 |
| *Tmem14c* | transmembrane protein 14C | -1.42890625 | 0.019011182 |
| *Baz1a* | bromodomain adjacent to zinc finger domain 1A | -1.4296875 | 0.002221411 |
| *Pds5b* | PDS5 cohesin associated factor B | -1.429785156 | 0.011724616 |
| *Atp2b1* | ATPase, Ca++ transporting, plasma membrane 1 | -1.430664063 | 0.017088132 |
| *Erh* | ERH mRNA splicing and mitosis factor | -1.43125 | 0.00630225 |
| *Knl1* | kinetochore scaffold 1 | -1.431640625 | 0.000692379 |
| *Snrpe* | small nuclear ribonucleoprotein E | -1.43203125 | 0.025417067 |
| *Gabpb2* | GA repeat binding protein, beta 2 | -1.43984375 | 0.043767623 |
| *A630089N07Rik* | RIKEN cDNA A630089N07 gene | -1.440917969 | 0.008138421 |
| *Zdhhc15* | zinc finger, DHHC domain containing 15 | -1.442089844 | 6.61793E-05 |
| *Dck* | deoxycytidine kinase | -1.444726563 | 0.007994526 |
| *Tmem107* | transmembrane protein 107 | -1.4453125 | 0.001611662 |
| *Smchd1* | SMC hinge domain containing 1 | -1.4484375 | 0.000627467 |
| *Trpm7* | transient receptor potential cation channel, subfamily M, member 7 | -1.449804688 | 0.016859385 |
| *Smc3* | structural maintenance of chromosomes 3 | -1.451757812 | 0.000522383 |
| *Ticrr* | TOPBP1-interacting checkpoint and replication regulator | -1.451757813 | 0.037988142 |
| *Hist2h2bb* | histone cluster 2, H2bb | -1.451953125 | 0.03288415 |
| *Nucks1* | nuclear casein kinase and cyclin-dependent kinase substrate 1 | -1.452734375 | 0.031569074 |
| *Gm15199* | predicted gene 15199 | -1.455566406 | 0.034819435 |
| *Exo1* | exonuclease 1 | -1.456152344 | 0.005144738 |
| *Cdc25a* | cell division cycle 25A | -1.459375 | 0.00379002 |
| *Prdx4* | peroxiredoxin 4 | -1.459375 | 0.007484885 |
| *Megf9* | multiple EGF-like-domains 9 | -1.4609375 | 0.007332123 |
| *Brip1* | BRCA1 interacting protein C-terminal helicase 1 | -1.464160156 | 0.003582834 |
| *Fen1* | flap structure specific endonuclease 1 | -1.465234375 | 0.003093568 |
| *Tmem206* | transmembrane protein 206 | -1.468457031 | 8.77132E-05 |
| *Cdt1* | chromatin licensing and DNA replication factor 1 | -1.46875 | 0.002961777 |
| *Taf1* | TATA-box binding protein associated factor 1 | -1.46875 | 0.022115425 |
| *Rfwd3* | ring finger and WD repeat domain 3 | -1.470898438 | 0.010554693 |
| *Plpp1* | phospholipid phosphatase 1 | -1.472460938 | 0.001149946 |
| *Ncapg* | non-SMC condensin I complex, subunit G | -1.472460938 | 0.039191135 |
| *Cd59b* | CD59b antigen | -1.480664063 | 0.012330357 |
| *Ccnb1* | cyclin B1 | -1.480859375 | 0.03652716 |
| *Tinf2* | Terf1 (TRF1)-interacting nuclear factor 2 | -1.484375 | 0.02215331 |
| *Dars2* | aspartyl-tRNA synthetase 2 (mitochondrial) | -1.485742187 | 0.003143387 |
| *Kif4* | kinesin family member 4 | -1.487792969 | 0.047860576 |
| *Zranb3* | zinc finger, RAN-binding domain containing 3 | -1.490234375 | 0.001568437 |
| *Nup155* | nucleoporin 155 | -1.492773438 | 0.006781945 |
| *Nkg7* | natural killer cell group 7 sequence | -1.493652344 | 0.025647455 |
| *Alyref* | Aly/REF export factor | -1.498632813 | 0.005176122 |
| *Ska3* | spindle and kinetochore associated complex subunit 3 | -1.50078125 | 0.008004975 |
| *9630013D21Rik* | RIKEN cDNA 9630013D21 gene | -1.502246094 | 0.01078007 |
| *Gm12247* | predicted gene 12247 | -1.507617188 | 0.012662826 |
| *1190002N15Rik* | RIKEN cDNA 1190002N15 gene | -1.50859375 | 0.006023839 |
| *Meaf6* | MYST/Esa1-associated factor 6 | -1.508691406 | 0.005872609 |
| *Gstt2* | glutathione S-transferase, theta 2 | -1.512011719 | 0.010111413 |
| *Far1* | fatty acyl CoA reductase 1 | -1.51484375 | 0.007040657 |
| *Npat* | nuclear protein in the AT region | -1.516015625 | 0.039041164 |
| *Mastl* | microtubule associated serine/threonine kinase-like | -1.5171875 | 0.00084256 |
| *Gins4* | GINS complex subunit 4 (Sld5 homolog) | -1.517773438 | 0.004228285 |
| *Lmnb1* | lamin B1 | -1.51953125 | 0.012630553 |
| *Sgk3* | serum/glucocorticoid regulated kinase 3 | -1.521875 | 0.000450892 |
| *Smim11* | small integral membrane protein 11 | -1.524804688 | 0.001456363 |
| *Kif18a* | kinesin family member 18A | -1.525585938 | 0.048344048 |
| *Cep290* | centrosomal protein 290 | -1.525976563 | 8.20937E-05 |
| *Kif11* | kinesin family member 11 | -1.526171877 | 0.002409601 |
| *Casp8ap2* | caspase 8 associated protein 2 | -1.526660156 | 0.012393746 |
| *Nme3* | NME/NM23 nucleoside diphosphate kinase 3 | -1.53125 | 0.000341606 |
| *Med6* | mediator complex subunit 6 | -1.531933594 | 0.034719503 |
| *Ccne2* | cyclin E2 | -1.534375 | 0.000526024 |
| *Kbtbd6* | kelch repeat and BTB (POZ) domain containing 6 | -1.536816406 | 0.00243457 |
| *Slc29a1* | solute carrier family 29 (nucleoside transporters), member 1 | -1.538476563 | 0.002724059 |
| *Rac2* | RAS-related C3 botulinum substrate 2 | -1.543554688 | 0.02473835 |
| *LOC102635682* | uncharacterized LOC102635682 | -1.545117188 | 0.031676547 |
| *Cenpe* | centromere protein E | -1.54765625 | 0.023288939 |
| *D430020J02Rik* | RIKEN cDNA D430020J02 gene | -1.549804688 | 0.003090193 |
| *Dclre1a* | DNA cross-link repair 1A | -1.55234375 | 0.002040263 |
| *Rfc5* | replication factor C (activator 1) 5 | -1.552734375 | 0.003089274 |
| *Tfb1m* | transcription factor B1, mitochondrial | -1.553125 | 0.004071864 |
| *Fam69a* | family with sequence similarity 69, member A | -1.553222656 | 0.025376446 |
| *Afmid* | arylformamidase | -1.553515857 | 0.000533459 |
| *Rdm1* | RAD52 motif 1 | -1.559570313 | 0.000709163 |
| *Ncapd2* | non-SMC condensin I complex, subunit D2 | -1.564257813 | 0.000808415 |
| *Nup188* | nucleoporin 188 | -1.56640625 | 0.005038655 |
| *Mrpl14* | mitochondrial ribosomal protein L14 | -1.571191406 | 0.017830373 |
| *Hirip3* | HIRA interacting protein 3 | -1.572070313 | 0.000778238 |
| *Bub1* | BUB1, mitotic checkpoint serine/threonine kinase | -1.577734375 | 0.013887128 |
| *Ppwd1* | peptidylprolyl isomerase domain and WD repeat containing 1 | -1.579296875 | 0.0035047 |
| *Pkhd1l1* | polycystic kidney and hepatic disease 1-like 1 | -1.580484859 | 0.00070285 |
| *Dleu2* | deleted in lymphocytic leukemia, 2 | -1.581054688 | 0.001320571 |
| *E2f7* | E2F transcription factor 7 | -1.582421875 | 0.046298365 |
| *Lpcat1* | lysophosphatidylcholine acyltransferase 1 | -1.583789062 | 0.031157999 |
| *Pidd1* | p53 induced death domain protein 1 | -1.583984375 | 0.000451895 |
| *Tmco6* | transmembrane and coiled-coil domains 6 | -1.584179688 | 0.000834066 |
| *Mir1957a* | microRNA 1957a | -1.5890625 | 0.019945179 |
| *Gm42111* | predicted gene, 42111 | -1.590429688 | 0.029020308 |
| *Tubb5* | tubulin, beta 5 class I | -1.593554688 | 0.031157999 |
| *Usp37* | ubiquitin specific peptidase 37 | -1.601953125 | 0.009111622 |
| *Pdzd8* | PDZ domain containing 8 | -1.602832031 | 0.011298774 |
| *Chaf1a* | chromatin assembly factor 1, subunit A (p150) | -1.603515625 | 0.019932991 |
| *Srsf10* | serine/arginine-rich splicing factor 10 | -1.603710938 | 0.019537449 |
| *Ubr7* | ubiquitin protein ligase E3 component n-recognin 7 (putative) | -1.604589844 | 0.010402177 |
| *Arpc5l* | actin related protein 2/3 complex, subunit 5-like | -1.605273438 | 0.007509856 |
| *Nup210* | nucleoporin 210 | -1.606054688 | 0.004590491 |
| *Pole2* | polymerase (DNA directed), epsilon 2 (p59 subunit) | -1.607324219 | 0.005115201 |
| *Aspm* | abnormal spindle microtubule assembly | -1.608203125 | 0.00383153 |
| *Ssrp1* | structure specific recognition protein 1 | -1.610449219 | 0.009524043 |
| *Tmem209* | transmembrane protein 209 | -1.610742188 | 0.019267291 |
| *Zfp367* | zinc finger protein 367 | -1.612695313 | 0.003452968 |
| *Phf12* | PHD finger protein 12 | -1.613671875 | 0.015457576 |
| *Tac2* | tachykinin 2 | -1.613769531 | 0.036001557 |
| *Cmc2* | COX assembly mitochondrial protein 2 | -1.617382812 | 0.013421592 |
| *Pds5a* | PDS5 cohesin associated factor A | -1.6203125 | 0.042026088 |
| *Prc1* | protein regulator of cytokinesis 1 | -1.6234375 | 0.001452862 |
| *Ints7* | integrator complex subunit 7 | -1.623925781 | 0.007208898 |
| *Rnf212* | ring finger protein 212 | -1.624902344 | 0.002142409 |
| *Manf* | mesencephalic astrocyte-derived neurotrophic factor | -1.627734375 | 0.013871252 |
| *Gnpda1* | glucosamine-6-phosphate deaminase 1 | -1.629101563 | 0.00173769 |
| *Dlgap5* | DLG associated protein 5 | -1.63125 | 0.029751749 |
| *Dgcr8* | DGCR8, microprocessor complex subunit | -1.633007813 | 0.00209399 |
| *Fcho1* | FCH domain only 1 | -1.634765625 | 0.006814229 |
| *Lrr1* | leucine rich repeat protein 1 | -1.635449219 | 0.003442251 |
| *Erg28* | ergosterol biosynthesis 28 | -1.640039062 | 0.003310793 |
| *Arl6ip6* | ADP-ribosylation factor-like 6 interacting protein 6 | -1.640234375 | 0.013700835 |
| *Rpa1* | replication protein A1 | -1.6421875 | 0.00630176 |
| *Hist1h1a* | histone cluster 1, H1a | -1.645800781 | 0.016282304 |
| *C1qtnf12* | C1q and tumor necrosis factor related 12 | -1.64765625 | 0.000274499 |
| *Nbn* | nibrin | -1.652734375 | 0.010679405 |
| *Zfp808* | zinc finger protein 80 | -1.657910156 | 0.014097127 |
| *Dhfr* | dihydrofolate reductase | -1.667578125 | 0.004484083 |
| *Atad5* | ATPase family, AAA domain containing 5 | -1.670898438 | 0.003784268 |
| *Cenph* | centromere protein H | -1.672167969 | 0.002873412 |
| *A730089K16Rik* | RIKEN cDNA A730089K16 gene | -1.674707031 | 0.000101153 |
| *Cks1b* | CDC28 protein kinase 1b | -1.678125 | 0.008020333 |
| *Nemp1* | nuclear envelope integral membrane protein 1 | -1.683984375 | 0.034043559 |
| *Fam205a1* | family with sequence similarity 205, member A1 | -1.684277344 | 0.015799921 |
| *Topbp1* | topoisomerase (DNA) II binding protein 1 | -1.6859375 | 0.004580978 |
| *Eldr* | Egfr long non-coding downstream RNA | -1.686230469 | 0.000109043 |
| *Tmpo* | thymopoietin | -1.687695313 | 0.013490755 |
| *Ankrd28* | ankyrin repeat domain 28 | -1.6890625 | 0.002623974 |
| *Grap2* | GRB2-related adaptor protein 2 | -1.689453125 | 0.000250783 |
| *Pold3* | polymerase (DNA-directed), delta 3, accessory subunit | -1.693554688 | 0.000771669 |
| *Tfdp1* | transcription factor Dp 1 | -1.696484375 | 0.022759803 |
| *Cenpl* | centromere protein L | -1.696679688 | 0.015411102 |
| *Stag1* | stromal antigen 1 | -1.70078125 | 0.006154274 |
| *Uhrf1* | ubiquitin-like, containing PHD and RING finger domains, 1 | -1.708203125 | 0.024605903 |
| *Mad2l1* | MAD2 mitotic arrest deficient-like 1 | -1.711328125 | 0.034962155 |
| *Kntc1* | kinetochore associated 1 | -1.711523438 | 0.00108597 |
| *Etaa1* | Ewing tumor-associated antigen 1 | -1.712695313 | 0.000275782 |
| *Casp2* | caspase 2 | -1.71484375 | 0.003090193 |
| *Cdc45* | cell division cycle 45 | -1.71640625 | 0.008620376 |
| *Agtr1a* | angiotensin II receptor, type 1a | -1.7203125 | 0.025706863 |
| *Rpa3* | replication protein A3 | -1.720605469 | 0.004330045 |
| *Cnnm2* | cyclin M2 | -1.72109375 | 0.001599276 |
| *Pcna* | proliferating cell nuclear antigen | -1.725 | 0.042964558 |
| *Sri* | sorcin | -1.72578125 | 8.90197E-05 |
| *Cenpn* | centromere protein N | -1.727148438 | 0.041971174 |
| *Hmgb2* | high mobility group box 2 | -1.729296875 | 0.004846365 |
| *Dna2* | DNA replication helicase/nuclease 2 | -1.730273438 | 0.000387557 |
| *Baz1b* | bromodomain adjacent to zinc finger domain, 1B | -1.73203125 | 0.008222087 |
| *Orc6* | origin recognition complex, subunit 6 | -1.733007813 | 0.008373017 |
| *Tmem230* | transmembrane protein 230 | -1.733398438 | 0.011643699 |
| *Hist1h2bn* | histone cluster 1, H2bn | -1.734375 | 0.014660245 |
| *Rad9a* | RAD9 checkpoint clamp component A | -1.741601563 | 0.009367437 |
| *Parvb* | parvin, beta | -1.744726563 | 0.000340933 |
| *Ncapd3* | non-SMC condensin II complex, subunit D3 | -1.746875 | 0.007102588 |
| *Ctdspl2* | CTD (carboxy-terminal domain, RNA polymerase II, polypeptide A) small phosphatase like 2 | -1.748632813 | 0.007469147 |
| *6030468B19Rik* | RIKEN cDNA 6030468B19 gene | -1.749023438 | 0.003878713 |
| *Mir3097* | microRNA 3097 | -1.749316406 | 0.017463944 |
| *Gna11* | guanine nucleotide binding protein, alpha 11 | -1.751171875 | 0.000533107 |
| *Polr3b* | polymerase (RNA) III (DNA directed) polypeptide B | -1.752929688 | 0.000345063 |
| *Nup133* | nucleoporin 133 | -1.755078125 | 0.005558173 |
| *Hpf1* | histone PARylation factor 1 | -1.75546875 | 0.029218438 |
| *Hmmr* | hyaluronan mediated motility receptor (RHAMM) | -1.75546875 | 0.015813473 |
| *Kif22* | kinesin family member 22 | -1.756054688 | 0.014713893 |
| *Hist2h2ab* | histone cluster 2, H2ab | -1.758984375 | 0.005481635 |
| *Hells* | helicase, lymphoid specific | -1.761132813 | 0.00146749 |
| *Rfc1* | replication factor C (activator 1) 1 | -1.763085938 | 0.00024311 |
| *Fam107b* | family with sequence similarity 107, member B | -1.764453125 | 0.002273649 |
| *Daglb* | diacylglycerol lipase, beta | -1.765527344 | 0.001060979 |
| *Rif1* | replication timing regulatory factor 1 | -1.773632813 | 0.004893912 |
| *Dek* | DEK oncogene (DNA binding) | -1.779296875 | 0.003169091 |
| *Nono* | non-POU-domain-containing, octamer binding protein | -1.781835937 | 0.035882193 |
| *Terf2* | telomeric repeat binding factor 2 | -1.78359375 | 0.001824128 |
| *Dnajc9* | DnaJ heat shock protein family (Hsp40) member C9 | -1.784960938 | 0.011444736 |
| *Selenbp1* | selenium binding protein 1 | -1.785546875 | 0.004569484 |
| *Mxd3* | Max dimerization protein 3 | -1.7859375 | 0.004497353 |
| *Polq* | polymerase (DNA directed), theta | -1.793945313 | 0.000956387 |
| *Prim2* | DNA primase, p58 subunit | -1.794140625 | 0.001730078 |
| *Rbl1* | retinoblastoma-like 1 (p107) | -1.79453125 | 0.001417877 |
| *Ifitm3* | interferon induced transmembrane protein 3 | -1.801757812 | 0.022588966 |
| *Kif18b* | kinesin family member 18B | -1.8046875 | 0.014455632 |
| *Hnrnpa3* | heterogeneous nuclear ribonucleoprotein A3 | -1.80625 | 0.016762548 |
| *Pola1* | polymerase (DNA directed), alpha 1 | -1.81015625 | 0.001001106 |
| *Cdca5* | cell division cycle associated 5 | -1.812695313 | 0.000985496 |
| *Kpna3* | karyopherin (importin) alpha 3 | -1.81328125 | 0.011239208 |
| *Pprc1* | peroxisome proliferative activated receptor, gamma, coactivator-related 1 | -1.816699219 | 0.000205583 |
| *Mdc1* | mediator of DNA damage checkpoint 1 | -1.819140625 | 0.004948254 |
| *Brca1* | breast cancer 1, early onset | -1.826660156 | 0.00820245 |
| *Cenpk* | centromere protein K | -1.826757813 | 0.000618163 |
| *Donson* | downstream neighbor of SON | -1.831054688 | 0.015516654 |
| *Hist1h2bk* | histone cluster 1, H2bk | -1.838085938 | 0.039760821 |
| *Larp7* | La ribonucleoprotein domain family, member 7 | -1.842578125 | 0.002058912 |
| *Cdca7* | cell division cycle associated 7 | -1.842773438 | 0.003693276 |
| *Gfi1b* | growth factor independent 1B | -1.842773438 | 0.037962002 |
| *Haspin* | histone H3 associated protein kinase | -1.845019531 | 0.008138421 |
| *Pold1* | polymerase (DNA directed), delta 1, catalytic subunit | -1.85546875 | 5.60235E-05 |
| *Rad54l* | RAD54 like (S. cerevisiae) | -1.858203125 | 0.000239037 |
| *Mcm7* | minichromosome maintenance complex component 7 | -1.87421875 | 0.015727175 |
| *Cse1l* | chromosome segregation 1-like (S. cerevisiae) | -1.875488281 | 0.003702034 |
| *Atad2* | ATPase family, AAA domain containing 2 | -1.879101563 | 0.003200475 |
| *Pkmyt1* | protein kinase, membrane associated tyrosine/threonine 1 | -1.879882813 | 0.002295025 |
| *BC052040* | cDNA sequence BC052040 | -1.883984375 | 0.006201096 |
| *Tmem120b* | transmembrane protein 120B | -1.888476563 | 0.043812376 |
| *Anln* | anillin, actin binding protein | -1.890625 | 0.001522598 |
| *Birc5* | baculoviral IAP repeat-containing 5 | -1.891796875 | 0.009394388 |
| *Spc25* | SPC25, NDC80 kinetochore complex component, homolog (S. cerevisiae) | -1.895507812 | 0.005956091 |
| *Xndc1* | Xrcc1 N-terminal domain containing 1 | -1.900683594 | 0.01915761 |
| *Cit* | citron | -1.909960938 | 0.003228517 |
| *Tspan32* | tetraspanin 32 | -1.911914063 | 0.009461438 |
| *Prr11* | proline rich 11 | -1.915234375 | 0.006667293 |
| *Skp2* | S-phase kinase-associated protein 2 (p45) | -1.928320313 | 0.003442251 |
| *Hist1h2bf* | histone cluster 1, H2bf | -1.930859375 | 0.005813786 |
| *Hras* | Harvey rat sarcoma virus oncogene | -1.93125 | 0.000223289 |
| *Samd1* | sterile alpha motif domain containing 1 | -1.938085938 | 0.006471762 |
| *Hist2h2ac* | histone cluster 2, H2ac | -1.95 | 0.043037016 |
| *Nup85* | nucleoporin 85 | -1.950976563 | 0.001206093 |
| *Ssna1* | SS nuclear autoantigen 1 | -1.952441406 | 0.002078193 |
| *Gen1* | GEN1, Holliday junction 5_ flap endonuclease | -1.95390625 | 0.007940739 |
| *Asf1b* | anti-silencing function 1B histone chaperone | -1.958007813 | 0.016754187 |
| *Cdk2* | cyclin-dependent kinase 2 | -1.963867188 | 0.011460361 |
| *Gins1* | GINS complex subunit 1 (Psf1 homolog) | -1.966015625 | 0.035847073 |
| *Chek1* | checkpoint kinase 1 | -1.966308594 | 0.003601113 |
| *Mis12* | MIS12 kinetochore complex component | -1.967773438 | 0.016511883 |
| *Cd63* | CD63 antigen | -1.969921875 | 0.027017614 |
| *Grcc10* | gene rich cluster, C10 gene | -1.972851563 | 1.3268E-05 |
| *Agfg2* | ArfGAP with FG repeats 2 | -1.975 | 0.007412066 |
| *Lpcat3* | lysophosphatidylcholine acyltransferase 3 | -1.977343749 | 0.000231695 |
| *Brca2* | breast cancer 2, early onset | -1.979296875 | 0.000408864 |
| *Eri1* | exoribonuclease 1 | -1.989257813 | 0.016012144 |
| *Stil* | Scl/Tal1 interrupting locus | -1.992578125 | 0.006645089 |
| *Mcm2* | minichromosome maintenance complex component 2 | -1.994726563 | 0.004632921 |
| *Slbp* | stem-loop binding protein | -2.009570312 | 0.01549997 |
| *Aurkb* | aurora kinase B | -2.0140625 | 0.020656584 |
| *Ska2* | spindle and kinetochore associated complex subunit 2 | -2.014550781 | 0.006685013 |
| *Suox* | sulfite oxidase | -2.01484375 | 8.26104E-05 |
| *Smarcc2* | SWI/SNF related, matrix associated, actin dependent regulator of chromatin, subfamily c, member 2 | -2.022851563 | 0.002094218 |
| *Wdr76* | WD repeat domain 76 | -2.030664063 | 0.010595084 |
| *Smc6* | structural maintenance of chromosomes 6 | -2.03203125 | 0.000396401 |
| *Ccnf* | cyclin F | -2.034960938 | 0.012330357 |
| *Dnajb3* | DnaJ heat shock protein family (Hsp40) member B3 | -2.03828125 | 0.013342537 |
| *Cbx5* | chromobox 5 | -2.041113281 | 0.007893984 |
| *Dtwd1* | DTW domain containing 1 | -2.058496094 | 7.386E-05 |
| *Pola2* | polymerase (DNA directed), alpha 2 | -2.0640625 | 0.001099787 |
| *Clspn* | claspin | -2.065039063 | 0.005426777 |
| *Abcg4* | ATP binding cassette subfamily G member 4 | -2.070117188 | 0.005154523 |
| *Chst10* | carbohydrate sulfotransferase 10 | -2.078515625 | 0.029269882 |
| *Pask* | PAS domain containing serine/threonine kinase | -2.083691406 | 0.001444625 |
| *Gstt1* | glutathione S-transferase, theta 1 | -2.089257813 | 0.001023817 |
| *Rad51* | RAD51 recombinase | -2.091308594 | 0.004249524 |
| *Tk1* | thymidine kinase 1 | -2.093164063 | 0.001569687 |
| *Msh6* | mutS homolog 6 | -2.093554688 | 0.010187115 |
| *Rfc3* | replication factor C (activator 1) 3 | -2.095996094 | 0.006571596 |
| *A430110L20Rik* | RIKEN cDNA A430110L20 gene | -2.099023438 | 0.003295623 |
| *Slc9a8* | solute carrier family 9 (sodium/hydrogen exchanger), member 8 | -2.100195313 | 5.16966E-05 |
| *Cxcr4* | chemokine (C-X-C motif) receptor 4 | -2.104882813 | 0.008968644 |
| *Srsf7* | serine/arginine-rich splicing factor 7 | -2.10625 | 0.017463944 |
| *A430005L14Rik* | RIKEN cDNA A430005L14 gene | -2.115625 | 0.010901761 |
| *Kif15* | kinesin family member 15 | -2.121875 | 0.001999939 |
| *Nuf2* | NUF2, NDC80 kinetochore complex component | -2.133789063 | 0.042764451 |
| *Plk4* | polo like kinase 4 | -2.140039063 | 0.002817367 |
| *Dscc1* | DNA replication and sister chromatid cohesion 1 | -2.146972656 | 0.000658561 |
| *Gm10371* | predicted gene 10371 | -2.162109375 | 0.014106685 |
| *Gp9* | glycoprotein 9 (platelet) | -2.162695313 | 0.000841864 |
| *Pbk* | PDZ binding kinase | -2.165429688 | 0.006730981 |
| *Rrm1* | ribonucleotide reductase M1 | -2.179492188 | 0.015309415 |
| *Chaf1b* | chromatin assembly factor 1, subunit B (p60) | -2.179882813 | 0.009649653 |
| *Top2a* | topoisomerase (DNA) II alpha | -2.190039063 | 0.003041278 |
| *Ccdc6* | coiled-coil domain containing 6 | -2.196484375 | 5.2564E-06 |
| *Neil3* | nei like 3 (E. coli) | -2.196875 | 0.011358359 |
| *Rfc2* | replication factor C (activator 1) 2 | -2.201367188 | 0.000512457 |
| *Ncaph* | non-SMC condensin I complex, subunit H | -2.205078125 | 0.007933139 |
| *Rad51ap1* | RAD51 associated protein 1 | -2.212109375 | 0.002305453 |
| *E2f8* | E2F transcription factor 8 | -2.230664063 | 0.02450199 |
| *Tmem179b* | transmembrane protein 179B | -2.237109375 | 0.001030357 |
| *Scarb1* | scavenger receptor class B, member 1 | -2.241015625 | 0.000818279 |
| *Rnf168* | ring finger protein 168 | -2.250585938 | 0.000308384 |
| *Fam111a* | family with sequence similarity 111, member A | -2.259179688 | 0.000790238 |
| *Lig1* | ligase I, DNA, ATP-dependent | -2.260546875 | 0.007888691 |
| *Rpa2* | replication protein A2 | -2.263183594 | 0.003063462 |
| *Prim1* | DNA primase, p49 subunit | -2.26484375 | 0.034844725 |
| *H2afv* | H2A histone family, member V | -2.274609375 | 0.000195751 |
| *Dtymk* | deoxythymidylate kinase | -2.280273438 | 0.002474314 |
| *Mybl2* | myeloblastosis oncogene-like 2 | -2.285742188 | 0.007407671 |
| *Tipin* | timeless interacting protein | -2.313085938 | 0.029181035 |
| *Kifc1* | kinesin family member C1 | -2.316601563 | 0.028155712 |
| *Cdca4* | cell division cycle associated 4 | -2.322851563 | 0.001357269 |
| *Mcm3* | minichromosome maintenance complex component 3 | -2.33359375 | 0.016084235 |
| *Lrrc39* | leucine rich repeat containing 39 | -2.336914063 | 2.42669E-05 |
| *Lage3* | L antigen family, member 3 | -2.351660156 | 0.013526324 |
| *Acp5* | acid phosphatase 5, tartrate resistant | -2.354101563 | 0.000126105 |
| *Mcm4* | minichromosome maintenance complex component 4 | -2.359960938 | 0.003853184 |
| *Mms22l* | MMS22-like, DNA repair protein | -2.3828125 | 0.000473772 |
| *Mcm6* | minichromosome maintenance complex component 6 | -2.388085938 | 0.005890933 |
| *Pole* | polymerase (DNA directed), epsilon | -2.403710938 | 0.004019497 |
| *Erdr1* | erythroid differentiation regulator 1 | -2.40859375 | 0.006244932 |
| *Dtl* | denticleless E3 ubiquitin protein ligase | -2.436132813 | 0.008727015 |
| *Cdc6* | cell division cycle 6 | -2.44296875 | 0.002597699 |
| *Snora23* | small nucleolar RNA, H/ACA box 23 | -2.461425781 | 0.001273366 |
| *Ncapg2* | non-SMC condensin II complex, subunit G2 | -2.482226563 | 0.012335559 |
| *Wdhd1* | WD repeat and HMG-box DNA binding protein 1 | -2.488085938 | 0.00057874 |
| *Rfc4* | replication factor C (activator 1) 4 | -2.501855469 | 0.019565347 |
| *Cdk1* | cyclin-dependent kinase 1 | -2.551171875 | 0.009814947 |
| *Mcm5* | minichromosome maintenance complex component 5 | -2.566601562 | 0.010298788 |
| *Ccne1* | cyclin E1 | -2.596289063 | 0.002667108 |
| *Tcf19* | transcription factor 19 | -2.602734375 | 0.003840726 |
| *Fads1* | fatty acid desaturase 1 | -2.62421875 | 0.000193814 |
| *Zfp39* | zinc finger protein 39 | -2.625585938 | 8.54135E-05 |
| *Hat1* | histone aminotransferase 1 | -2.625585938 | 0.01487938 |
| *Trim34a* | tripartite motif-containing 34A | -2.648242188 | 0.000238477 |
| *Cacna1g* | calcium channel, voltage-dependent, T type, alpha 1G subunit | -2.653125 | 0.000322617 |
| *Mpo* | myeloperoxidase | -2.666992188 | 0.032743731 |
| *Fbxo5* | F-box protein 5 | -2.66953125 | 0.033991594 |
| *Aunip* | aurora kinase A and ninein interacting protein | -2.670605469 | 0.001603553 |
| *Gmnn* | geminin | -2.723828125 | 0.007194658 |
| *Slc6a20a* | solute carrier family 6 (neurotransmitter transporter), member 20A | -2.74570149 | 0.000120835 |
| *Elane* | elastase, neutrophil expressed | -2.747851563 | 0.032791637 |
| *LOC102633666* | uncharacterized LOC102633666 | -2.756054688 | 0.003143387 |
| *Camp* | cathelicidin antimicrobial peptide | -2.787695312 | 0.040601681 |
| *Pacs2* | phosphofurin acidic cluster sorting protein 2 | -2.812695313 | 2.83595E-05 |
| *4930430F08Rik* | RIKEN cDNA 4930430F08 gene | -2.816308594 | 0.000129164 |
| *Fignl1* | fidgetin-like 1 | -2.902148438 | 0.001321259 |
| *Ssh1* | slingshot protein phosphatase 1 | -3.156445312 | 0.000116378 |
| *Ighm* | immunoglobulin heavy constant mu | -3.845019531 | 0.013115785 |
